# Impact of the diagnostic label for a low-risk prostate lesion: protocol for two online factorial randomised experiments

**DOI:** 10.1136/bmjopen-2024-085947

**Published:** 2024-08-09

**Authors:** James Bullen, Brooke Nickel, Kirsten McCaffery, Timothy J Wilt, Jenna Smith, Farzaneh Boroumand, Lisa Parker, Jeremy Millar, John Brandt Brodersen, Philipp Dahm, Brett Delahunt, Murali Varma, Paul Glasziou, Andrew Warden, Lawrence Diller, Larry Billington, Christo van Rensburg, Katy Bell

**Affiliations:** 1 School of Public Health, University of Sydney, Sydney, New South Wales, Australia; 2 Sydney Health Literacy Lab, School of Public Health, University of Sydney, Sydney, New South Wales, Australia; 3 Wiser Healthcare Research Collaboration, Sydney, New South Wales, Australia; 4 Center for Chronic Disease Outcomes Research and Minneapolis VA High Value Care Initiative, Minneapolis VA Health Care System, Minneapolis, Minnesota, USA; 5 Department of Medicine, Section of General Internal Medicine, University of Minnesota Twin Cities, Minneapolis, Minnesota, USA; 6 School of Pharmacy, Faculty of Medicine and Health, Charles Perkins Centre, University of Sydney, Sydney, New South Wales, Australia; 7 Department of Radiation Oncology, Royal North Shore Hospital, NSW Health, Sydney, New South Wales, Australia; 8 Radiation Oncology, Alfred Health, Melbourne, Victoria, Australia; 9 School of Public Health and Preventive Medicine, Faculty of Medicine, Nursing and Health Sciences, Monash University, Melbourne, Victoria, Australia; 10 Centre of General Practice, Department of Public Health & Research Unit for General Practice, Region Zealand, University of Copenhagen, Copenhagen, Denmark; 11 Research Unit for General Practice, Department of Community Medicine, UiT The Arctic University of Norway Faculty of Health Sciences, Tromso, Norway; 12 Department of Urology, University of Minnesota, Minneapolis, Minnesota, USA; 13 Urology Section, Minneapolis Veterans Administration Health System, Minneapolis, Minnesota, USA; 14 Wellington School of Medicine and Health Sciences, University of Otago Wellington, Wellington, New Zealand; 15 Department of Cellular Pathology, University Hospital of Wales, Cardiff, UK; 16 Institute for Evidence-Based Healthcare, Bond University, Gold Coast, Queensland, Australia; 17 Health Consumers New South Wales, Sydney, New South Wales, Australia

**Keywords:** Prostatic Neoplasms, Adverse events, Clinical Decision-Making, Clinical Trial, Surgical pathology, Patient-Centered Care

## Abstract

**Introduction:**

Many types of prostate cancer present minimal risk to a man’s lifespan or well-being, but existing terminology makes it difficult for men to distinguish these from high-risk prostate cancers. This study aims to explore whether using an alternative label for low-risk prostate cancer influences management choice and anxiety levels among Australian men and their partners.

**Methods and analysis:**

We will run two separate studies for Australian men and Australian women with a male partner. Both studies are between-subjects factorial (3×2) randomised online hypothetical experiments. Following consent, eligible participants will be randomised 1:1:1 to three labels: ‘low-risk prostate cancer, Gleason Group 1’, ‘low-risk prostate neoplasm’ or ‘low-risk prostate lesion’. Participants will then undergo a second randomisation step with 1:1 allocation to the provision of detailed information on the benefits and harms of different management choices versus the provision of less detailed information about management choices. The required sample sizes are 1290 men and 1410 women. The primary outcome is the participant choice of their preferred management strategy: no immediate treatment (prostate-specific antigen (PSA)-based monitoring or active surveillance using PSA, MRI, biopsy with delayed treatment for disease progression) versus immediate treatment (prostatectomy or radiation therapy). Secondary outcomes include preferred management choice (from the four options listed above), diagnosis anxiety, management choice anxiety and management choice at a later time point (for participants who initially choose a monitoring strategy).

**Ethics and dissemination:**

Ethics approval has been received from The University of Sydney Human Research Ethics Committee (2023/572). The results of the study will be published in a peer-reviewed medical journal and a plain language summary of the findings will be shared on the Wiser Healthcare publications page http://www.wiserhealthcare.org.au/category/publications/https://www.wiserhealthcare.org.au/category/publications/

**Trial registration numbers:**

Australian New Zealand Clinical Trials Registry (ID 386701 and 386889).

**Strengths and limitations of this study**

- The randomised design will provide highly relevant evidence on the potential impacts of alternative diagnostic labels for low-risk prostate cancer on decision-making of men and women with male partners.
- The study has been co-designed with consumers and clinicians to ensure evidence is relevant to end-users.
- The two large online randomised studies will be representative of men and women with male partners in the Australian community.
- The hypothetical nature of the study means it cannot capture the actual experiences of patients and their partners after an actual cancer diagnosis.
- The study does not include male partners of men with a potential prostate cancer diagnosis. This is an important group for further research.

## Introduction

 Over the past decades, there have been large increases in the diagnosis of prostate cancer with the widespread use of prostate-specific antigen (PSA) testing.^1^,^4^ Small reductions in prostate cancer mortality (eg, decrease of 19 per 100 000 men in Australia from the peak in mid 1990s^5^) suggest this has translated into some benefit, possibly due to earlier treatment of detected aggressive cancers.^5^ However the mortality reduction is many fold smaller than the incidence increase (eg, increase of 118 per 100 000 men at Australia’s peak incidence in 2009.^6^ These and other data indicate a large proportion of detected cancers are not biologically aggressive^5 7 8^ and their detection represents overdiagnosis. Overdiagnosis refers to prostate cancer diagnoses that meet pathology diagnostic criteria, but that would not have caused symptoms or shortened the person’s life if they’d been left undetected and untreated.^9^ Overdiagnosis often leads to overtreatment with the risk of adverse effects such as erectile dysfunction and urinary incontinence.^10^,^12^ As well as overdiagnosis and overtreatment, other potential harms from PSA testing include false-positive and false-negative results, psychological stress, biopsy complications including use of and harms from, prophylactic antibiotics, labelling effect,^13^ depression,^14^ financial burden^15^ and increased social inequity in the use of healthcare resources.^16^

Avoiding immediate treatment of patients with low-risk cancers, and monitoring the patient instead, has emerged as one way to mitigate potential harms.^17^ Monitoring varies in intensity, both in testing frequency, test types used and thresholds to initiate treatment with surgery or radiation therapy. While randomised controlled trials have demonstrated comparative effectiveness of observation with additional care for symptomatic disease spread (‘watch and wait’), or active surveillance using PSA monitoring with delayed intervention with surgery or radiation,^18^,^22^ many active surveillance protocols used in practice also use periodic multiparametric MRI, biomarkers and biopsies^23^ in addition to PSA to influence treatment decisions.

Active surveillance is now widely recommended as a preferred management option, but uptake varies substantially within and between countries.^24^ This may reflect differences in clinical guideline recommendations, clinician beliefs and surveillance protocols.

Patients often lack awareness of possible options and may find it difficult to understand and weigh up potential benefits and harms to decide between them.^25^ Furthermore, individuals (and their clinicians) may perceive prostate cancer as being inevitably fatal or at least causing serious morbidity if not immediately treated. Effective strategies are needed to overcome patient and clinician barriers to choosing and adhering to conservative management (observation or active surveillance) that avoids the harms of unnecessary immediate treatment (surgery or radiotherapy). One potential strategy is to use an alternative diagnostic label that does not include the word ‘cancer’.^17 23 26^ Evidence on low-risk thyroid papillary microcarcinoma and ductal carcinoma in situ of the breast suggests that avoiding the term cancer could potentially increase the uptake of conservative management and thus improve long-term health outcomes while reducing harms.^27 28^

Two previous hypothetical randomised online experiments of prostate cancer labels have been reported. In Hudnall *et al*, 748 men without a history of prostate cancer were presented with a hypothetical scenario in which they were given a diagnosis for a low-risk prostate lesion.^29^ The men were randomised to receive one of three diagnoses using different terminology to describe the same condition: Prostate cancer Gleason Score 6 (measured on a scale from 2 to 10), versus Prostate Cancer Gleason Group 1 (measured on a scale from 1 to 5), versus indolent lesion of epithelial origin (‘IDLE’). Authors found that participants in the ‘Prostate Cancer Gleason Group 1’ label had a lower preference for immediate definitive treatment and had lower ratings of anxiety after their hypothetical diagnosis compared with the ‘Gleason Score 6’ or ‘IDLE’ labels. Berlin *et al* conducted a discrete choice experiment in three participant groups: 194 healthy men without a history of prostate cancer, 159 partners and 159 men with a history of low-risk prostate cancer (grade Group 1).^30^ Using hypothetical scenarios relating to a Gleason Group 1 prostate cancer, the authors tested different possible labels to describe pathology findings (‘adenocarcinoma’ vs ‘acinar neoplasm’ vs ‘prostatic acinar neoplasm of low malignant potential’ vs ‘prostatic acinar neoplasm of uncertain malignant potential’ (‘PAN-LMP’)), and the disease in general (‘cancer’ vs ‘neoplasm’ vs ‘tumour’ vs ‘growth’). The study found that avoiding the terms cancer and adenocarcinoma increased the likelihood of preferring active surveillance by 17%. Participants showed strong preferences for ‘tumour’ over ‘cancer’ and for ‘PAN-LMP’ over ‘adenocarcinoma’. More generally, less medicalised terms for a condition tend to be associated with less invasive management choices.^31^

The proposed study seeks to expand on this work. To ensure the relevance of our findings to end-users, we will test alternative labels for low-risk prostate cancer that were chosen by our Clinician and Consumer Co-Investigators. Alternative label(s) need to be acceptable to patients and clinicians, and convey the low, but not zero, risk of disease progression.^32^ We will undertake separate studies for men and for women who have male partners, with sufficient sample sizes to estimate label effects in both populations. We are studying women with male partners as they are often involved in deciding the preferred management of low-risk prostate cancer. Also, the diagnosis can have impacts on the female partner’s mental and physical well-being, as well as on the man receiving the diagnosis.^33 34^

This study aims to explore whether using an alternative diagnostic label to communicate a hypothetical low-risk prostate cancer diagnosis influences management choice and level of anxiety among Australian men and their partners. It further seeks to understand whether the provision of absolute risk information modifies any diagnostic label effects.^35^

## Methods and analysis

### Study structure

Two separate online randomised studies of Australian residents will be run for men and for women. For both studies, participants will be randomised to receive one of three hypothetical scenarios about the diagnosis of a low-risk prostate cancer received by themselves or their partner. Participants within each label group are then randomised to high information or low information condition, referring to the level of detail presented about the possible management options. Each group will be presented with a different diagnostic label, and we will survey participants about their preferred choice of management for that diagnosis, their level of anxiety about that diagnosis and their level of anxiety about that management choice.

**Figure 1:**
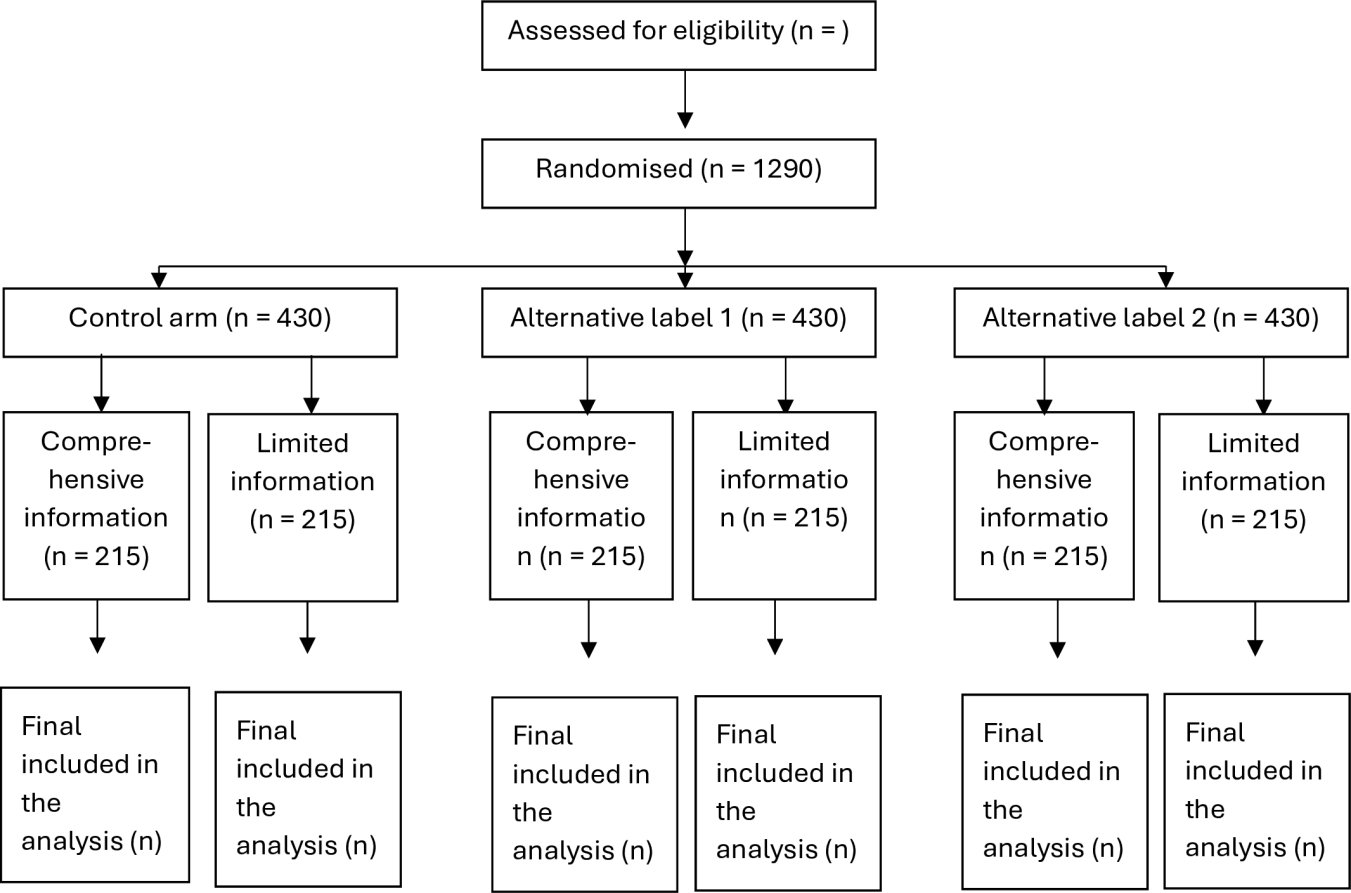
Study Consolidated Standards of Reporting Trials flow diagram for men.

The studies are structured as between-subjects factorial (3×2) online randomised experiments. The primary outcome and secondary outcomes will be compared across these randomised groups. There will be an equal probability of being assigned to each of the six groups, and we expect approximately equal numbers per group. We will use Qualtrics survey software to randomly allocate participants into groups, present the scenarios, survey questions and collect data on the outcomes (Qualtrics, Provo, Utah, USA, 2020). Our participants flow diagrams present a summary of the randomisation of participants into the allocated control and intervention arms (figures1  2).

**Figure 2:**
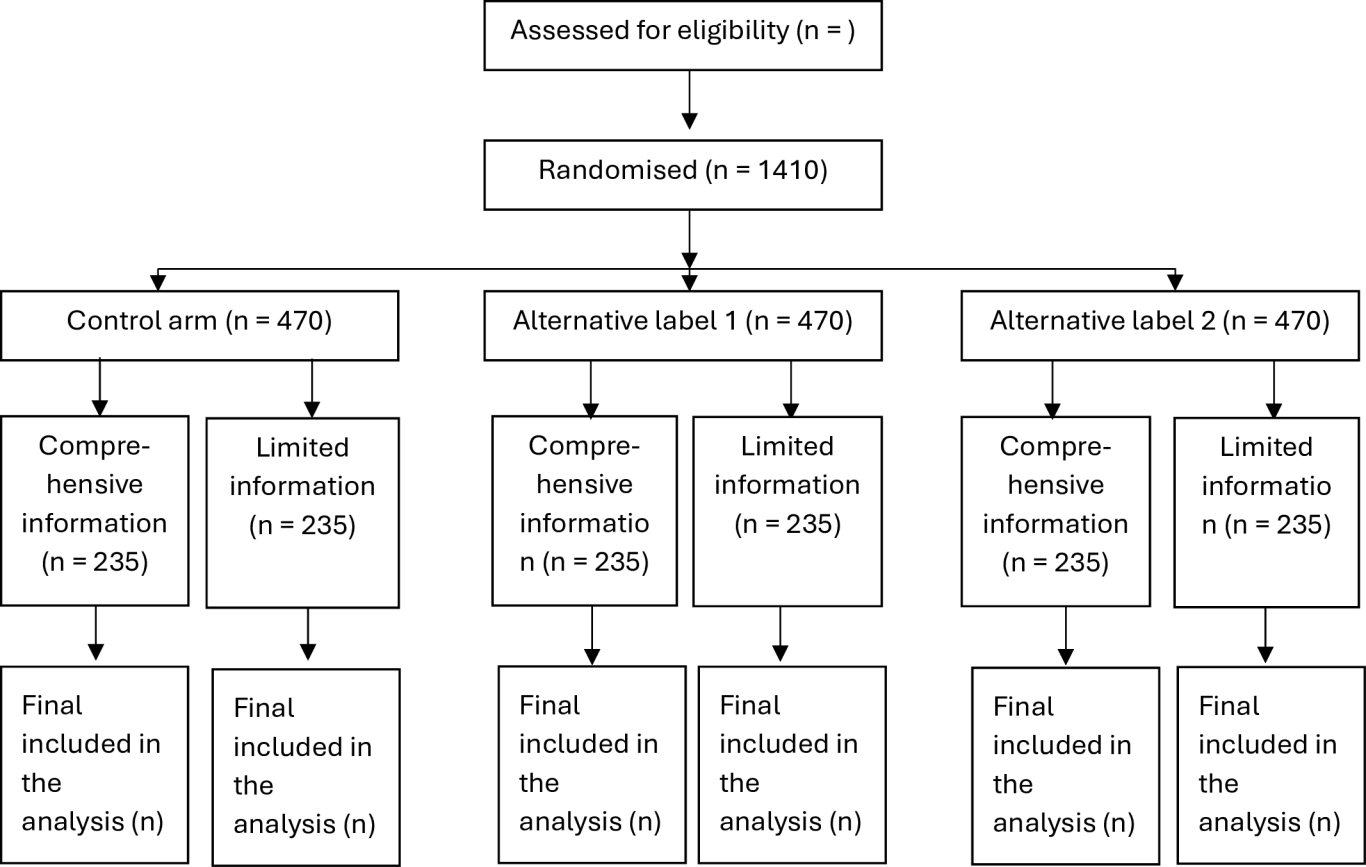
Study Consolidated Standards of Reporting Trials flow diagram for women with a male partner.

### Eligibility criteria

Two categories of participants will be eligible for the study: (1) men and (2) women. For men, participants must be 50 years or older, understand written English and be living in Australia to be included. For women, participants must have a male partner who is 50 years or older, understand written English and be living in Australia. Participants will be excluded if they, or their partner, have a history of prostate cancer.

### Recruitment and data collection

Participants will be recruited by the market research provider Qualtrics, from their pre-arranged pool of respondents who have agreed to be contacted to respond to surveys.^36^ Quotas will be used to ensure that approximately 50% of respondents are without tertiary education and the sample includes people from all Australian states and territories.

Participants who agree to participate in the study will complete an online Qualtrics survey managed by the research team. Only eligible participants will proceed to the randomisation step. The survey will capture baseline data and characteristics of participants including socio-demographic details including their age, location, health literacy and personal and family history of any cancer, personal (or partner’s) history of PSA testing and participant responses on outcome measures. The survey questions are presented in the online supplemental file 1.

All data will be collected via Qualtrics software and hosted on The University of Sydney secure server. Information will be de-identified and we will not be able to link the survey back to participants. The non-identifiable data will be downloaded for analysis and stored within The University of Sydney’s Research Data Store.

### Intervention

Participants will be randomised using Qualtrics randomisation software to receive one of three hypothetical scenarios. They will not be blinded. In each scenario, the participant will be told they have received a particular diagnosis. Group 1 (the control group) will be told they have a ‘low-risk prostate cancer, Gleason Group 1’. The alternative labels for Groups 2 and 3 were chosen by Consumer and Clinician Co-Investigators (see section v. Determination of alternative labels to be tested). Group 2 will be told they have a ‘low-risk prostate neoplasm’, and Group 3 will be told they have a ‘low-risk prostate lesion’.

**Figure 3:**
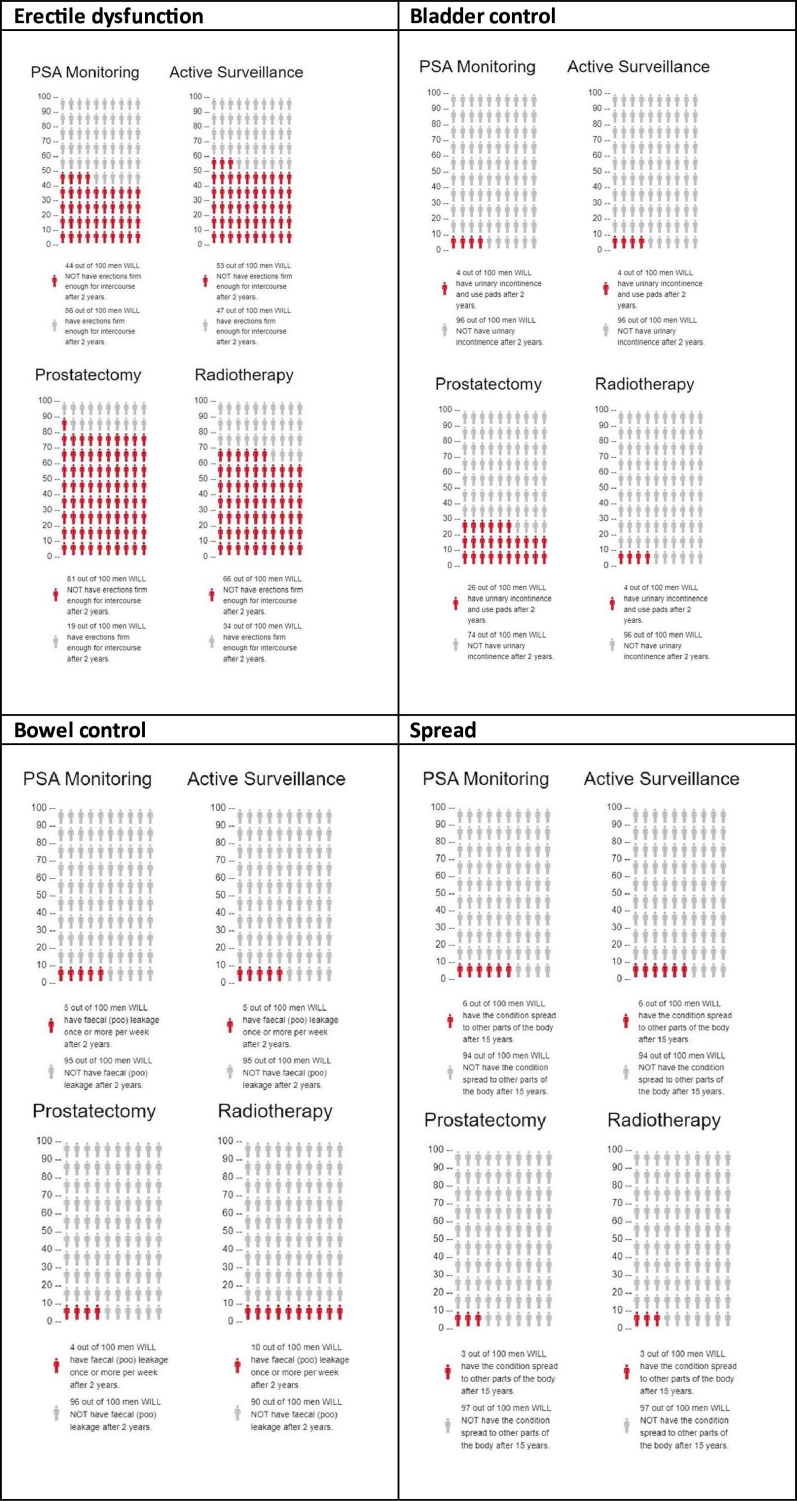
Additional information on absolute benefits and harms of management options provided in high information condition.^1^ Risk estimates are based on long-term follow-up in the PIVOT^18^,^20^ and ProtecT randomised controlled trials.^12 21 22^ PSA, prostate-specific antigen.

Within each scenario arm, participants will be further randomised to receive either limited information about their diagnosis and management options (a short description of the diagnosis and the management options available, see box 1) or more comprehensive information about their diagnosis and management options (additionally including the absolute rates of problems with erectile, urinary and bowel function and of metastatic spread, as reported in the two randomised controlled trials (RCTs) that included a majority with PSA-detected cancer: the PIVOT^18^,^20^ and ProtecT RCTs^12 21 22^ (see figure 3).

Box 1 **Explanation of management options for men (equivalent text used for women with male partners)**

1. Prostate-specific antigen (PSA) monitoring, where you keep visiting the doctor to get check-ups and tests. You will be monitored at regular points in time with PSA blood tests. This is to monitor the way the (diagnostic label) behaves. If it shows signs of growing, then treatment can be started.
2. Active surveillance, where you keep visiting the doctor to get check-ups and tests. Your will be monitored at regular points in time with PSA blood tests, MRI scans and prostate biopsies. This is to monitor the way (diagnostic label) behaves. If it shows signs of growing, then treatment can be started.
3. Prostatectomy, where you have a surgical procedure to remove the prostate. This includes the (diagnostic label).
4. Radiotherapy, where you have a non-surgical procedure on the prostate. The prostate is treated with radiation to destroy the (diagnostic label).

Studies have reported the health outcomes for men diagnosed with low-risk prostate cancer, grade Group 1 who had one of these options. The studies found that for every 100 men found to have low-risk prostate cancer, grade Group 1, 2 men will die because of their condition over the next 15 years, regardless of the treatment that is given. Different ways of managing the condition have advantages and disadvantages.

### Determination of alternative labels to be tested

We undertook a targeted literature search in May 2023 by retrieving forward and backward citations of three key papers that considered low-risk prostate lesions and cancer labelling.^21 24^ We used an automated tool ‘Spidercite’^37^ to identify records, and Covidence to screen title, abstract and full-texts (Veritas Health Innovation, Australia; https://www.covidence.org). Of 362 unique records retrieved, we screened the full text of 44 and included 9 papers describing 7 alternative labels for prostate cancer (table 1). Using short online questionnaires implemented in Qualtrics (Provo, Utah, USA: Qualtrics, 2020), we ran two rounds of survey with the eight international clinician investigators (with expertise in prostate pathology, primary care, urology and radiation oncology) and four consumer investigators (two with lived experience of low-risk prostate cancer and two without a history of prostate cancer) to determine choice of alternative labels we would test in the online survey. In the first-round surveys, clinician and consumer investigators ranked the seven labels identified in the targeted literature search and an additional label ‘low-risk prostate lesion’ in order of preference. Both clinicians and consumers highly ranked ‘low-risk prostate lesion’, and this was chosen as one of the alternative labels to be tested. Clinicians also highly ranked IDLE and PAN-LMP, and one clinician suggested a new label ‘low-risk neoplasm’. In the second-round survey, in which clinicians and consumers considered these three alternative labels, ‘low-risk prostate neoplasm’ was ranked highest by both groups and so was chosen as the second alternative label to be tested.

**Table 1:** Studies that considered alternatives to low-risk prostate cancer

Authors (year)	Study type	Participants	Labels included
Berlin *et al* (2023)^30^	Discrete choice experiment	1254	Adenocarcinoma, acinar neoplasm, prostatic acinar neoplasm of low malignant potential, prostatic acinar neoplasm of uncertain malignant potential, neoplasm, tumour, growth.
Zhou *et al* (2023)^45^	Comment	NA	Non-cancer.
Eggener *et al* (2022)^46^	Editorial	NA	Indolent lesion of epithelial origin.
Epstein (2022)^47^	Editorial	NA	Indolent lesion of epithelial origin.
Hudnall *et al* (2021)^29^	Online survey	748	Gleason 6 out of 10 prostate cancer, grade Group 1 out of 5 prostate cancer, indolent lesion of epithelial origin.
Ho *et al* (2019)^48^	Review	NA	Indolent lesion of epithelial origin.
Varma (2018)^49^	Comment	NA	Indolent lesion of low malignant potential, tumour of uncertain malignant potential.
Epstein (2018)^50^	Editorial	NA	Indolent lesion of epithelial origin.
Epstein *et al* (2016)^51^	Review	NA	Indolent lesion of epithelial origin.
Kulac *et al* (2015)^52^	Editorial	NA	Indolent lesion of epithelial origin.

### Primary and secondary outcomes

Primary and secondary outcomes are described in table 2. The primary outcome is participant’s choice of management option for the low-risk prostate lesion: no immediate treatment (PSA monitoring or active surveillance) versus immediate treatment (prostatectomy or radiation therapy). Secondary outcomes are: proportions choosing each individual management option (PSA monitoring, active surveillance, prostatectomy or radiotherapy), diagnosis anxiety (11-point single-question Visual Analogue Scale, with anchored endpoints of 0=not anxious at all and 10=extremely anxious),^38^ management choice anxiety (11-point single-question Visual Analogue Scale, with anchored endpoints of 0=not anxious at all and 10=extremely anxious),^38^ open-text explanation of management choice (free text input) and whether the participant would choose definitive treatment (prostatectomy or radiotherapy) after 5 years of conservative management (monitoring or active surveillance; for participants who chose these options initially).

**Table 2:** Participant characteristics and outcome measures

Variable	Measure
Participant characteristics	
General mood and well-being	WHO (5) Well-Being Questionnaire.^53^
Medical minimiser/maximiser	Single-Item Maximiser/Minimiser Elicitation Question.^54^
Health literacy	Single Item Literacy Screener.^55^
Cancer worry	Direct choice between specified options, one choice possible.
Self-efficacy	Generalised Self-Efficacy Scale.^56^
Primary outcomes	
Choice of management approach: no immediate treatment (PSA monitoring or active surveillance) vs immediate treatment (prostatectomy, radiotherapy)	Direct choice between two management approaches (patient choices between the four management options grouped into two).
Secondary outcomes	
Management choice between PSA monitoring, active surveillance, prostatectomy or radiotherapy	Direct choice between specified options, one choice possible from four options.
Diagnosis anxiety	Single-question Visual Analogue Scale (0–10).^38^
Management choice anxiety	Single-question Visual Analogue Scale (0–10).^38^
Open-text explanation of management choice	Free text (optional).
Participant management choice 5 years later (for those initially choosing PSA monitoring or active surveillance)	Direct choice between two management approaches: continue monitoring vs immediate treatment.

PSAprostate-specific antigen

### Sample size

Separate calculations were done for the required sample sizes for the studies of men and women with male partners. We estimated a sample size of 1290 participants with 430 participants per group in the study of men, and 1410 participants with 470 participants per group in the study of women with male partners, would each provide 80% power to detect a pairwise difference in the absolute proportion choosing conservative management as small as 10%.

The assumptions are: 70% of men with a prostate^30 39^ and 65% of women with male partners^30^ would choose conservative management in the control label condition, a 5% dropout rate, α=0.05, the normal approximation to the binomial distribution and the standard formula for comparing proportions in independent equal-sized groups.^40^

### Analysis

The two studies (men and women) will be run in parallel and analysed separately. Each study will include variables used in sampling strata: age, education and geographical location (by state/territory). All analyses will adhere to the intention-to-treat principle, with participant data analysed according to the randomly assigned study arm, regardless of adherence to the study protocol. We will present categorical data using counts and percentages, and continuous data using the minimum and maximum, mean and SD or median and quartile 1 (Q1) and quartile 3 (Q3). For each outcome, we will present the number of participant responses included in the analysis.

Statistical analyses will use a superiority framework to make pairwise comparisons across the three label groups. We will use logistic regression for binary outcomes and linear regression for continuous outcomes. We will present 95% CIs for effect estimates on all primary and secondary outcomes. As well as the effects of the alternative diagnostic labels, we will estimate the effects of the provision of absolute risk information. We will investigate effect modification of the label effects by provision of absolute risk information through testing significance of interaction terms with the label group variable. All hypothesis tests will be two-sided with an α of 5%. P values from secondary analyses will not be adjusted for multiple testing and so will be interpreted conservatively.

We will estimate unadjusted and adjusted effects using the relevant regression model, and will present these across important patient characteristics including: age, state or territory, relative socioeconomic disadvantage (postcode-based), remoteness (postcode-based), education, language spoken at home, family member with prostate cancer diagnosis and other participant characteristics in table 2. For the adjusted analyses, we will include baseline measurements of important prognostic factors for the outcome in the model, which is recommended to improve the power of the study^41^ and to obtain valid SEs when using stratification.^42^ These will include variables used in sampling strata: age, education and geographical location (by state/territory). Other prognostic factors will be measured through the baseline questionnaire, and include baseline anxiety levels, prior diagnosis of (non-prostate) cancer, diagnosis of prostate cancer in a family member or close friend. The effects of participants’ health literacy on intervention effects will also be explored as a potential confounder. Data analysis will be conducted in R V.4.3.2.^43^

### Patient and public involvement

Four consumer investigators are involved in this research. As well as providing valuable input into the choice of alternative labels, they have reviewed study materials to ensure these are optimum for the community samples we will recruit. The research question and outcome measures were informed by patient experiences of overdiagnosis and harm related to low-risk prostate cancers.^44^ Patients and the public will not be directly involved in the recruitment and conduct of the study. We aim to share a lay summary of the study findings with health consumer groups for dissemination in the wider community.

### Planned start and end dates for the study

The anticipated date of first participant enrolment was 01 April 2024 and the anticipated date of last data collection completion was 30 April 2024 (see Australian New Zealand Clinical Trials Registry entries ID 386701 and 386889).

### Ethics and dissemination

Ethics approval for this study was obtained by the University of Sydney’s Human Research Ethics Committee on 21 September 2023 (Project Number 2023/572). This study has been created as a project on the Open Science Framework (OSF) Platform (https://doi.org/10.17605/OSF.IO/UN9GB). Updates to the protocol will be uploaded to the OSF platform and identified by version number. The two trials are registered with the Australian New Zealand Clinical Trials Registry. Updates to the protocol will also be uploaded to the registry and identified by version number.

As this study is an online randomised experiment that includes a hypothetical survey, we do not anticipate significant adverse events because of the trial interventions or conduct. Participants are reminded at several points before and after the study as part of the participant information, consent and debrief processes that the nature of the study is hypothetical, that none of the information relates to their actual health or well-being and that researchers do not have access to their actual medical histories or information. The debriefing content also includes links to relevant resources for participants who wish to find out more.

The research team will have access to the final trial data set. Access may be granted to other researchers on reasonable request. No contractual agreements limit the disclosure of data to other investigators.

The findings of the study will be published in a peer-reviewed medical journal. A lay summary of the findings will be published via the permanent link at the Wiser Healthcare publications page. This research will provide robust evidence on the potential effects of proposed changes to the terminology of low-risk prostate lesions on uptake of, and persistence with, active surveillance. It may inform policy discussion, public health approaches and medical guidelines regarding which prostate lesions have a cancer label. It may also help to inform clinician practice regarding how clinicians discuss prostate cancer, its risks and management options.

## supplementary material

10.1136/bmjopen-2024-085947 online supplemental file 1
