# Supplementary material for: Impact of the diagnostic label for a low-risk prostate lesion: protocol for two online factorial randomised experiments
Source: BMJ Open. 2024 Aug 9;14(8):e085947. doi: 10.1136/bmjopen-2024-085947 (PMC11331948; doi:10.1136/bmjopen-2024-085947)
Supplement: online supplemental file 1 [file bmjopen-14-8-s001.pdf]

Landing Page ✓

Does the label given to a low-risk prostate lesion influence management choice: a randomised experiment.

Thank you for your interest in our study about low-risk prostate lesions.

In this study, you will be randomised to be shown one of three hypothetical scenarios following a routine prostate screening, which will be followed by questions about management options and anxiety.

The study is being conducted by a team of researchers and clinicians from The University of Sydney School of Public Health.

Taking part in the study involves completing one online questionnaire which will take approximately 5 to 10 minutes to complete.

Being in this study is completely voluntary and you do not have to take part. Your decision on whether to participate will not affect your current or future relationship with the researchers or anyone else at the University of Sydney.

Please take the time to read through the **Participant Information Statement** below.

If you are interested in taking part in this study, you will be asked to consent to take part by ticking the 'yes' box at the beginning of the questionnaire. By giving

your consent to take part in this study, you are telling us that you:

✓ Understand what you have read in the Participant Information Statement.
✓ Agree to take part in the research study as outlined in the Participant Information Statement.
✓ Agree to the use of your personal information as described.

When you have consented, you will fill out an **online questionnaire** that asks a series of questions:

- Demographic questions, including age, education, income level and relationship status.
- General health and cancer-related questions.
- Prostate cancer knowledge and prostate cancer history-related questions.

You will be randomised to read one of three **HYPOTHETICAL EXAMPLES** (these are made-up examples) in which different labels are used to explain a low-risk prostate lesion result. **Please note that you WILL NOT be receiving information or advice on any real prostate screening result or information about your actual health status**.

The hypothetical examples will be followed by questions asking your choice of management strategy and your personal perspective.

Pre-Survey PIS ✓

Effect of the diagnostic label for a low-risk prostate lesion on management strategy

PARTICIPANT INFORMATION STATEMENT

(1) What is the study about?

You are invited to participate in a study that assesses how different diagnostic labels for a low-risk prostate lesion affect a person's intention to undergo different treatments, and their anxiety. We are interested in a range of views and experiences.

(2) Who is running the study?

The study is being conducted by a team of researchers and clinicians. The team members are:

• Professor Katy Bell (School of Public Health at the University of Sydney).
• Dr Brooke Nickel (School of Public Health at the University of Sydney).
• Mr James Bullen (School of Public Health at the University of Sydney).
• Professor Kirsten McCaffery (School of Public Health at the University of Sydney).

Professor Katy Bell and Dr Brooke Nickel are leading the study.

(3) What will the study involve for me?

If you agree to participate, you will complete an online questionnaire asking for some background information about yourself and your medical history. Your results will not be able to be identified as yours - no identifiable data will be collected. You will be randomised to be shown one of three hypothetical scenarios about low-risk prostate lesion results, which will be followed by questions about treatment choice, anxiety and cancer concern. After completing and submitting this questionnaire, there will be no further contact anticipated between yourself and the research team.

(4) How much time will the study take?

The study involves one online questionnaire which will take approximately 5-10 minutes to complete.

(5) Who can take part in the study?

To be included in the trial, participants must:

For men:

• Be 50 years or older.
• Understand written English.
• Be living in Australia.

For women:

• Have a male partner who is 50 years or older.
• Understand written English.
• Be living in Australia.

Participants will be excluded if they or their partner:

• Has a history of prostate cancer.

(6) Do I have to be in the study? Can I withdraw from the study once I've started?

Being in this study is completely voluntary and you do not have to take part. Your decision whether to participate will not affect your current or future relationship with the researchers or anyone else at the University of Sydney. Submitting your completed questionnaire is an indication of your consent to

participate in the study. You can withdraw your responses any time before you have submitted the questionnaire without giving a reason. Once you have submitted it, we will not be able to withdraw your responses due to their anonymous nature, and therefore we will not be able to tell which one is yours.

(7) Are there any risks or costs associated with being in the study?

There are no foreseeable risks involved if you participate in this study; however some participants may feel emotional when thinking about different disease treatment options. Participants who express or experience distress during the survey are not obligated to continue and can contact the Cancer Council helpline for support on 13 11 20 or info@cancer.org.au. Please contact researchers via email katy.bell@sydney.edu.au if you require further information or support. Aside from giving up your time, we do not expect that there will be any costs associated with taking part in this study.

(8) Are there any benefits associated with being in the study?

Findings from this study will provide much needed Australian-first data on the impact of different labels for low-risk prostate cancer. This said, we cannot guarantee that you will receive any direct benefits from being in the study.

(9) What will happen to the information about me that is collected during the study?

By providing your consent, you are agreeing to us collecting the personal information that you provide in your answers to the survey for this research study. None of your answers will be able to be identified as being yours - no identifiable data will be collected. Your answers will only be used for the purposes outlined in this Participant Information Statement, unless you consent otherwise. The non-identifiable data will be stored securely, and kept strictly confidential, except as required by law. We will store the data for a period of 5

years. Study findings will be published as articles in academic journals, and presented at conferences, but you will not be individually identifiable in these publications. The research team will have access to the final trial dataset. Access may be granted to other researchers on reasonable request. Sharing research data is important for advancing knowledge and innovation. The non-identifiable data collected in this study may be made available for use in future research.

(10) Can I tell other people about the study?

Yes, you are welcome to tell other people about the study.

(11) What if I would like further information about the study?

When you have read this information, Prof Katy Bell will be available to discuss it with you further and answer any questions you may have. If you would like to know more at any stage during the study, please feel free to contact Prof Katy Bell on katy.bell@sydney.edu.au.

(12) Will I be told the results of the study?

You have a right to receive feedback about the overall results of this study. The results of the study and a plain language summary of the findings will be published at the permanent web page wiserhealthcare.org.au/category/publications after the study has been published.

(13) What if I have a complaint or any concerns about the study?

Research involving humans in Australia is reviewed by an independent group of people called a Human Research Ethics Committee (HREC). The ethical aspects of this study have been approved by the HREC of the University of

Sydney. As part of this process, we have agreed to carry out the study according to the National Statement on Ethical Conduct in Human Research (2007). This statement has been developed to protect people who agree to take part in research studies. If you are concerned about the way this study is being conducted or you wish to make a complaint to someone independent from the study, please contact the university using the details outlined below. Please quote the study title and protocol number 2019/1030.

The Manager, Ethics Administration, University of Sydney:
• Telephone: +61 2 8627 8176
• Email: human.ethics@sydney.edu.au
• Fax: +61 2 8627 8177 (Facsimile)

Pre-Survey Consent Form ✓

Do you consent to take part in this study as described in the Participant Information Sheet?

◯ Yes
◯ No

Section 1: Screening and Socio-Demographic ✓

Please note that is no back button in this survey as we are interested in your first answer to each question.

Which of the following best describes your current gender identity?

◯ Male
◯ Female
◯ Non-binary / gender fluid

- ◯ Different identity

Do you have a prostate?

- ◯ Yes
- ◯ No

Have you been previously diagnosed with prostate cancer?

- ◯ Yes
- ◯ No

Do you have a partner?

- ◯ Spouse
- ◯ De-facto partner
- ◯ Partner who does not reside with you
- ◯ No partner
- ◯ Widowed
- ◯ Divorced or separated
- ◯ [ ] Other - please list:

Does your partner have a prostate?

- ◯ Yes
- ◯ No

Has your partner been previously diagnosed with prostate cancer?

- ◯ Yes

○ No

Has a former partner been diagnosed with prostate cancer?

○ Yes
○ No

What is your age?

What is your partner's age?

Section 1.5: Screening and Socio-Demographic Part 2

Which Australian state or territory do you currently live in?

○ New South Wales
○ Victoria
○ Australian Capital Territory
○ Queensland
○ South Australia
○ Western Australia
○ Northern Territory
○ Tasmania

Where are you located? (please enter your postcode)

What is your highest level of education?

- Year 10 or below
- Year 11
- Year 12
- Certificate I/II
- Certficate III/IV
- Advanced diploma/diploma
- Bachelor's degree
- Graduate diploma/graduate certificate
- Postgraduate degree (Master's or Doctorate)
- Level not determined

What is your current employment status?

- Permanent or ongoing
- Fixed-term contract
- Casual/temporary (no paid sick leave or annual leave)
- Self-employed
- On paid leave (e.g. maternity/paternity leave)
- Unemployed
- Retired/not in the workforce (e.g. student, home duties)

What was your total household income before taxes during the past 12 months?

- Less than AUD $30,000
- Between AUD $30,000 - $49,999
- Between AUD $50,000 - $79,999
- Between AUD $80,000 - $99,999

- ◯ Between AUD $100,000 - $149,999
- ◯ Between AUD $150,000 - $199,999
- ◯ More than AUD $200,000
- ◯ Prefer not to say

Do you have children?

- ◯ Yes
- ◯ No
- ◯ Prefer not to say

Are you of Aboriginal or Torres Strait Islander origin?

- ◯ Aboriginal
- ◯ Torres Strait Islander
- ◯ Both Aboriginal and Torres Strait Islander
- ◯ Neither Aboriginal or Torres Strait Islander
- ◯ Prefer not to say

Were you born in Australia?

- ◯ Yes
- ◯ No

What is your country of birth?

- ◯ UK
- ◯ India
- ◯ China
- ◯ New Zealand

- ◯ The Philippines
- ◯ [ ] Other

In which year did you move to Australia?

[ ]

What language do you mostly speak at home?

- ◯ English
- ◯ Mandarin
- ◯ Arabic
- ◯ Cantonese
- ◯ Vietnamese
- ◯ [ ] Other - please list:

Do you have private health insurance?

- ◯ Yes
- ◯ No
- ◯ Don't know

Section 2: General Health ✓

In general, would you say your health is ...

- ◯ Excellent
- ◯ Very good
- ◯ Good
- ◯ Fair

- Poor

Have you ever been diagnosed with cancer?

- Yes
- No
- Don't know

Which type of cancer?

- Skin
- Melanoma
- Bowel
- Lung
- Lymphoma
- Prostate
- Kidney
- ______ Other - please list:
- Don't know

Which type of cancer?

- Breast
- Bowel
- Skin
- Lung
- Uterine
- ______ Other - please list:
- Don't know

Has anyone in your immediate family (parents, siblings or children) ever been diagnosed with cancer?

- ◯ Yes
- ◯ No
- ◯ Don't know

Which type of cancer? Please tick all that apply

- ☐ Prostate
- ☐ Breast
- ☐ Skin
- ☐ Bowel
- ☐ Lung
- ☐ ________ Other - please list:
- ☐ Don't know

Who was this? Please tick all that apply

- ◯ Father
- ◯ Brother
- ◯ Son
- ◯ ________ Other - please list:

How worried are you about developing prostate cancer?

- ◯ Not worried at all
- ◯ A bit worried
- ◯ Quite worried
- ◯ Very worried

Have you ever had a PSA blood test to check for prostate cancer?

- ◯ Yes
- ◯ No
- ◯ Not sure

How worried are you about your partner developing prostate cancer?

- ◯ Not worried at all
- ◯ A bit worried
- ◯ Quite worried
- ◯ Very worried

Has your partner ever had a PSA blood test to check for prostate cancer?

- ◯ Yes
- ◯ No
- ◯ Not sure

Sometimes, medical action is clearly necessary and sometimes it is clearly not necessary. Other times, reasonable people differ in their beliefs about whether medical action is needed.

In situations where it's not clear, do you tend to lean towards taking action or do you prefer to wait and see if action is needed?

Importantly, there is no right way to be.

I strongly lean towards wait	I lean towards wait and see.	I somewhat lean towards	I somewhat lean towards	I lean towards taking action.	I strongly lean towards taking

and see.		wait and see.	taking action.		action.
○	○	○	○	○	○

The following questions are related to how you have been feeling over the past two weeks. Please read each statement and then choose the most appropriate option regarding how you felt in the last two weeks.

	At no time	Some of the time	Less than half of the time	More than half of the time	Most of the time	All of the time
I have felt cheerful and in good spirits.	○	○	○	○	○	○
I have felt calm and relaxed.	○	○	○	○	○	○
I have felt active and vigorous.	○	○	○	○	○	○
I woke up feeling fresh and rested.	○	○	○	○	○	○
My daily life has been filled with things that interest me.	○	○	○	○	○	○

Please respond to the following statements.

	Not at all true	Hardly true	Moderately true	Exactly true
I can always manage to solve difficult problems if I try hard enough.	○	○	○	○
If someone opposes me, I can find the means and ways to get what I want.	○	○	○	○
It is easy for me to stick to my aims and	○	○	○	○

	Not at all true	Hardly true	Moderately true	Exactly true
accomplish my goals.				
I am confident that I could deal efficiently with unexpected events.	◯	◯	◯	◯
Thanks to my resourcefulness, I know how to handle unforeseen situations.	◯	◯	◯	◯
I can solve most problems if I invest the necessary effort.	◯	◯	◯	◯
I can remain calm when facing difficulties because I can rely on my coping abilities.	◯	◯	◯	◯
When I am confronted with a problem, I can usually find several solutions.	◯	◯	◯	◯
If I am in trouble, I can usually think of a solution.	◯	◯	◯	◯
I can usually handle whatever comes my way.	◯	◯	◯	◯

Section 3: Health Literacy ✓

How often do you need to have someone help you when you read instructions, pamphlets or other written material from your doctor or pharmacy?

◯ Always

- Often
- Sometimes
- Occasionally
- Never

Hypothetical - Control + Low Information - Partners

Please read the information below and answer the questions that follow. You are asked to imagine as if the following scenario is true. Please answer how you would feel or react if you were in this situation, to the best of your ability.

You are at the doctor (GP) with your partner who recently had a prostate biopsy (sample of their prostate). This was because of a raised prostate specific antigen (PSA) result on a blood test.

The doctor has the biopsy test results and says:
"We found a small focus of **low-risk prostate cancer, grade group 1**."

After giving your partner these test results, the doctor explains that there are four options. These are:

Option 1. PSA monitoring: where your partner keeps visiting the doctor to get check-ups and tests. Your partner will be monitored at regular points in time with PSA blood tests. This is to monitor the way the low-risk prostate cancer (grade group 1) behaves. If it shows signs of growing, then treatment can be started.

Option 2. Active surveillance: where your partner keeps visiting the doctor to get check-ups and tests. Your partner will be monitored at regular points in time with PSA blood tests, MRI scans, and prostate biopsies. This is to monitor the way the low-risk prostate cancer (grade group 1) behaves. If it shows signs of

growing, then treatment can be started.

Option 3. Prostatectomy: where your partner has a surgical procedure to remove the prostate. This includes the low-risk prostate cancer (grade group 1).

Option 4. Radiotherapy: where your partner has a non-surgical procedure on the prostate. The prostate is treated with radiation to destroy the low-risk prostate cancer (grade group 1).

Studies have reported:

- For every 100 men found to have a low-risk prostate cancer (grade group 1), 2 will die from this over the next 15 years, regardless of the treatment.

Different treatments have different advantages and disadvantages.

Hypothetical - Control + High Information - Partners

Please read the information below and answer the questions that follow. You are asked to imagine as if the following scenario is true. Please answer how you would feel or react if you were in this situation, to the best of your ability.

You are at the doctor (GP) with your partner who recently had a prostate biopsy (sample of their prostate). This was because of a raised prostate specific antigen (PSA) result on a blood test.

The doctor has the biopsy test results and says:
"We found a small focus of **low-risk prostate cancer, grade group 1**."

After giving your partner these test results, the doctor explains that there are four options. These are:

Option 1. PSA monitoring: where your partner keeps visiting the doctor to get check-ups and tests. Your partner will be monitored at regular points in time with PSA blood tests. This is to monitor the way the low-risk prostate cancer (grade group 1) behaves. If it shows signs of growing, then treatment can be started.

Option 2. Active surveillance: where your partner keeps visiting the doctor to get check-ups and tests. Your partner will be monitored at regular points in time with PSA blood tests, MRI scans, and prostate biopsies. This is to monitor the way the low-risk prostate cancer (grade group 1) behaves. If it shows signs of growing, then treatment can be started.

Option 3. Radical prostatectomy: where your partner has a surgical procedure to remove the prostate. This includes the low-risk prostate cancer (grade group 1).

Option 4. Radiotherapy: where your partner has a non-surgical procedure on the prostate. The prostate is treated with radiation to destroy the low-risk prostate cancer (grade group 1).

Studies have reported:

- For every 100 men found to have a low-risk prostate cancer (grade group 1), 2 will die from this over the next 15 years, regardless of the treatment.

Different treatments have different advantages and disadvantages.
Please consider the diagrams on the next page for details:

Erectile dysfunction
(difficulty getting and keeping an erection)

PSA Monitoring

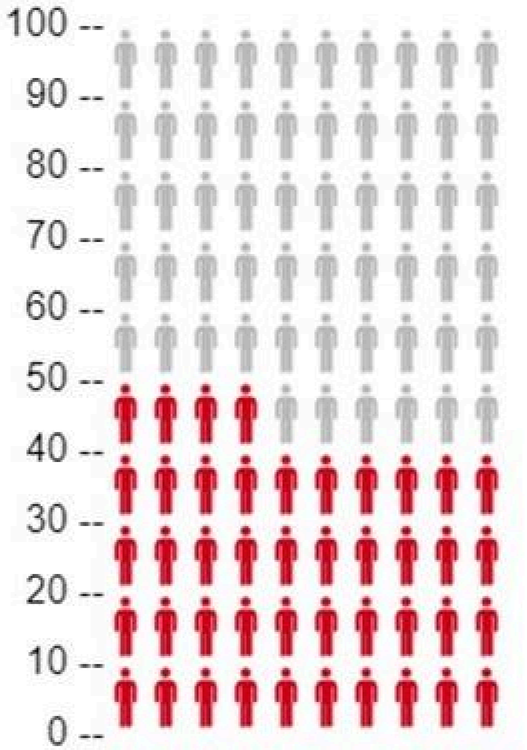

44 out of 100 men WILL NOT have erections firm enough for intercourse after 2 years.

56 out of 100 men WILL have erections firm enough for intercourse after 2 years.

Active Surveillance

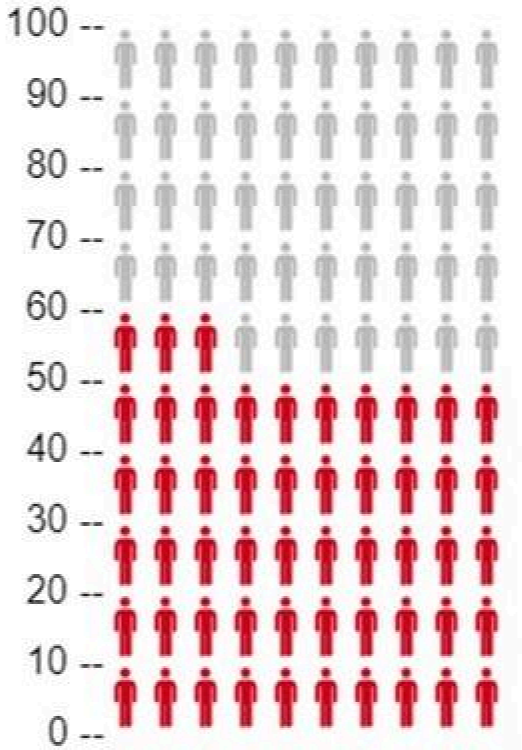

53 out of 100 men WILL NOT have erections firm enough for intercourse after 2 years.

47 out of 100 men WILL have erections firm enough for intercourse after 2 years.

Prostatectomy

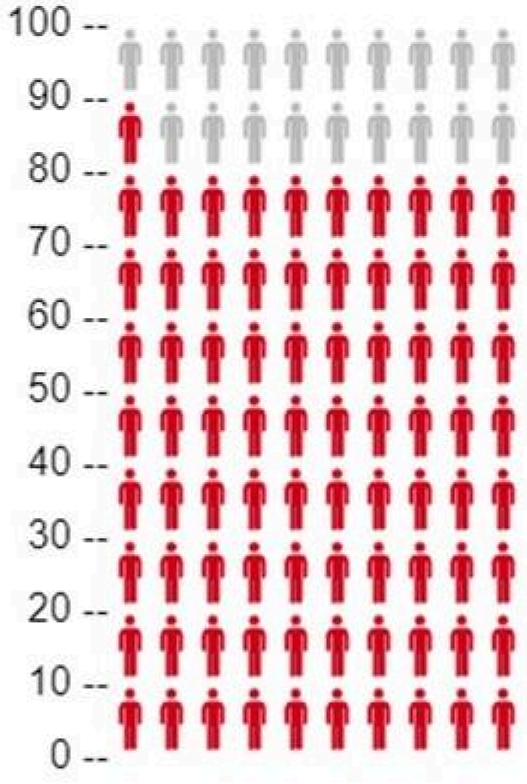

81 out of 100 men WILL NOT have erections firm enough for intercourse after 2 years.

19 out of 100 men WILL have erections firm enough for intercourse after 2 years.

Radiotherapy

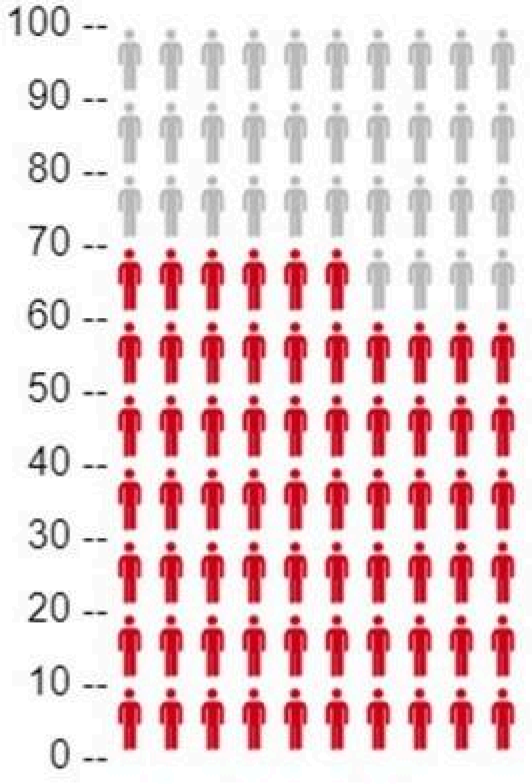

66 out of 100 men WILL NOT have erections firm enough for intercourse after 2 years.

34 out of 100 men WILL have erections firm enough for intercourse after 2 years.

Bladder Control

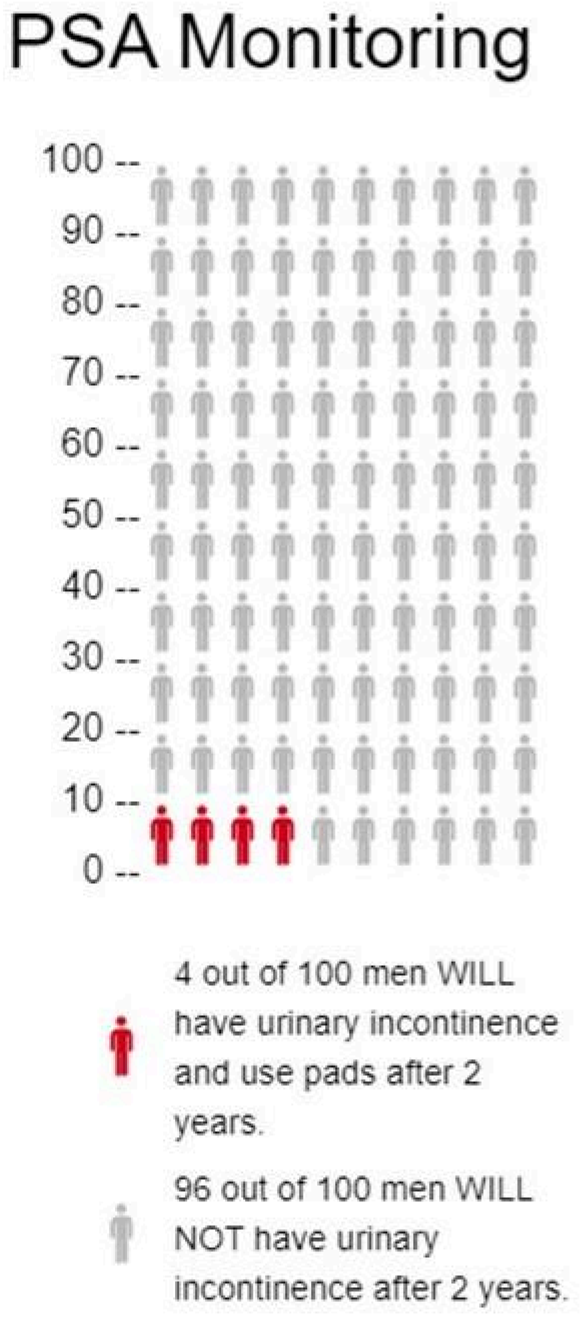

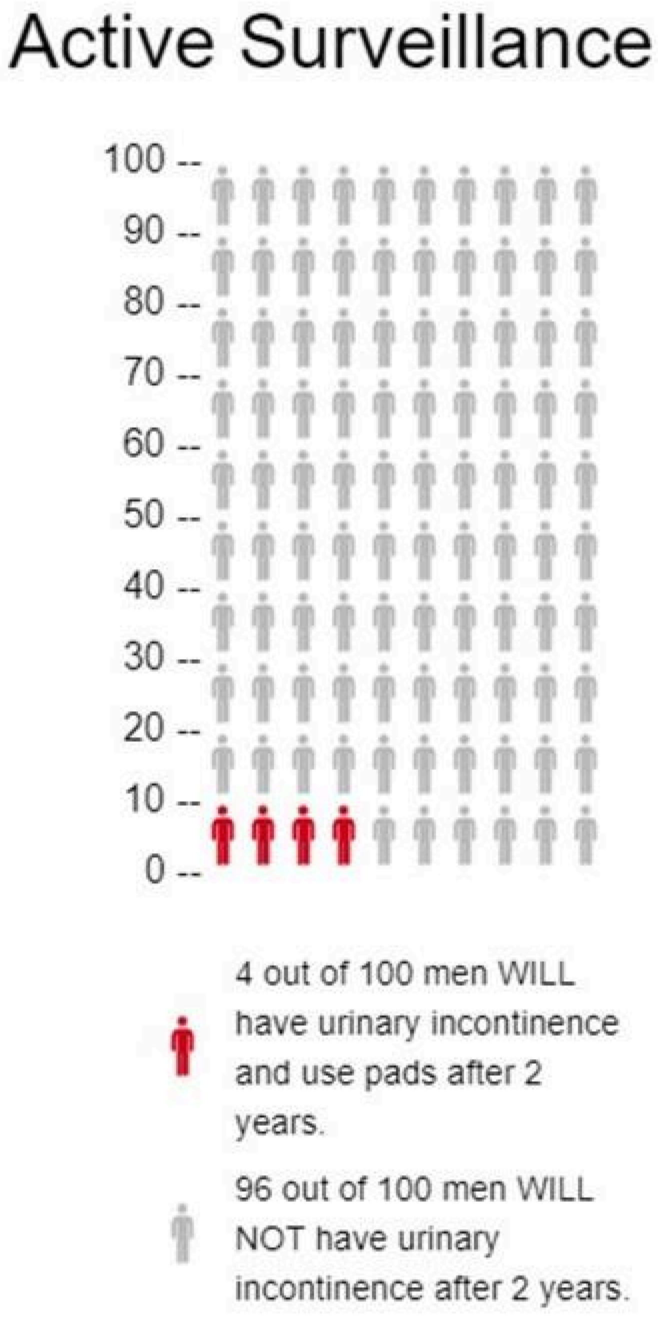

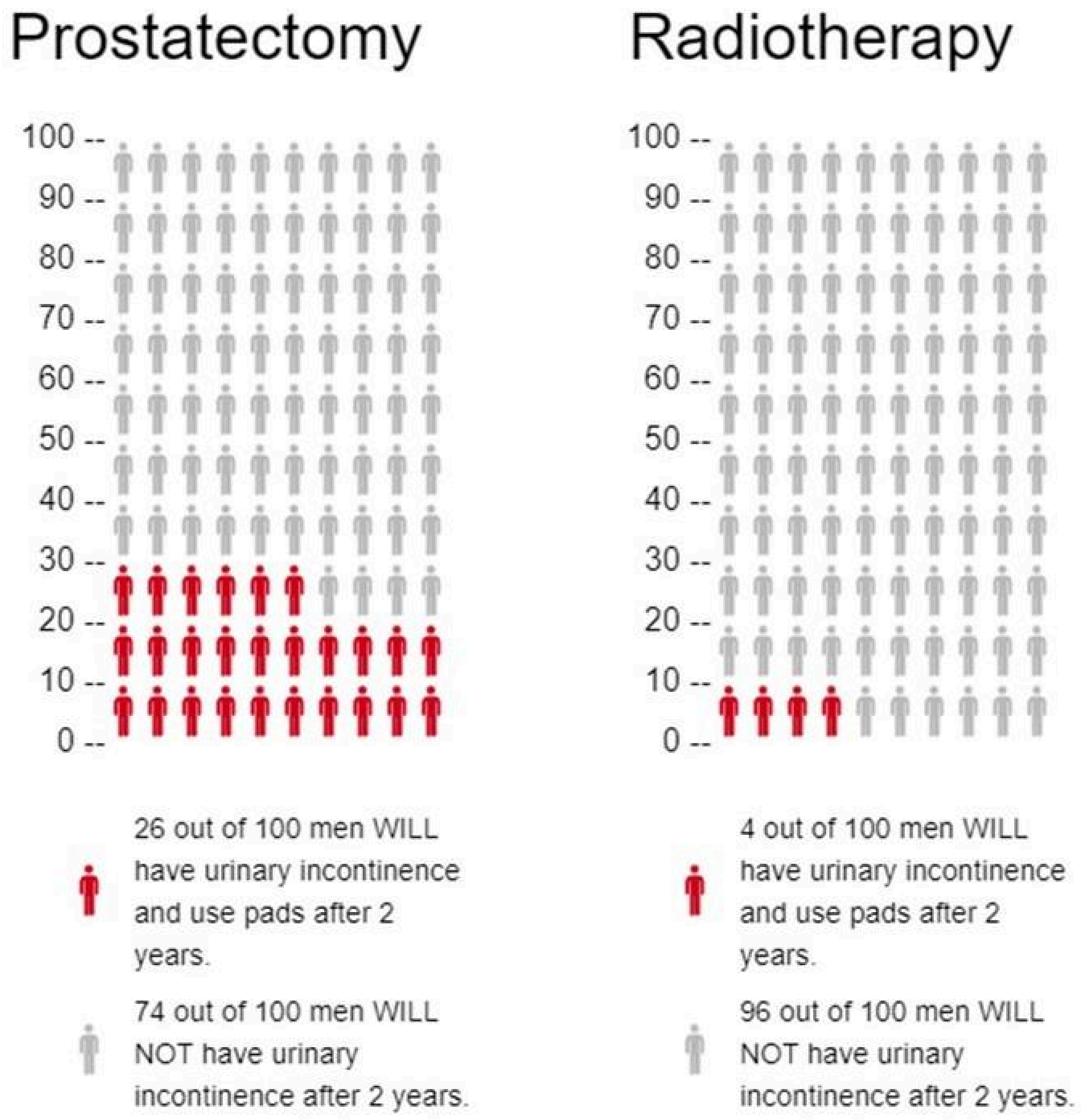
Prostatectomy
100 --
90 --
80 --
70 --
60 --
50 --
40 --
30 --
20 --
10 --
0 --
26 out of 100 men WILL have urinary incontinence and use pads after 2 years.
74 out of 100 men WILL NOT have urinary incontinence after 2 years.
Radiotherapy
100 --
90 --
80 --
70 --
60 --
50 --
40 --
30 --
20 --
10 --
0 --
4 out of 100 men WILL have urinary incontinence and use pads after 2 years.
96 out of 100 men WILL NOT have urinary incontinence after 2 years.

Bowel control

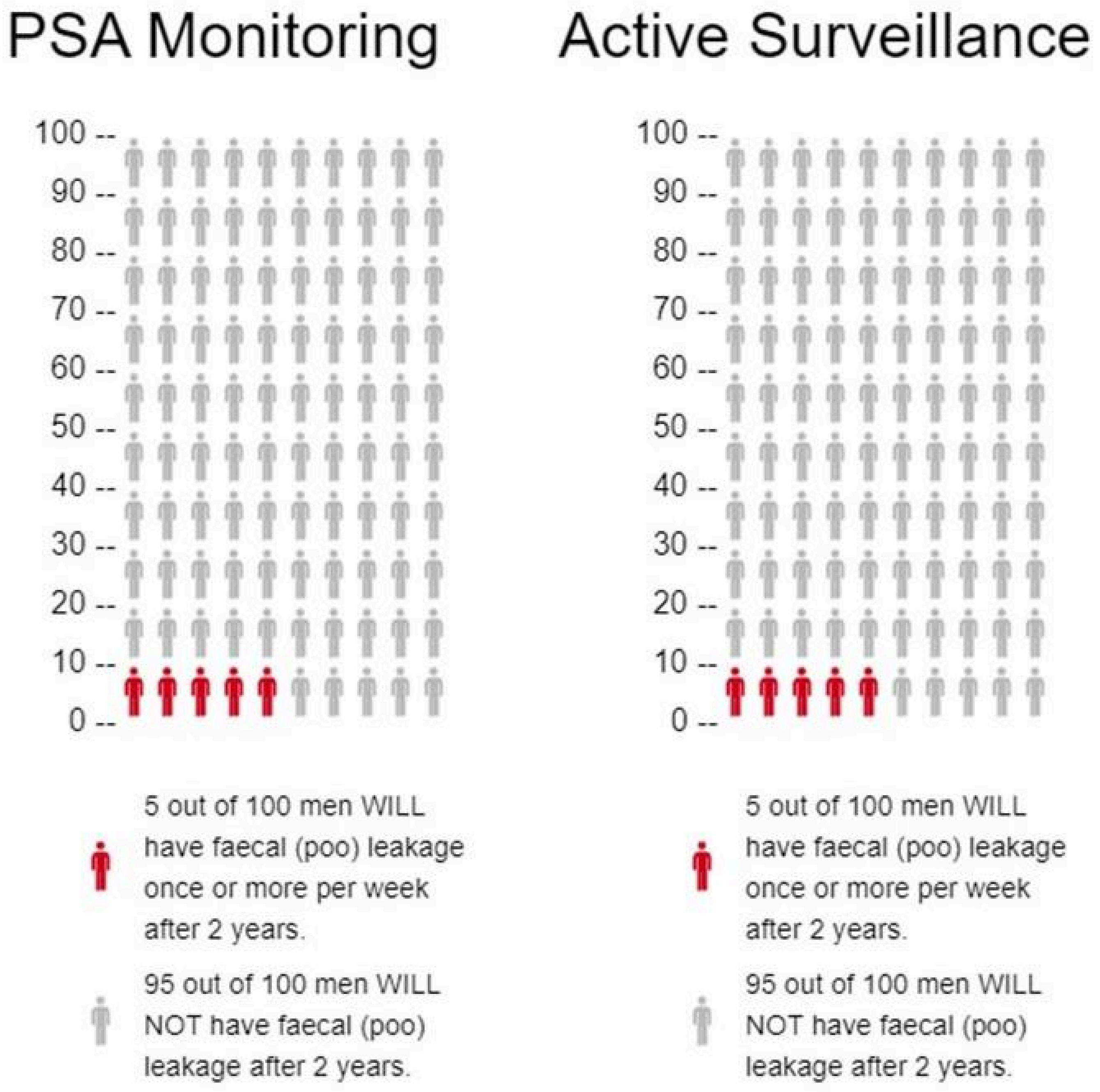

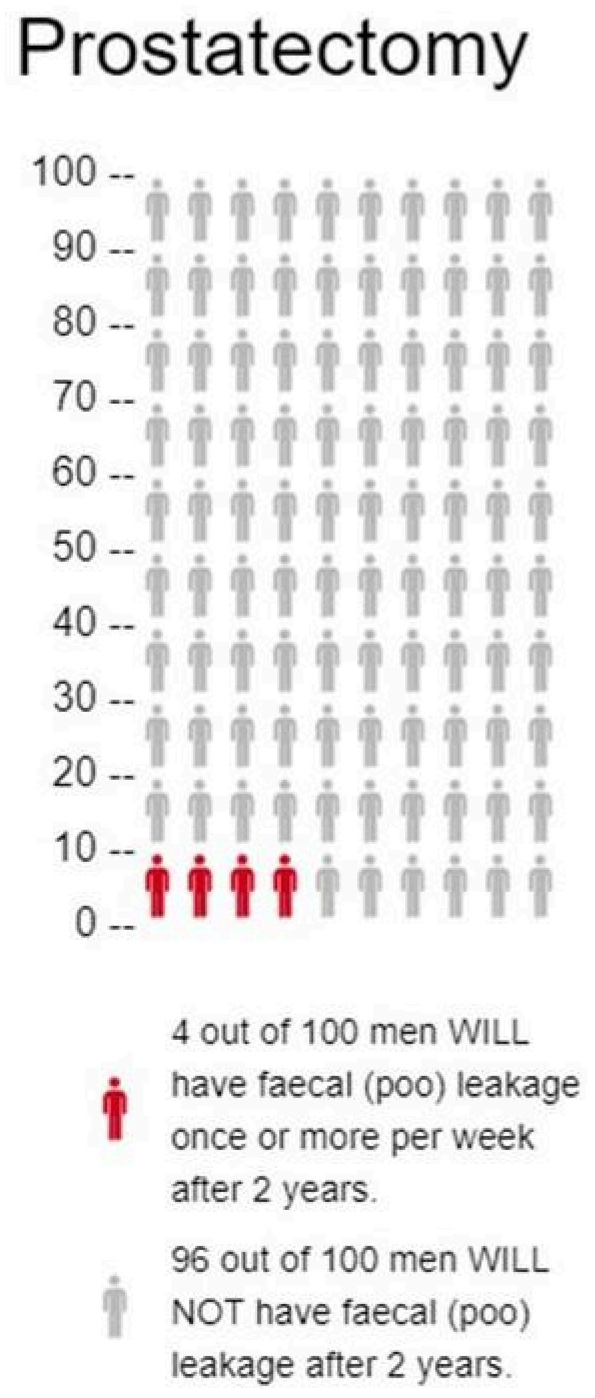
Prostatectomy
100 --
90 --
80 --
70 --
60 --
50 --
40 --
30 --
20 --
10 --
0 --
4 out of 100 men WILL have faecal (poo) leakage once or more per week after 2 years.
96 out of 100 men WILL NOT have faecal (poo) leakage after 2 years.

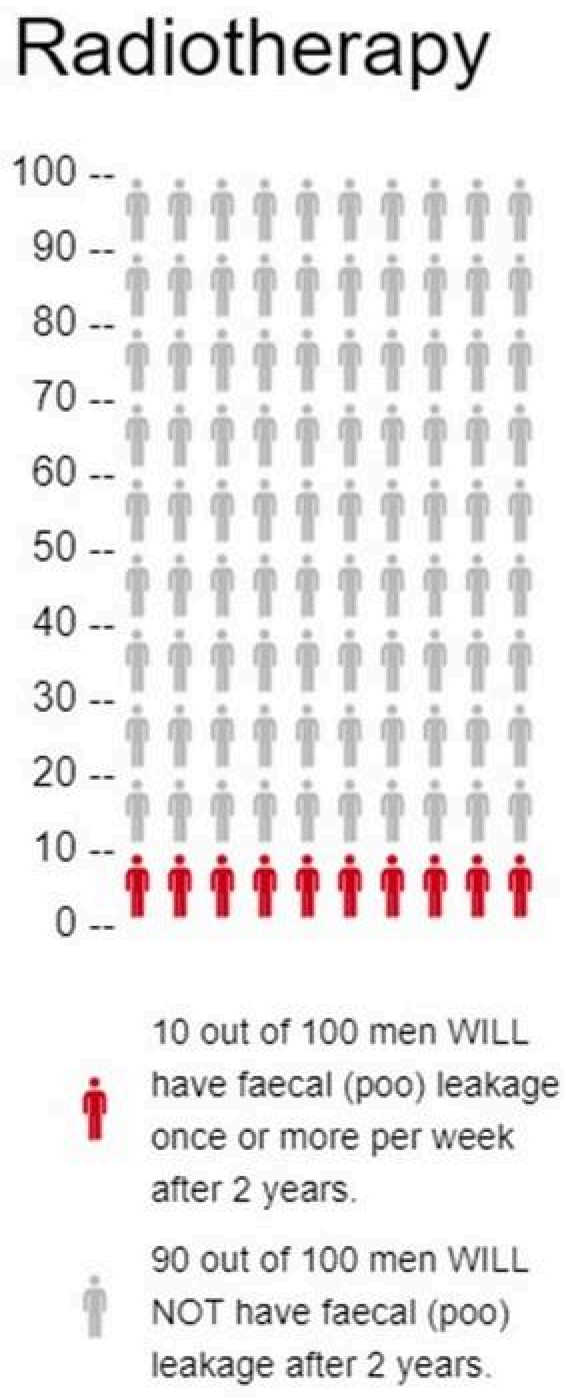
Radiotherapy
100 --
90 --
80 --
70 --
60 --
50 --
40 --
30 --
20 --
10 --
0 --
10 out of 100 men WILL have faecal (poo) leakage once or more per week after 2 years.
90 out of 100 men WILL NOT have faecal (poo) leakage after 2 years.

Spread

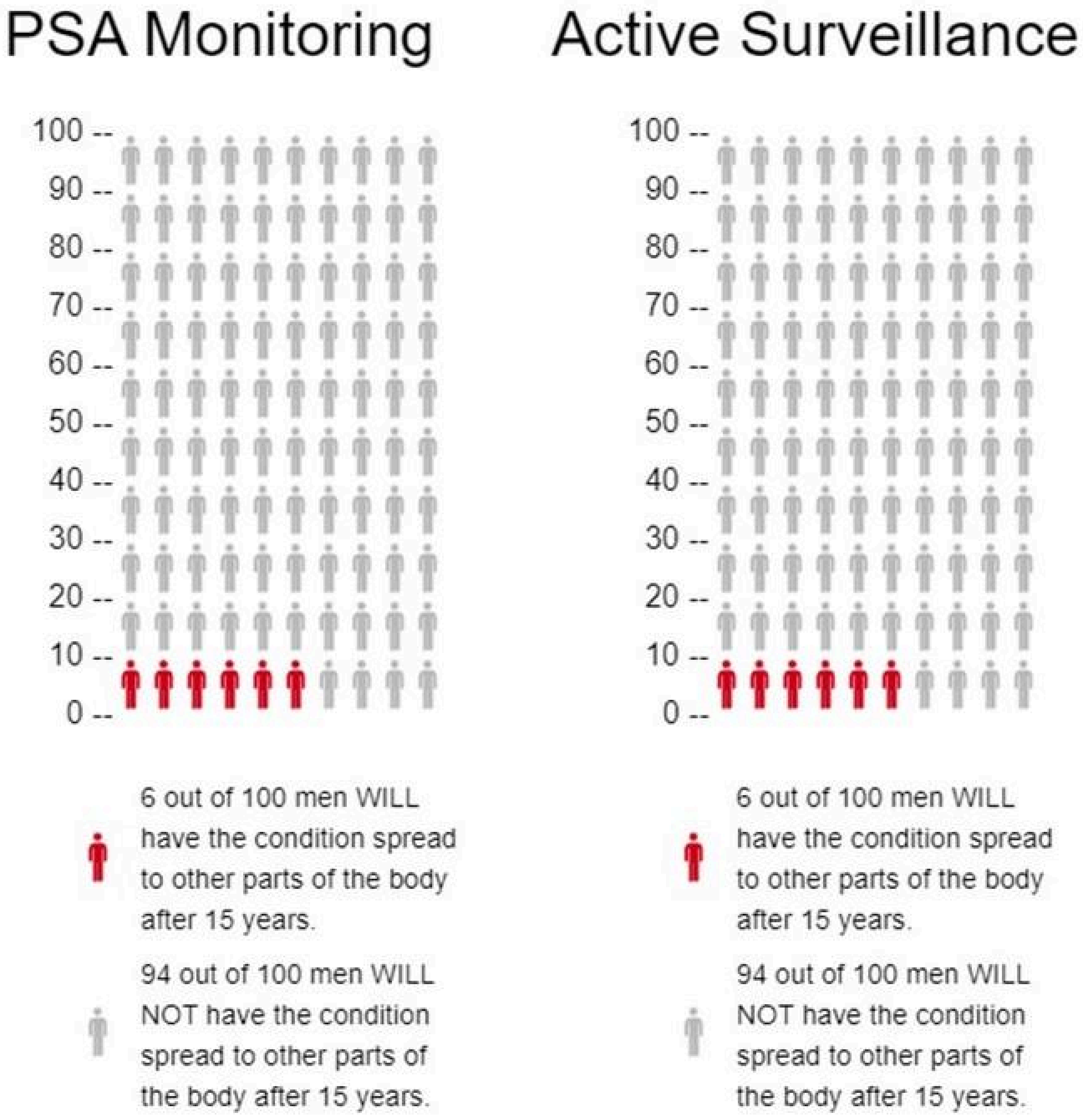
PSA Monitoring
Active Surveillance
100 --
90 --
80 --
70 --
60 --
50 --
40 --
30 --
20 --
10 --
0 --
6 out of 100 men WILL have the condition spread to other parts of the body after 15 years.
94 out of 100 men WILL NOT have the condition spread to other parts of the body after 15 years.
6 out of 100 men WILL have the condition spread to other parts of the body after 15 years.
94 out of 100 men WILL NOT have the condition spread to other parts of the body after 15 years.

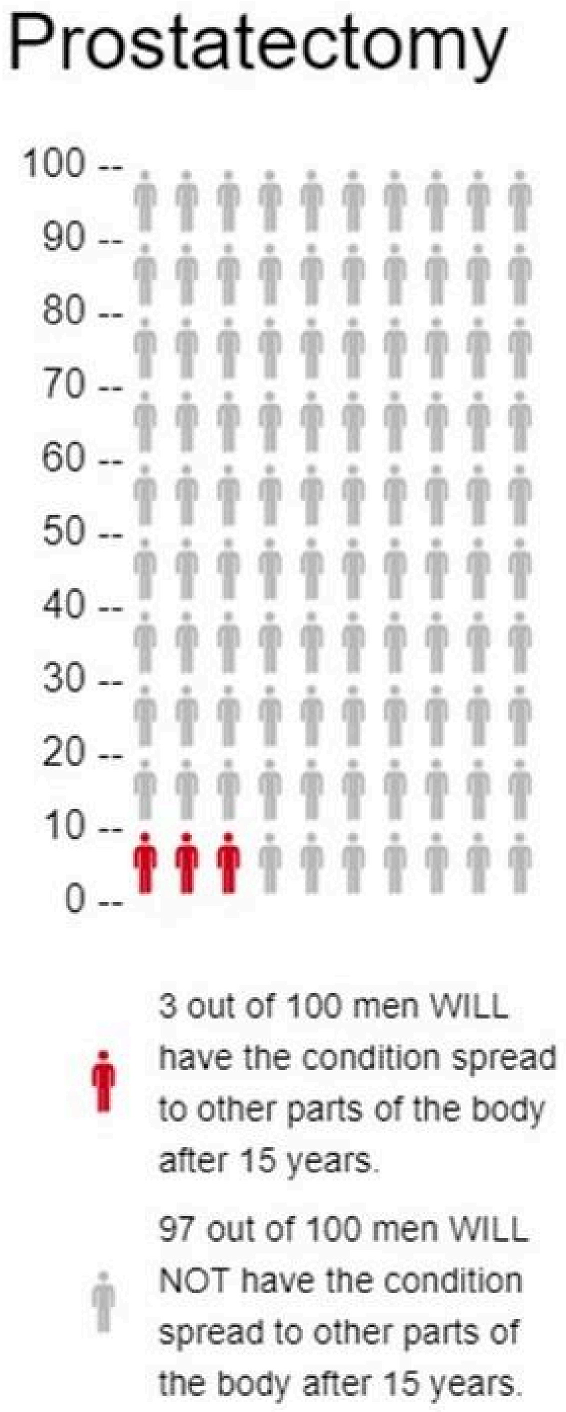

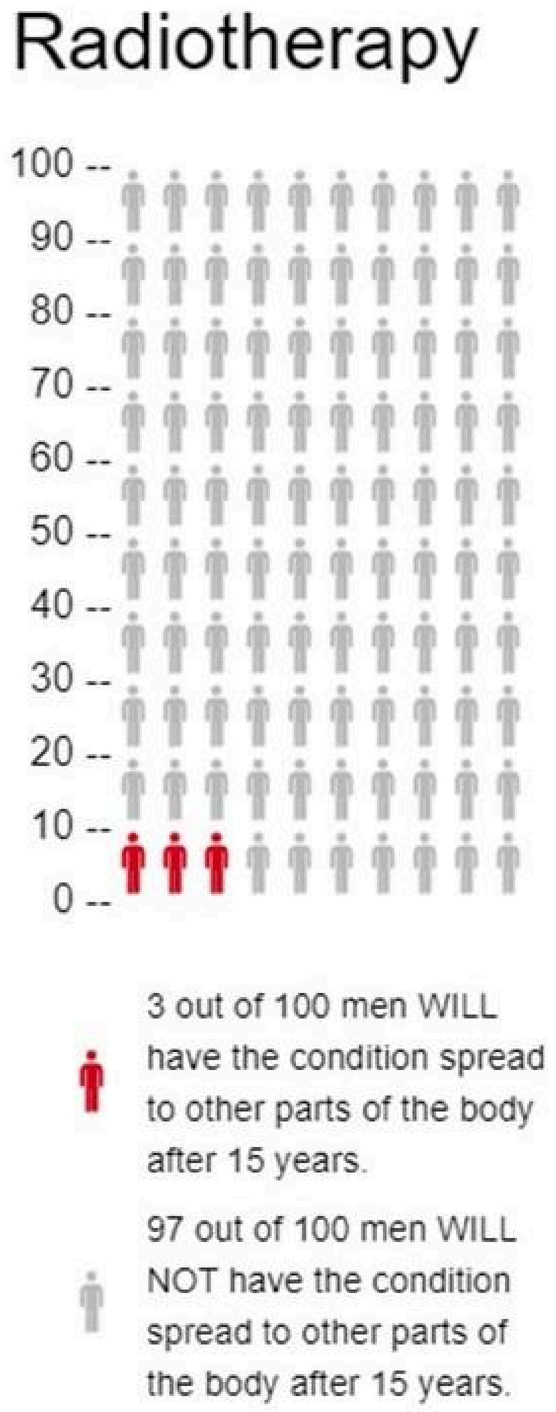

Hypothetical - Label 1 + Low Information - Partners

Please read the information below and answer the questions that follow. You are asked to imagine as if the following scenario is true. Please answer how you would feel or react if you were in this situation, to the best of your ability.

You are at the doctor (GP) with your partner who recently had a prostate biopsy (sample of their prostate). This was because of a raised prostate specific antigen (PSA) result on a blood test.

The doctor has the biopsy test results and says:
“We found a small focus of **low-risk prostate neoplasm**.”

After giving your partner these test results, the doctor explains that there are four options. These are:

Option 1. PSA monitoring: where your partner keeps visiting the doctor to get check-ups and tests. Your partner will be monitored at regular points in time with PSA blood tests. This is to monitor the way the low-risk prostate neoplasm behaves. If it shows signs of growing, then treatment can be started.

Option 2. Active surveillance: where your partner keeps visiting the doctor to get check-ups and tests. Your partner will be monitored at regular points in time with PSA blood tests, MRI scans, and prostate biopsies. This is to monitor the way the low-risk prostate neoplasm behaves. If it shows signs of growing, then treatment can be started.

Option 3. Radical prostatectomy: where your partner has a surgical procedure to remove the prostate. This includes the low-risk prostate neoplasm.

Option 4. Radiotherapy: where your partner has a non-surgical procedure on the prostate. The prostate is treated with radiation to destroy the low-risk prostate neoplasm.

Studies have reported:

- For every 100 men found to have a low-risk prostate neoplasm, 2 will die from this over the next 15 years, regardless of the treatment.

Different treatments have different advantages and disadvantages.

Hypothetical - Label 1 + High Information - Partners

Please read the information below and answer the questions that follow. You are asked to imagine as if the following scenario is true. Please answer how you would feel or react if you were in this situation, to the best of your ability.

You are at the doctor (GP) with your partner who recently had a prostate biopsy (sample of their prostate). This was because of a raised prostate specific antigen (PSA) result on a blood test.

The doctor has the biopsy test results and says:
“We found a small focus of **low-risk prostate neoplasm**.”

After giving your partner these test results, the doctor explains that there are four options. These are:

Option 1. PSA monitoring: where your partner keeps visiting the doctor to get check-ups and tests. Your partner will be monitored at regular points in time with PSA blood tests. This is to monitor the way the low-risk prostate neoplasm behaves. If it shows signs of growing, then treatment can be started.

Option 2. Active surveillance: where your partner keeps visiting the doctor to get check-ups and tests. Your partner will be monitored at regular points in time with PSA blood tests, MRI scans, and prostate biopsies. This is to monitor the way the low-risk prostate neoplasm behaves. If it shows signs of growing, then treatment can be started.

Option 3. Radical prostatectomy: where your partner has a surgical procedure to remove the prostate. This includes the low-risk prostate neoplasm.

Option 4. Radiotherapy: where your partner has a non-surgical procedure on the prostate. The prostate is treated with radiation to destroy the low-risk prostate neoplasm.

Studies have reported:

- For every 100 men found to have a low-risk prostate neoplasm, 2 will die from this over the next 15 years, regardless of the treatment.

Different treatments have different advantages and disadvantages.
Please consider the diagrams on the next pages for details:

Erectile dysfunction
(difficulty getting and keeping an erection)

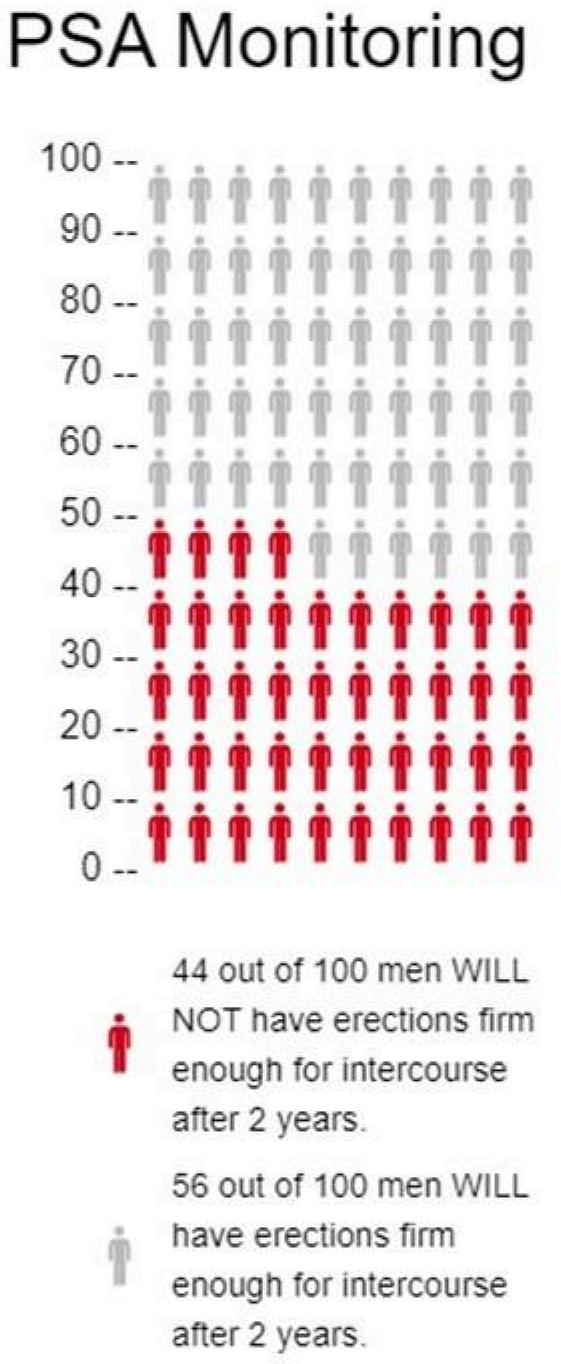
PSA Monitoring
100 --
90 --
80 --
70 --
60 --
50 --
40 --
30 --
20 --
10 --
0 --
44 out of 100 men WILL NOT have erections firm enough for intercourse after 2 years.
56 out of 100 men WILL have erections firm enough for intercourse after 2 years.

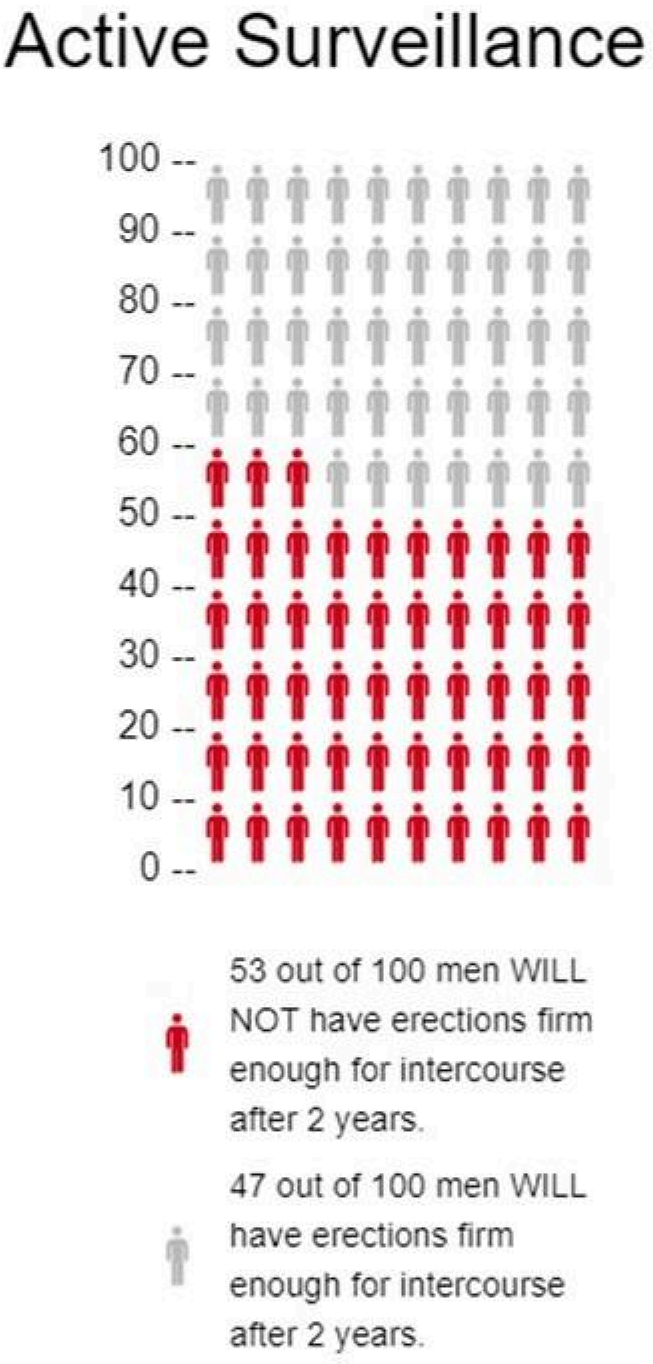
Active Surveillance
100 --
90 --
80 --
70 --
60 --
50 --
40 --
30 --
20 --
10 --
0 --
53 out of 100 men WILL NOT have erections firm enough for intercourse after 2 years.
47 out of 100 men WILL have erections firm enough for intercourse after 2 years.

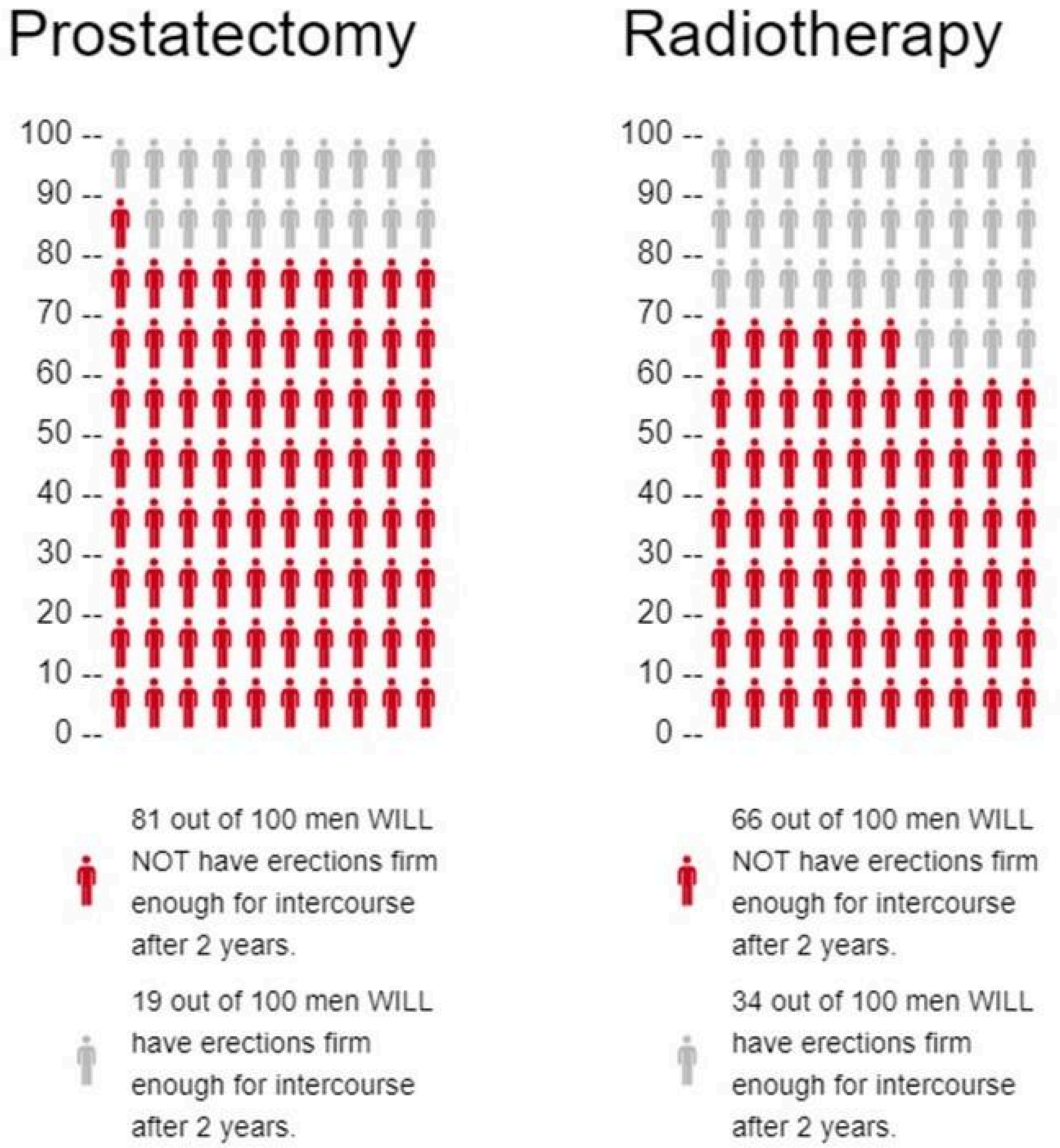
Prostatectomy
100 --
90 --
80 --
70 --
60 --
50 --
40 --
30 --
20 --
10 --
0 --
81 out of 100 men WILL NOT have erections firm enough for intercourse after 2 years.
19 out of 100 men WILL have erections firm enough for intercourse after 2 years.
Radiotherapy
100 --
90 --
80 --
70 --
60 --
50 --
40 --
30 --
20 --
10 --
0 --
66 out of 100 men WILL NOT have erections firm enough for intercourse after 2 years.
34 out of 100 men WILL have erections firm enough for intercourse after 2 years.

Bladder control

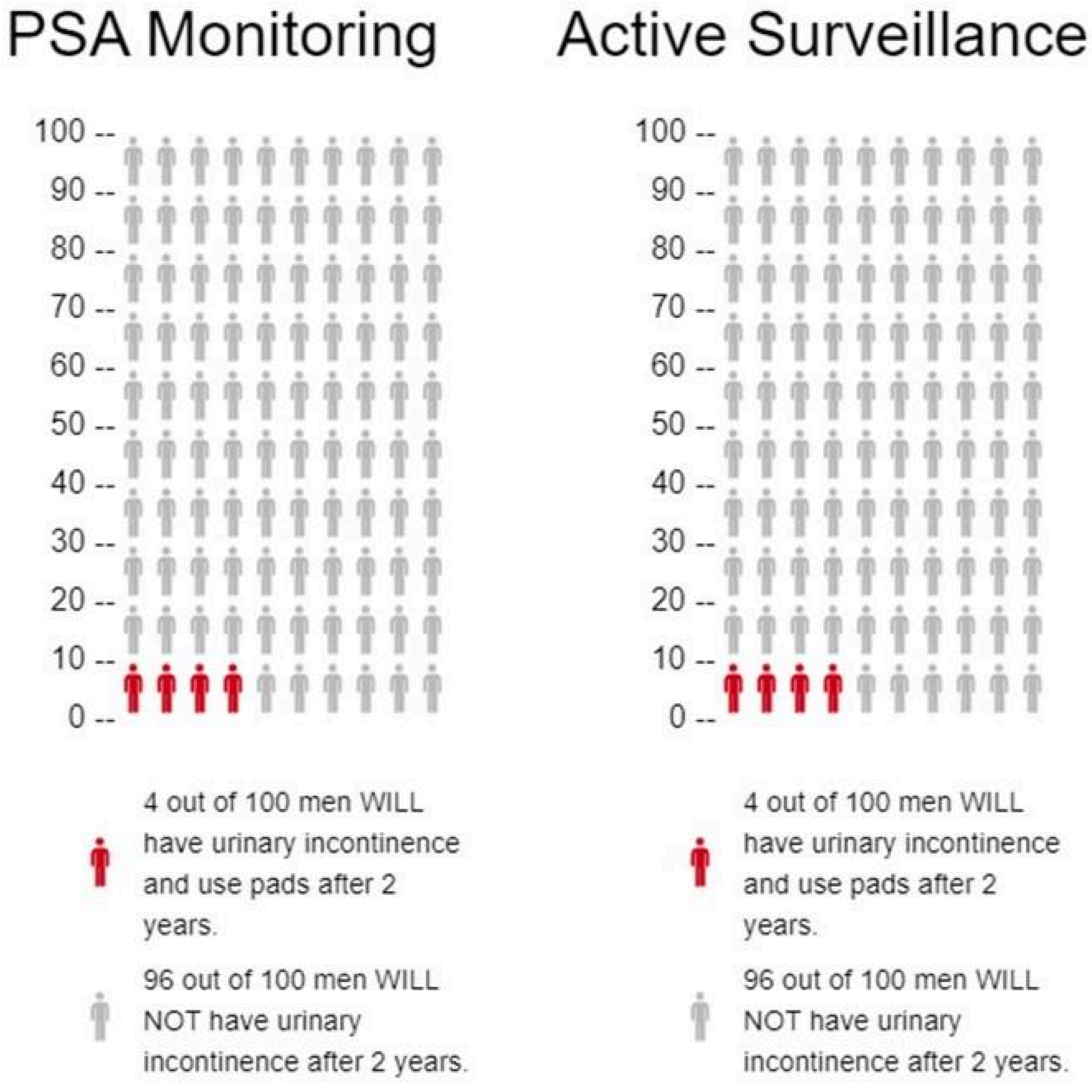

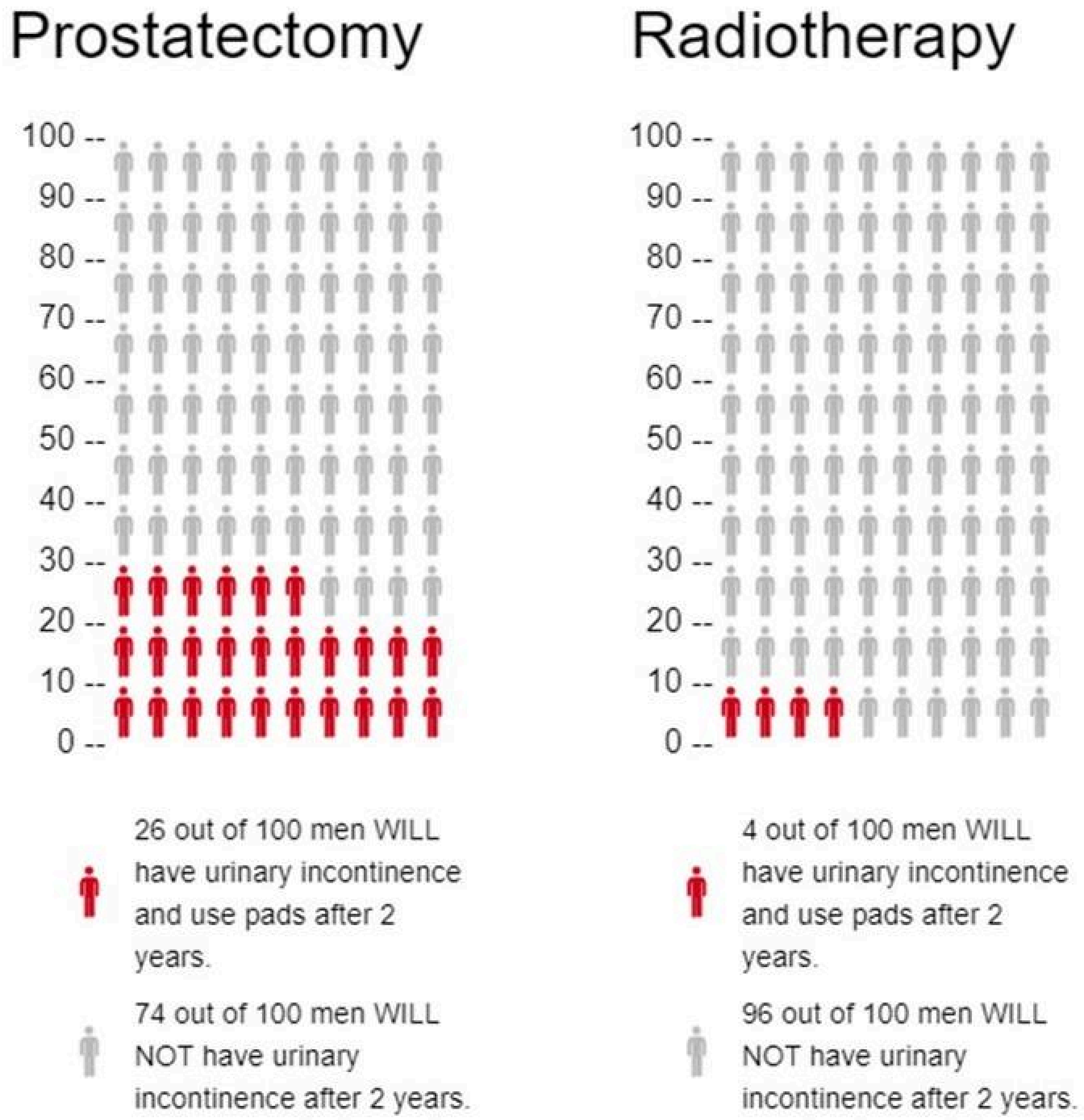
Prostatectomy
Radiotherapy
100 --
90 --
80 --
70 --
60 --
50 --
40 --
30 --
20 --
10 --
0 --
26 out of 100 men WILL have urinary incontinence and use pads after 2 years.
74 out of 100 men WILL NOT have urinary incontinence after 2 years.
4 out of 100 men WILL have urinary incontinence and use pads after 2 years.
96 out of 100 men WILL NOT have urinary incontinence after 2 years.

Bowel control

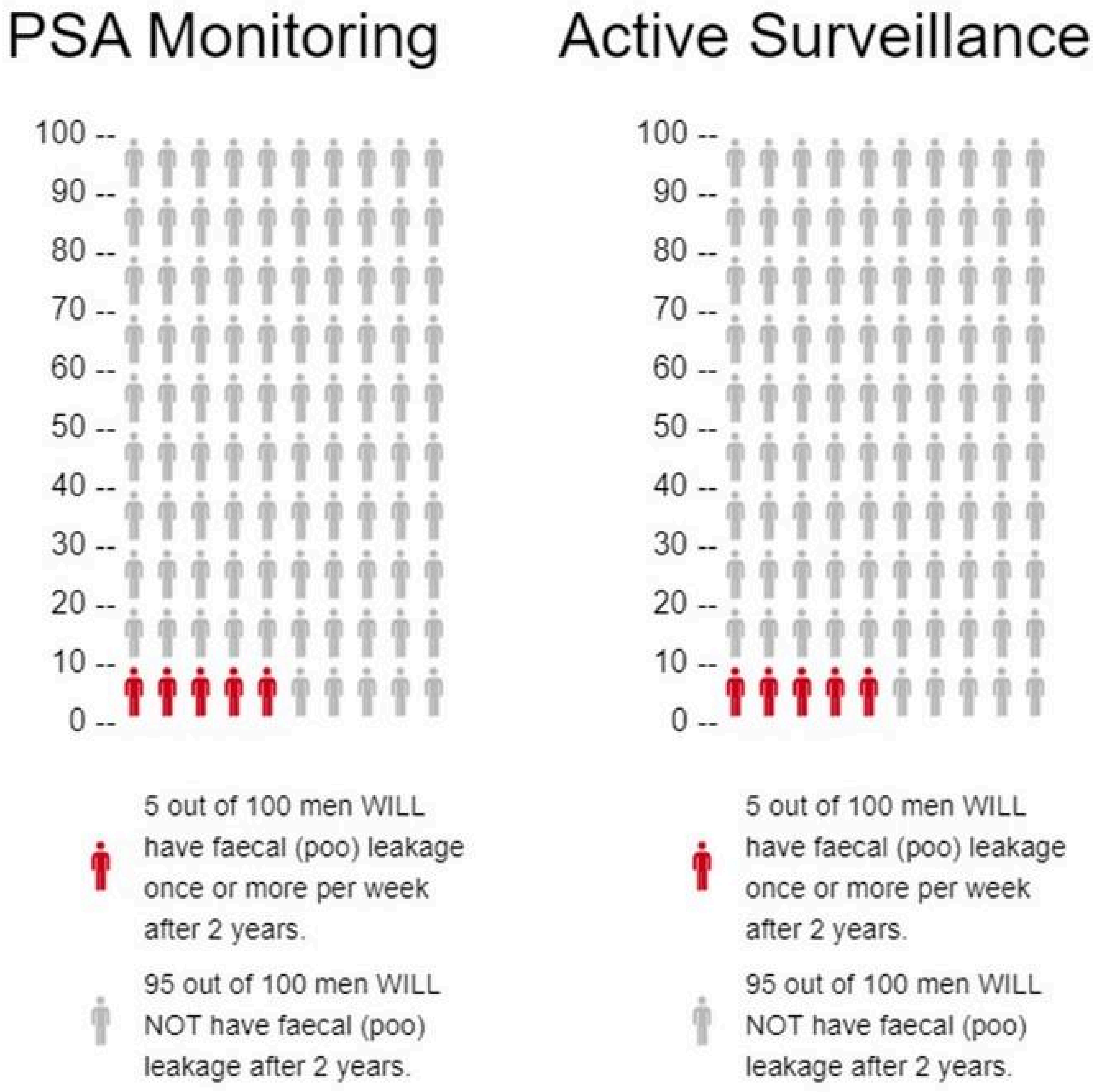

Prostatectomy

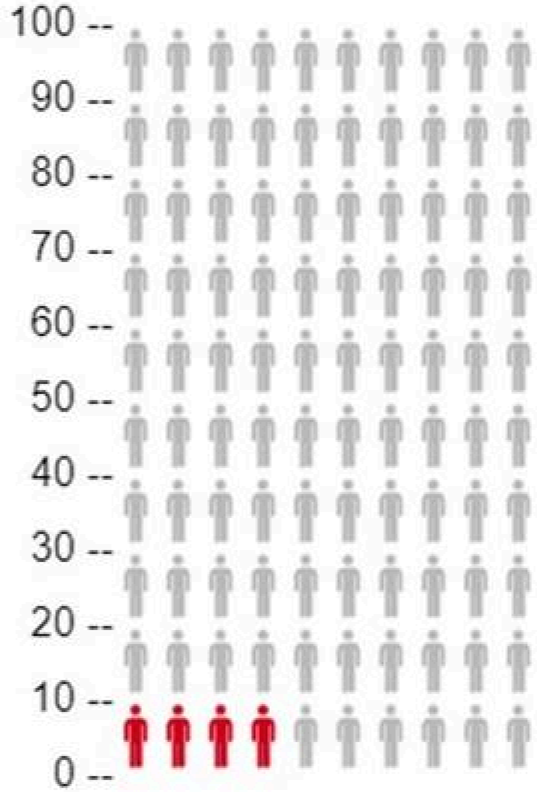

4 out of 100 men WILL have faecal (poo) leakage once or more per week after 2 years.

96 out of 100 men WILL NOT have faecal (poo) leakage after 2 years.

Radiotherapy

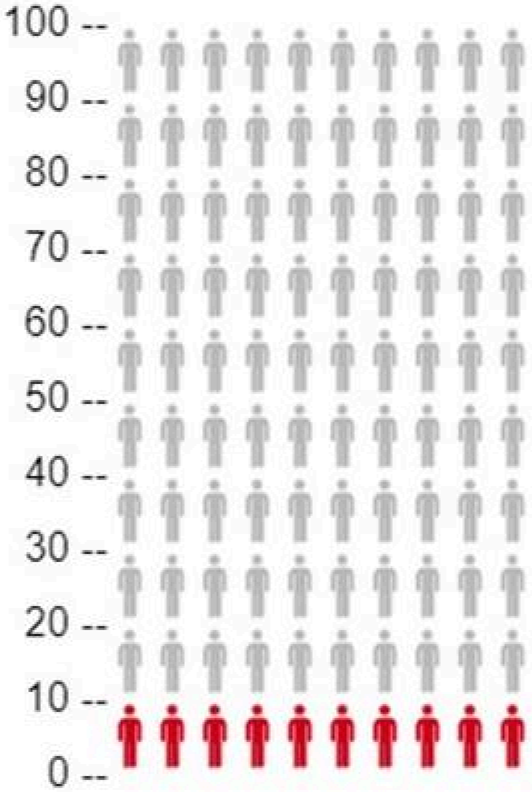

10 out of 100 men WILL have faecal (poo) leakage once or more per week after 2 years.

90 out of 100 men WILL NOT have faecal (poo) leakage after 2 years.

Spread

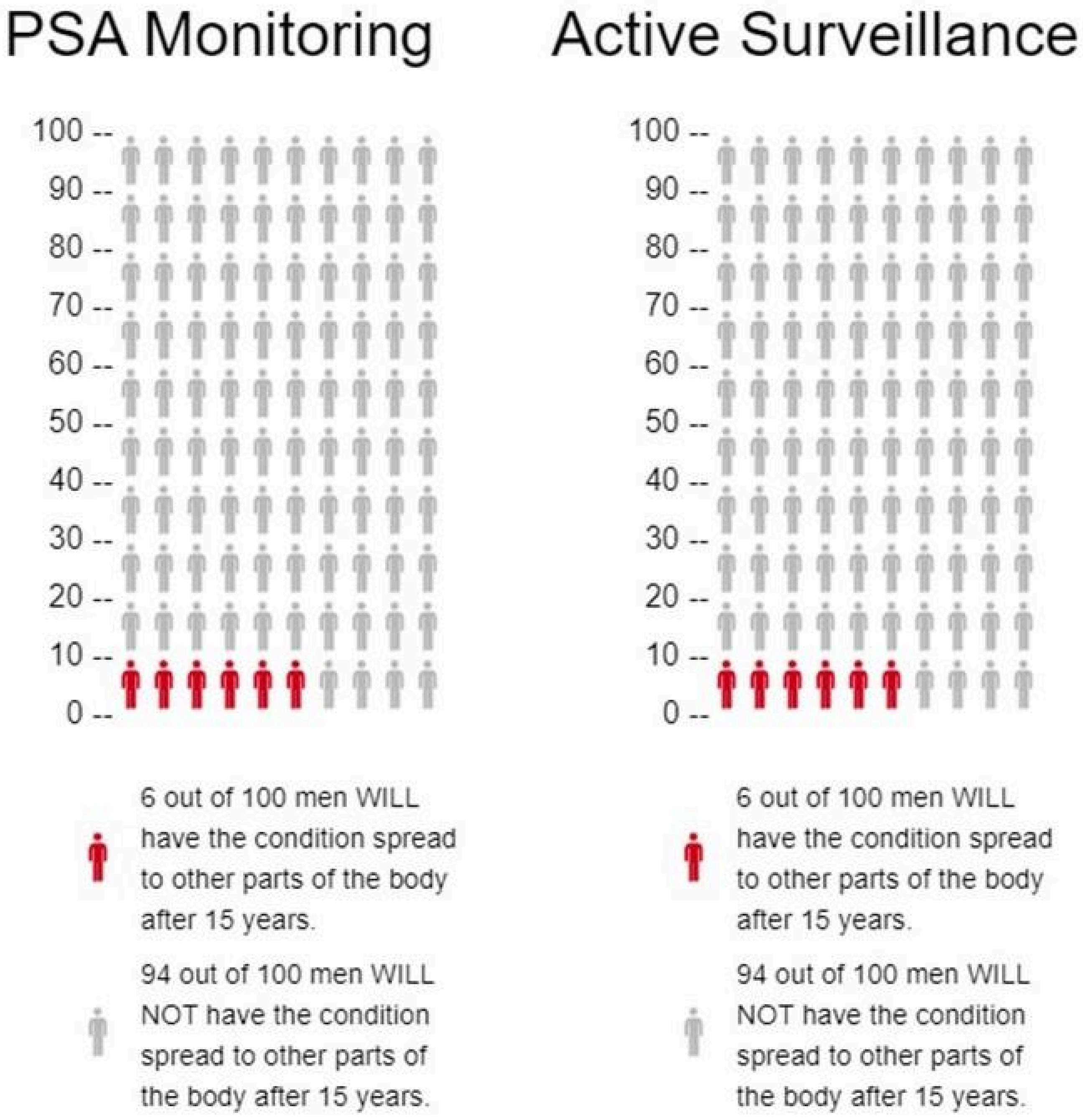
PSA Monitoring
Active Surveillance
100 --
90 --
80 --
70 --
60 --
50 --
40 --
30 --
20 --
10 --
0 --
6 out of 100 men WILL have the condition spread to other parts of the body after 15 years.
94 out of 100 men WILL NOT have the condition spread to other parts of the body after 15 years.
6 out of 100 men WILL have the condition spread to other parts of the body after 15 years.
94 out of 100 men WILL NOT have the condition spread to other parts of the body after 15 years.

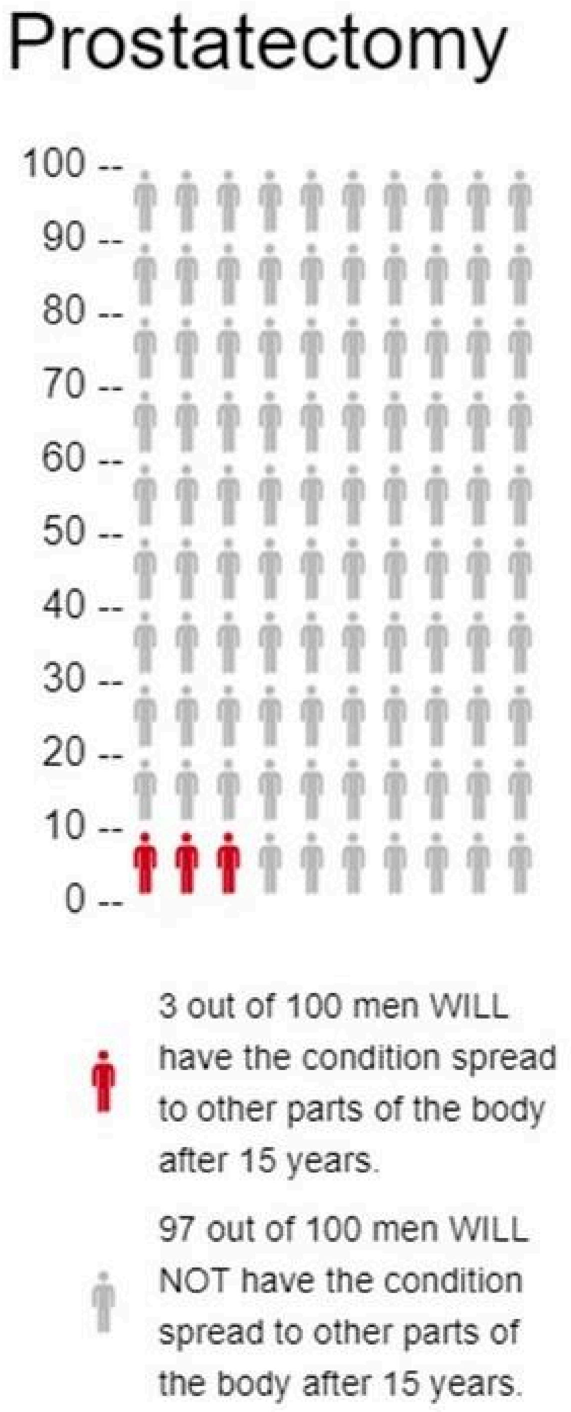

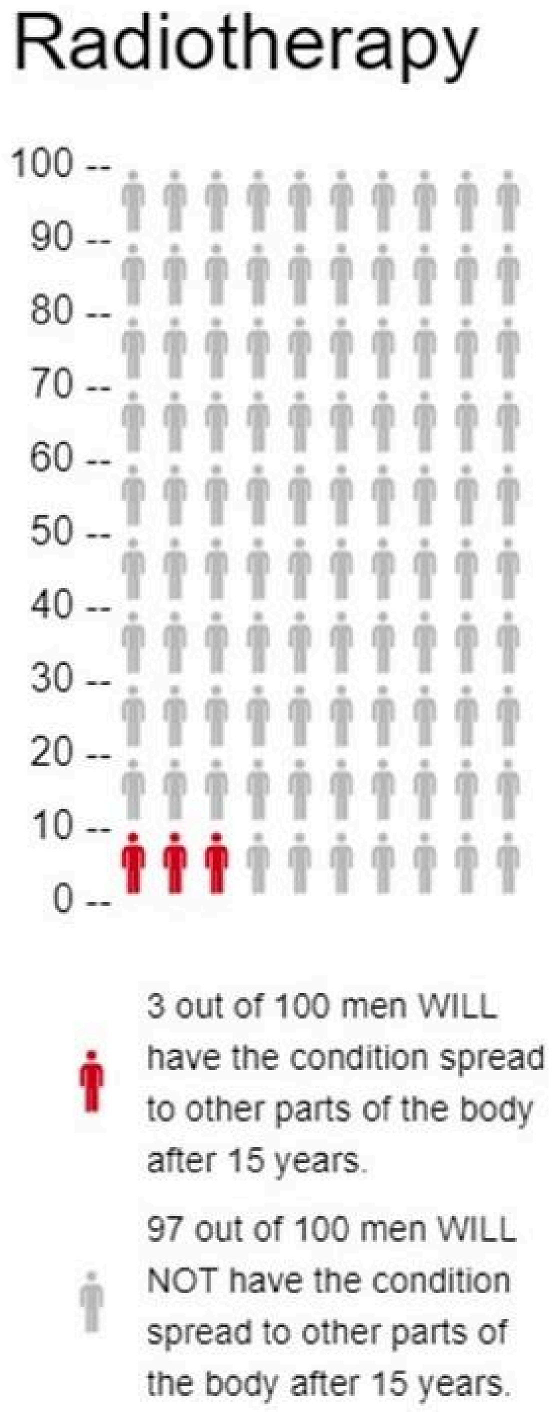

Hypothetical - Label 2 + Low Information - Partners

Please read the information below and answer the questions that follow. You are asked to imagine as if the following scenario is true. Please answer how you would feel or react if you were in this situation, to the best of your ability.

You are at the doctor (GP) with your partner who recently had a prostate biopsy (sample of their prostate). This was because of a raised prostate specific antigen (PSA) result on a blood test.

The doctor has the biopsy test results and says:
“We found a small focus of **low-risk prostate lesion**.”

After giving your partner these test results, the doctor explains that there are four options. These are:

Option 1. PSA monitoring: where your partner keeps visiting the doctor to get check-ups and tests. Your partner will be monitored at regular points in time with PSA blood tests. This is to monitor the way the low-risk prostate lesion behaves. If it shows signs of growing, then treatment can be started.

Option 2. Active surveillance: where your partner keeps visiting the doctor to get check-ups and tests. Your partner will be monitored at regular points in time with PSA blood tests, MRI scans, and prostate biopsies. This is to monitor the way the low-risk prostate lesion behaves. If it shows signs of growing, then treatment can be started.

Option 3. Radical prostatectomy: where your partner has a surgical procedure to remove the prostate. This includes the low-risk prostate lesion.

Option 4. Radiotherapy: where your partner has a non-surgical procedure on the prostate. The prostate is treated with radiation to destroy the low-risk prostate lesion.

Studies have reported:

- For every 100 men found to have a low-risk prostate lesion, 2 will die from this over the next 15 years, regardless of the treatment.

Different treatments have different advantages and disadvantages.

Hypothetical - Label 2 + High Information - Partners

Please read the information below and answer the questions that follow. You are asked to imagine as if the following scenario is true. Please answer how you would feel or react if you were in this situation, to the best of your ability.

You are at the doctor (GP) with your partner who recently had a prostate biopsy (sample of their prostate). This was because of a raised prostate specific antigen (PSA) result on a blood test.

The doctor has the biopsy test results and says:
“We found a small focus of **low-risk prostate lesion**.”

After giving your partner these test results, the doctor explains that there are four options. These are:

Option 1. PSA monitoring: where your partner keeps visiting the doctor to get check-ups and tests. Your partner will be monitored at regular points in time with PSA blood tests. This is to monitor the way the low-risk prostate lesion behaves. If it shows signs of growing, then treatment can be started.

Option 2. Active surveillance: where your partner keeps visiting the doctor to get check-ups and tests. Your partner will be monitored at regular points in time with PSA blood tests, MRI scans, and prostate biopsies. This is to monitor the way the low-risk prostate lesion behaves. If it shows signs of growing, then treatment can be started.

Option 3. Radical prostatectomy: where your partner has a surgical procedure to remove the prostate. This includes the low-risk prostate lesion.

Option 4. Radiotherapy: where your partner has a non-surgical procedure on the prostate. The prostate is treated with radiation to destroy the low-risk prostate lesion.

Studies have reported:

- For every 100 men found to have a low-risk prostate lesion, 2 will die from this over the next 15 years, regardless of the treatment.

Different treatments have different advantages and disadvantages.

Erectile dysfunction
(difficulty getting and keeping an erection)

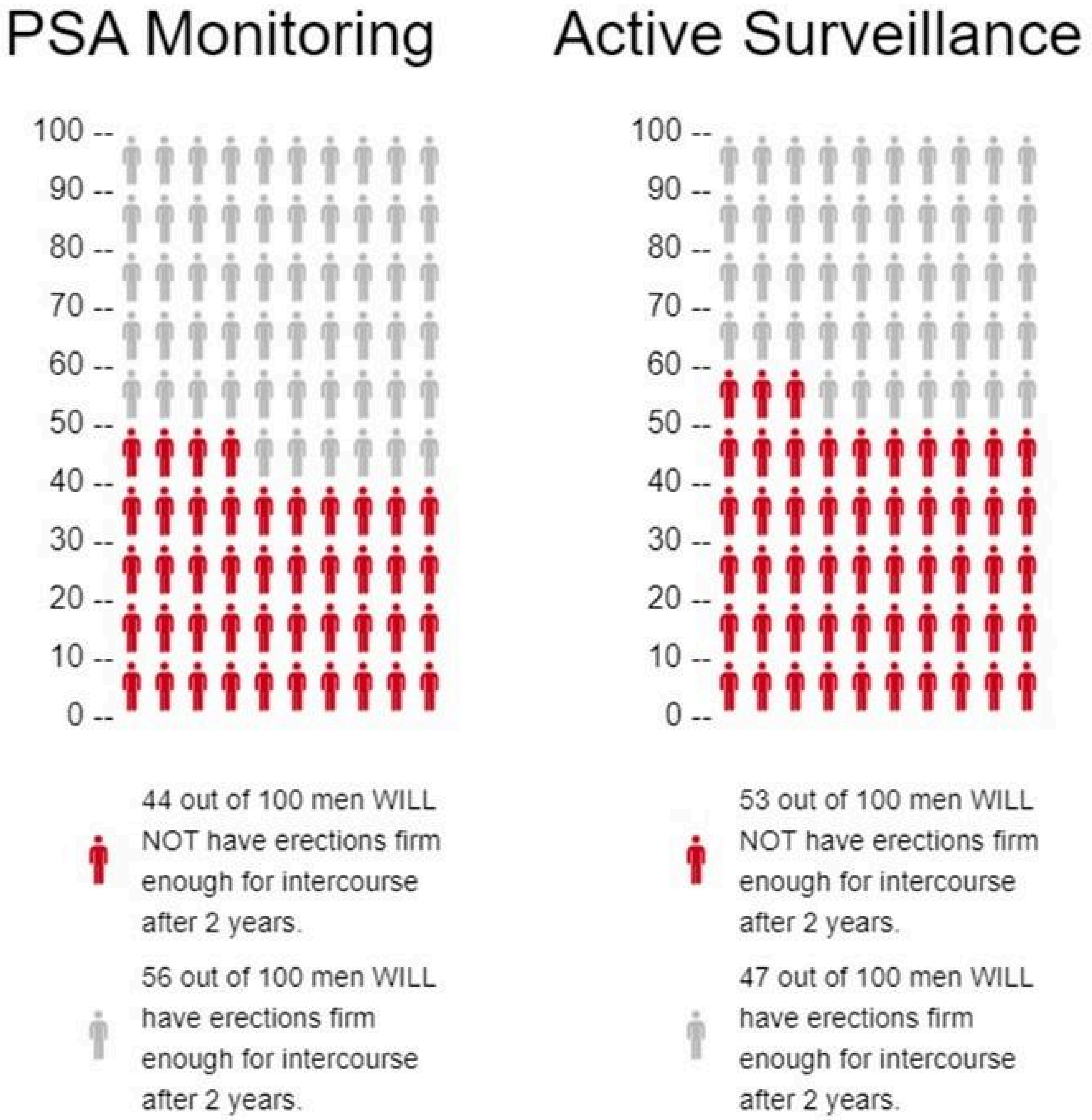
PSA Monitoring
Active Surveillance
100 --
90 --
80 --
70 --
60 --
50 --
40 --
30 --
20 --
10 --
0 --
44 out of 100 men WILL NOT have erections firm enough for intercourse after 2 years.
56 out of 100 men WILL have erections firm enough for intercourse after 2 years.
53 out of 100 men WILL NOT have erections firm enough for intercourse after 2 years.
47 out of 100 men WILL have erections firm enough for intercourse after 2 years.

Prostatectomy

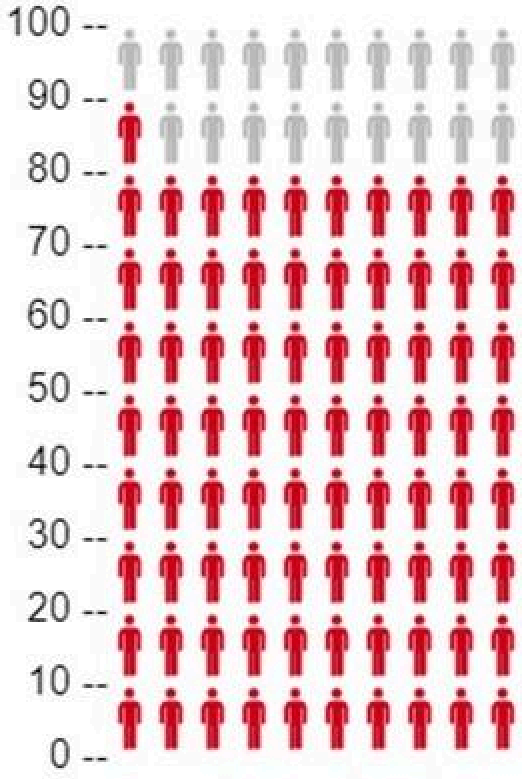

81 out of 100 men WILL NOT have erections firm enough for intercourse after 2 years.

19 out of 100 men WILL have erections firm enough for intercourse after 2 years.

Radiotherapy

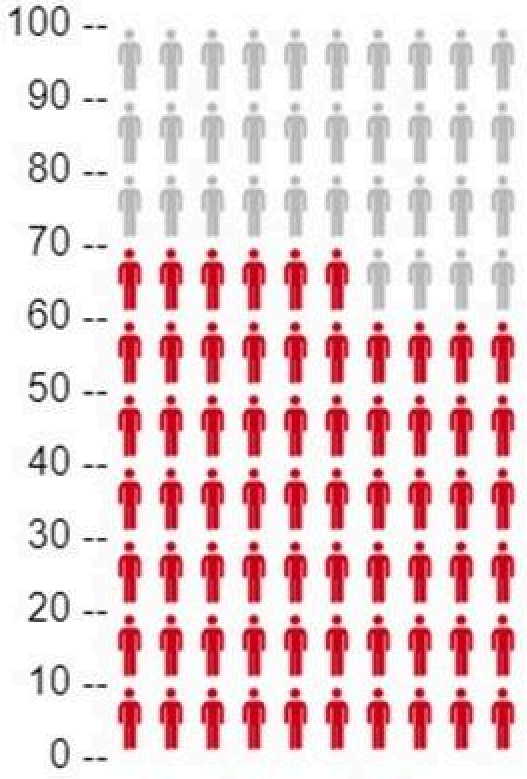

66 out of 100 men WILL NOT have erections firm enough for intercourse after 2 years.

34 out of 100 men WILL have erections firm enough for intercourse after 2 years.

Bladder control

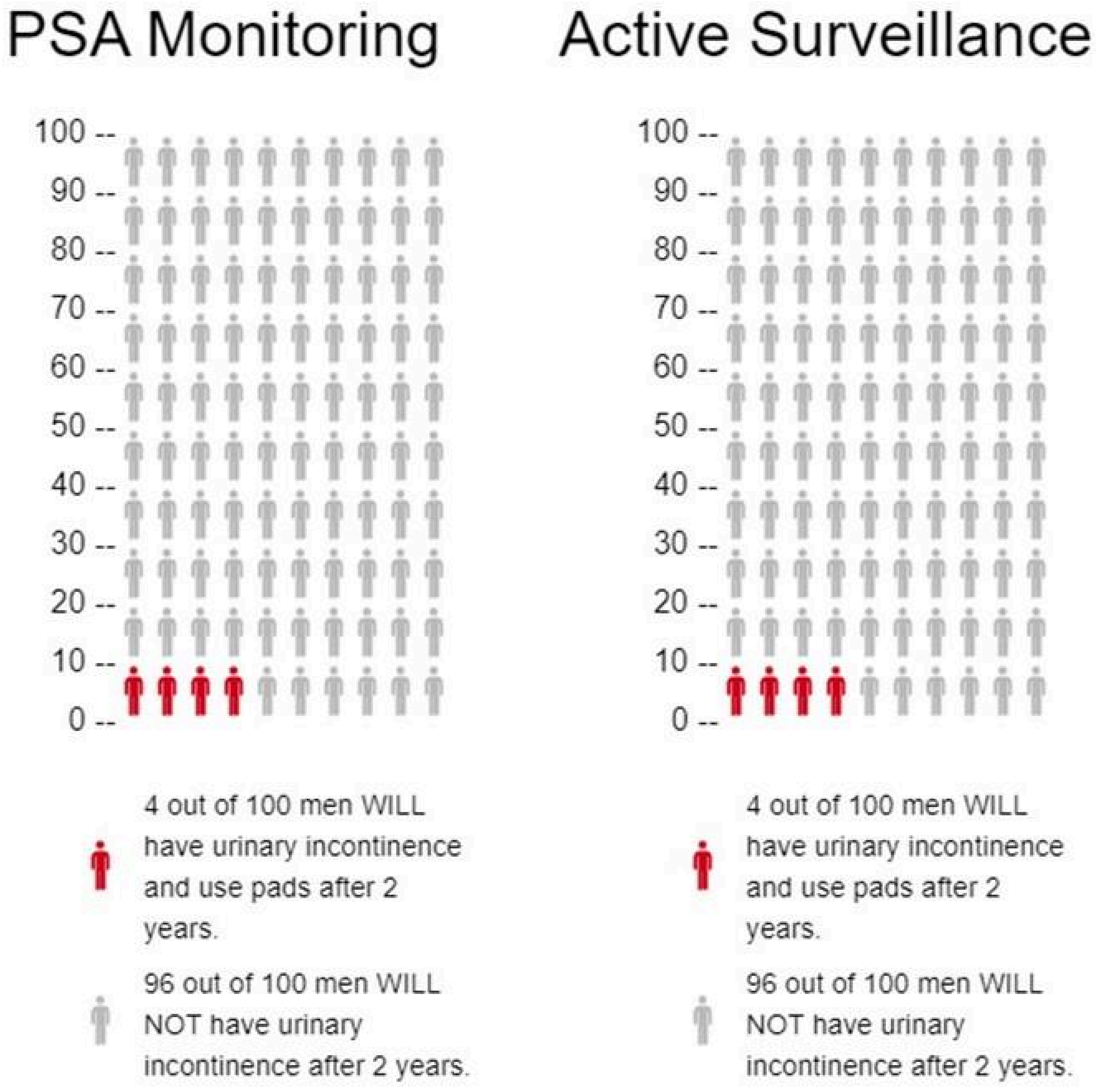

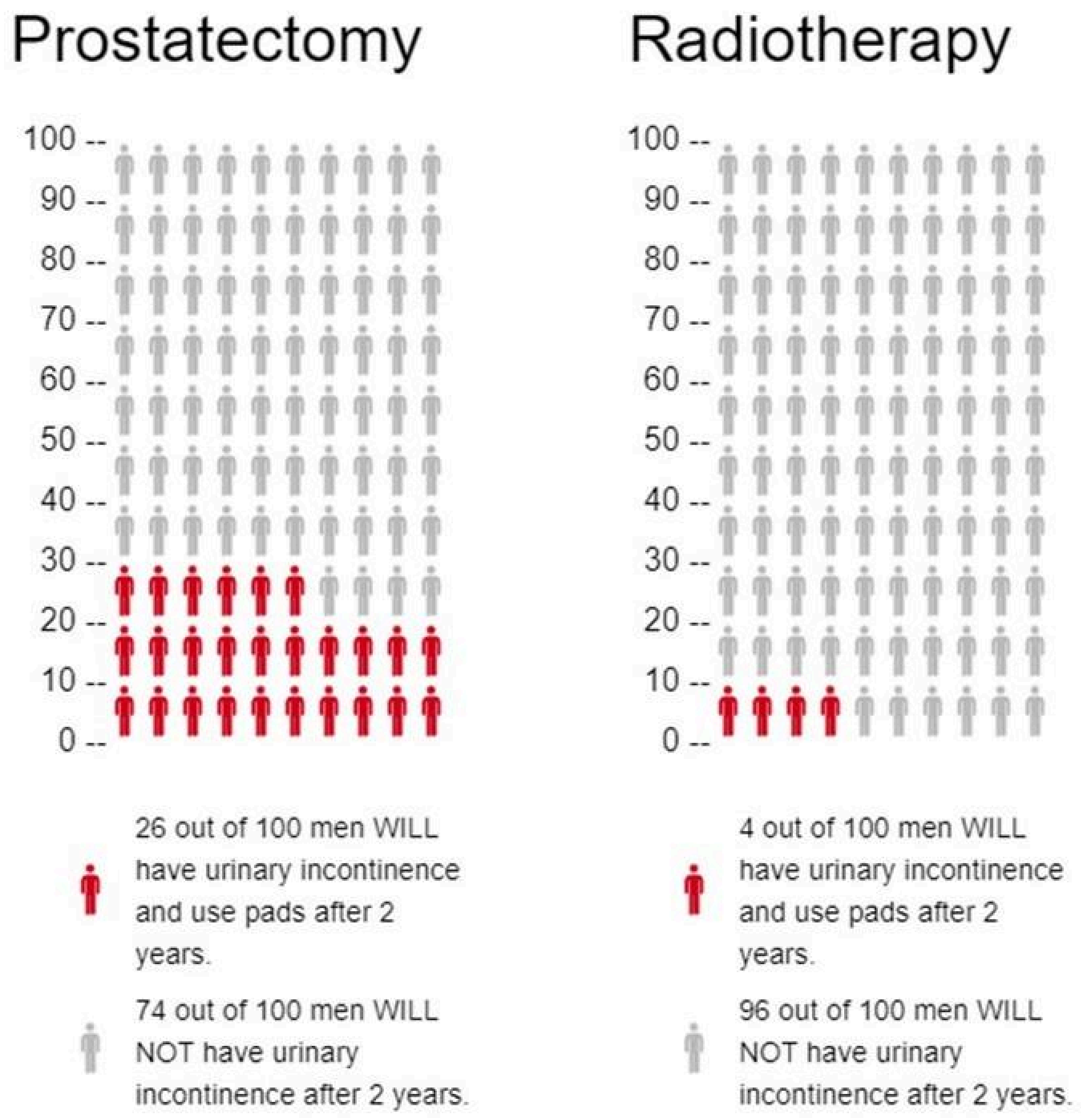
Prostatectomy
100 --
90 --
80 --
70 --
60 --
50 --
40 --
30 --
20 --
10 --
0 --
26 out of 100 men WILL have urinary incontinence and use pads after 2 years.
74 out of 100 men WILL NOT have urinary incontinence after 2 years.
Radiotherapy
100 --
90 --
80 --
70 --
60 --
50 --
40 --
30 --
20 --
10 --
0 --
4 out of 100 men WILL have urinary incontinence and use pads after 2 years.
96 out of 100 men WILL NOT have urinary incontinence after 2 years.

Bowel control

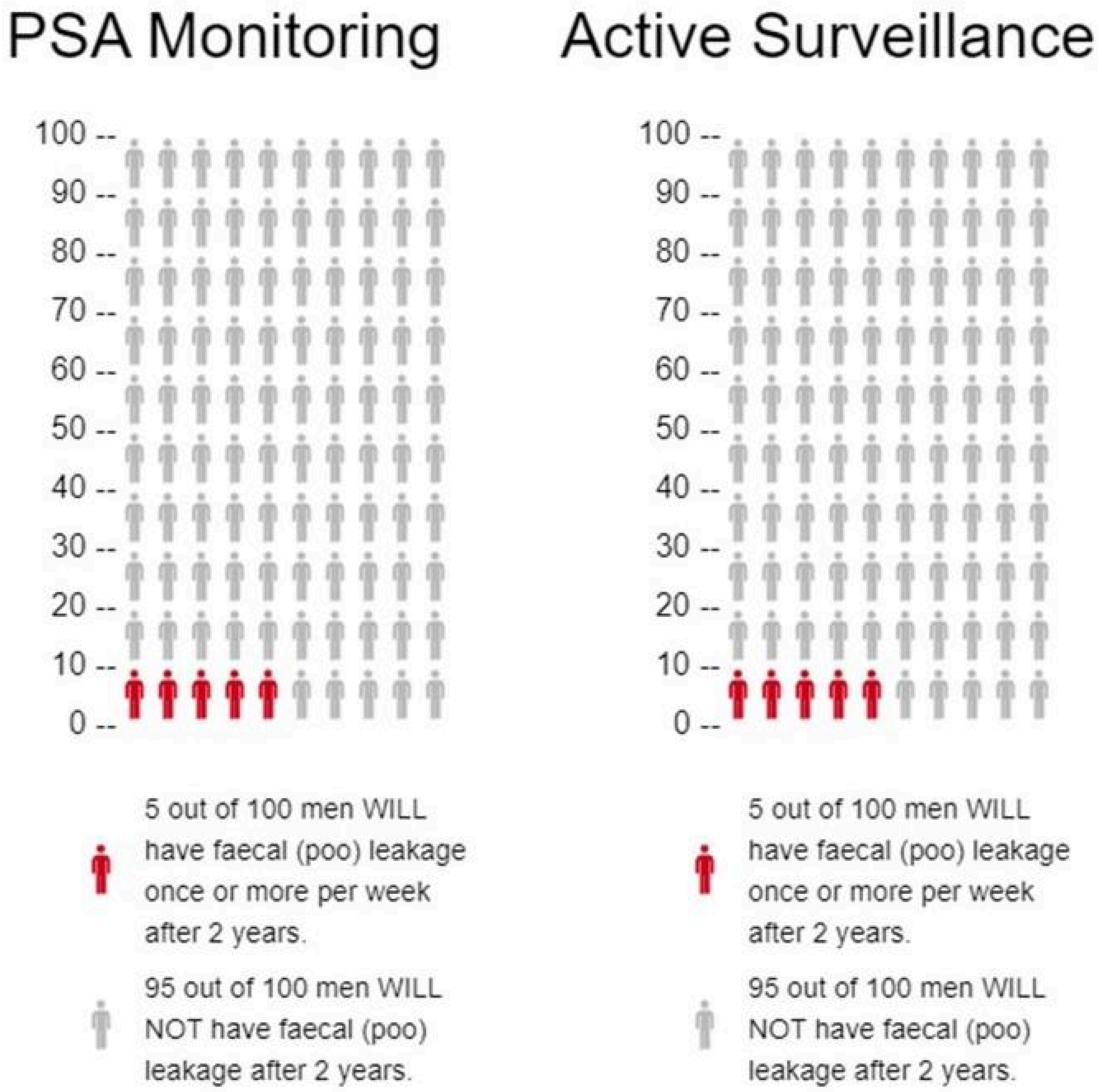

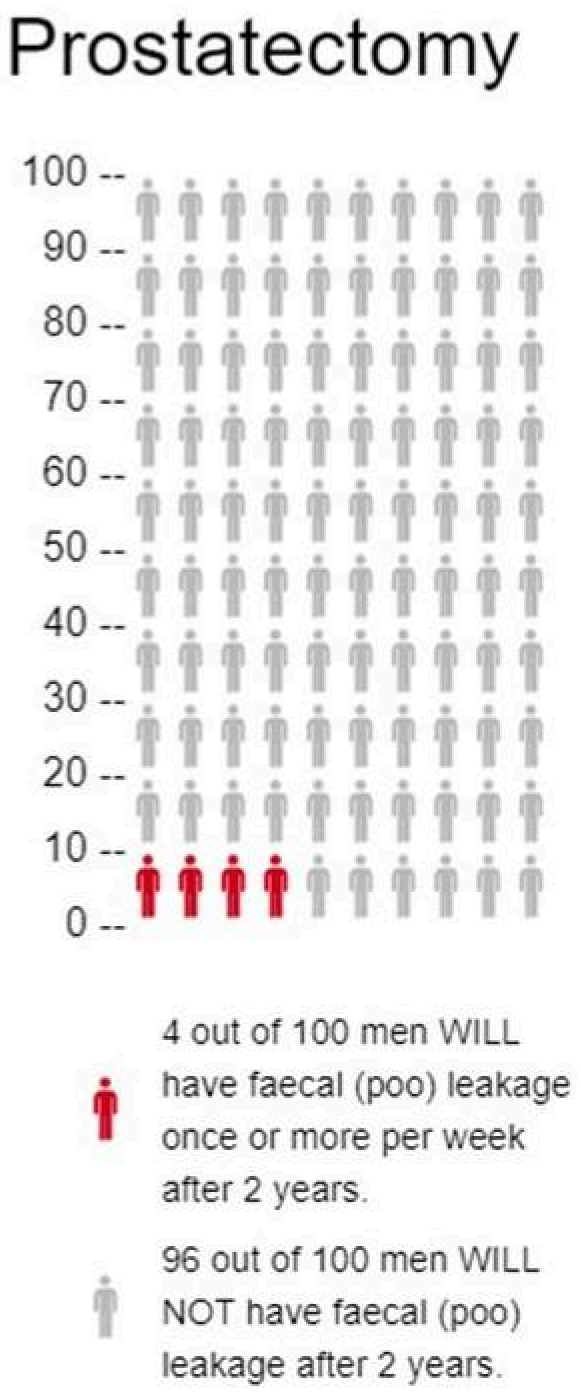
Prostatectomy
100 --
90 --
80 --
70 --
60 --
50 --
40 --
30 --
20 --
10 --
0 --
4 out of 100 men WILL have faecal (poo) leakage once or more per week after 2 years.
96 out of 100 men WILL NOT have faecal (poo) leakage after 2 years.

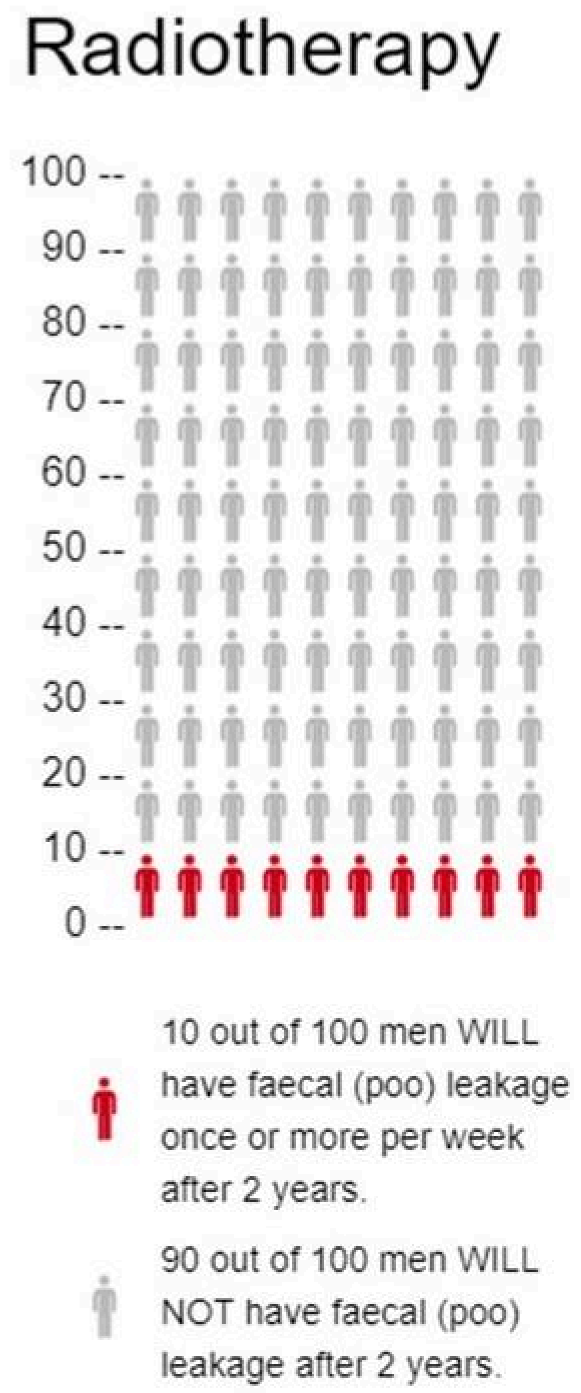
Radiotherapy
100 --
90 --
80 --
70 --
60 --
50 --
40 --
30 --
20 --
10 --
0 --
10 out of 100 men WILL have faecal (poo) leakage once or more per week after 2 years.
90 out of 100 men WILL NOT have faecal (poo) leakage after 2 years.

Spread

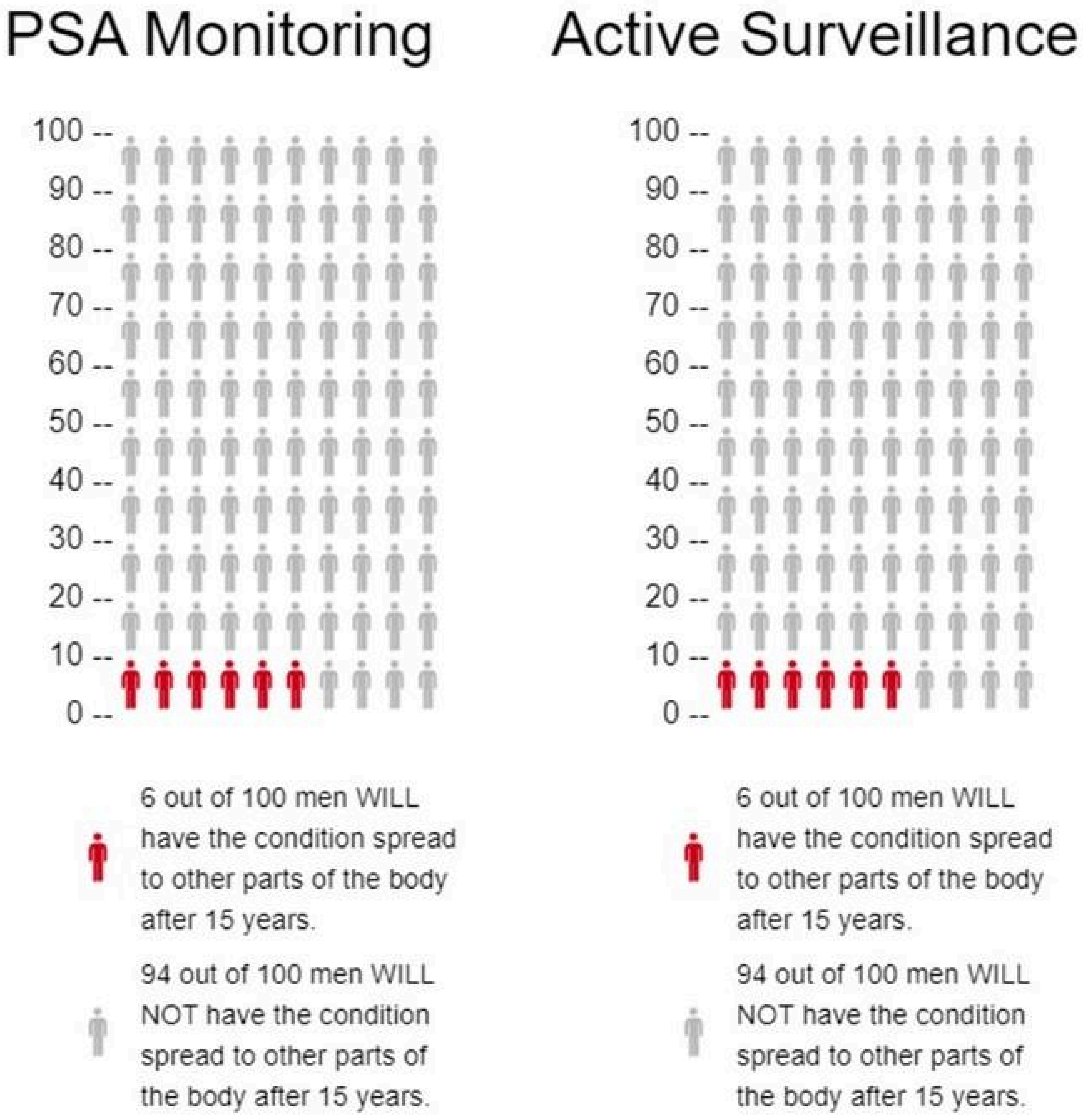
PSA Monitoring
Active Surveillance
100 --
90 --
80 --
70 --
60 --
50 --
40 --
30 --
20 --
10 --
0 --
6 out of 100 men WILL have the condition spread to other parts of the body after 15 years.
94 out of 100 men WILL NOT have the condition spread to other parts of the body after 15 years.
6 out of 100 men WILL have the condition spread to other parts of the body after 15 years.
94 out of 100 men WILL NOT have the condition spread to other parts of the body after 15 years.

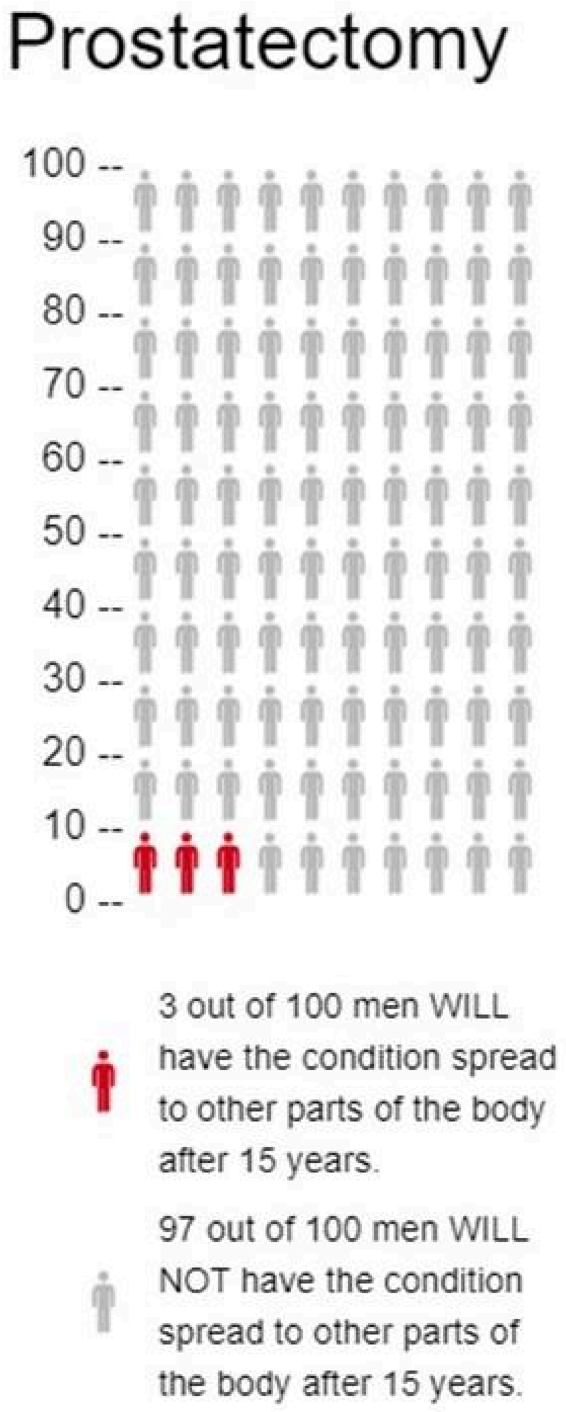

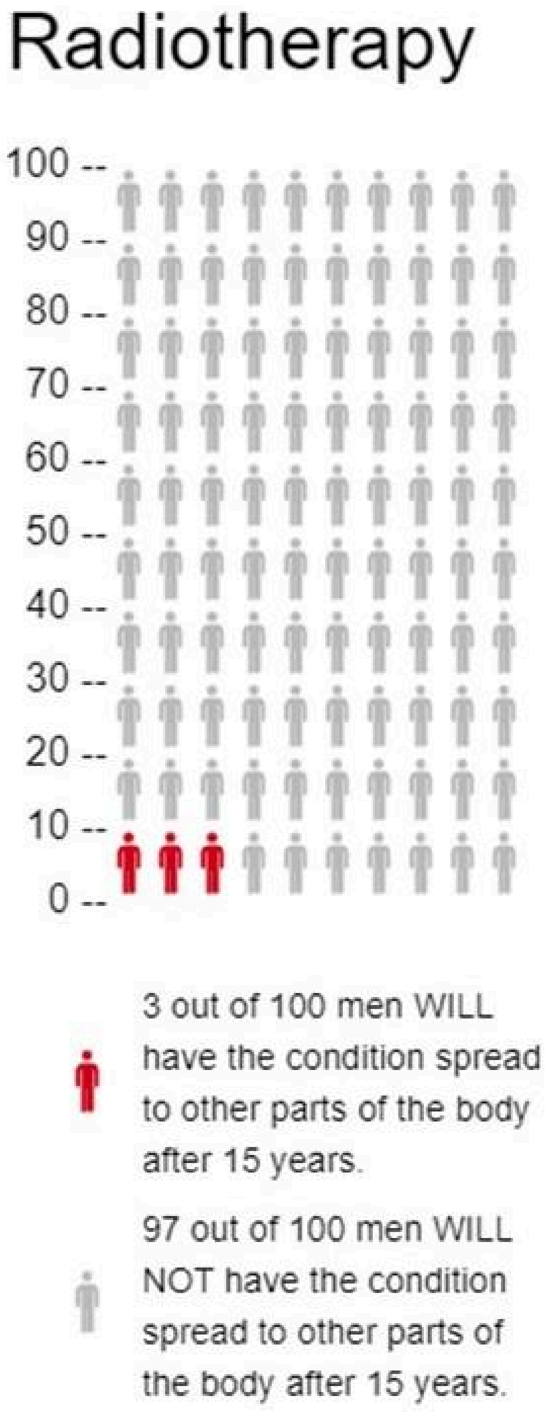

Hypothetical - Control + Low Information - Men

Please read the information below and answer the questions that follow. You are asked to imagine as if the following scenario is true. Please answer how you would feel or react if you were in this situation, to the best of your ability.

You are at the doctor (GP) after you recently had a prostate biopsy (sample of your prostate). This was because of a raised prostate specific antigen (PSA) result on a blood test.

The doctor has the biopsy test results and says:
“We found a small focus of **low-risk prostate cancer, grade group 1**.”

After giving you these test results, the doctor explains that there are four options. These are:

Option 1. PSA monitoring: where you keep visiting the doctor to get check-ups and tests. You will be monitored at regular points in time with PSA blood tests. This is to monitor the way the low-risk prostate cancer (grade group 1) behaves. If it shows signs of growing, then treatment can be started.

Option 2. Active surveillance: where you keep visiting the doctor to get check-ups and tests. You will be monitored at regular points in time with PSA blood tests, MRI scans, and prostate biopsies. This is to monitor the way the low-risk prostate cancer (grade group 1) behaves. If it shows signs of growing, then treatment can be started.

Option 3. Radical prostatectomy: where you have a surgical procedure to remove the prostate. This includes the low-risk prostate cancer (grade group 1).

Option 4. Radiotherapy: where you have a non-surgical procedure on the prostate. The prostate is treated with radiation to destroy the low-risk prostate cancer (grade group 1).

Studies have reported:

- For every 100 men found to have low-risk prostate cancer (grade group 1), 2 will die from this over the next 15 years, regardless of the treatment.

Different treatments have different advantages and disadvantages.

Hypothetical - Control + High Information - Men

Please read the information below and answer the questions that follow. You are asked to imagine as if the following scenario is true. Please answer how you would feel or react if you were in this situation, to the best of your ability.

You are at the doctor (GP) after you recently had a prostate biopsy (sample of your prostate). This was because of a raised prostate specific antigen (PSA) result on a blood test.

The doctor has the biopsy test results and says:
"We found a small focus of **low-risk prostate cancer, grade group 1**."

After giving you these test results, the doctor explains that there are four options. These are:

Option 1. PSA monitoring: where you keep visiting the doctor to get check-ups and tests. You will be monitored at regular points in time with PSA blood tests. This is to monitor the way the low-risk prostate cancer (grade group 1) behaves. If it shows signs of growing, then treatment can be started.

Option 2. Active surveillance: where you keep visiting the doctor to get check-ups and tests. You will be monitored at regular points in time with PSA blood tests, MRI scans, and prostate biopsies. This is to monitor the way the low-risk prostate cancer (grade group 1) behaves. If it shows signs of growing, then treatment can be started.

Option 3. Radical prostatectomy: where you have a surgical procedure to remove the prostate. This includes the low-risk prostate cancer (grade group 1).

Option 4. Radiotherapy: where you have a non-surgical procedure on the prostate. The prostate is treated with radiation to destroy the low-risk prostate cancer (grade group 1).

Studies have reported:

- For every 100 men found to have low-risk prostate cancer (grade group 1), 2 will die from this over the next 15 years, regardless of the treatment.

Different treatments have different advantages and disadvantages.
Please consider the diagrams on the next page for details:

Erectile dysfunction

(difficulty getting and keeping an erection)

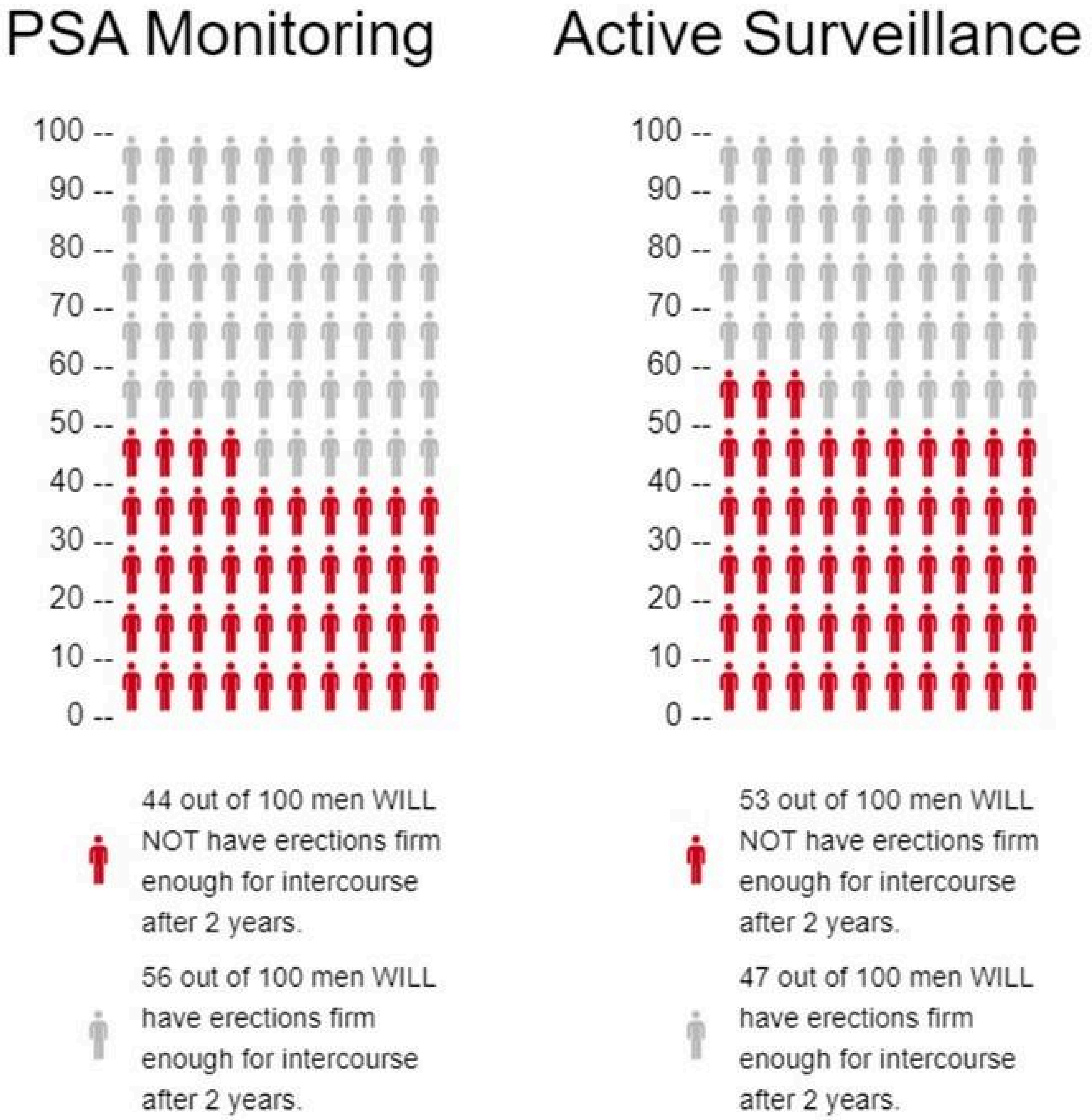
PSA Monitoring
Active Surveillance
100 --
90 --
80 --
70 --
60 --
50 --
40 --
30 --
20 --
10 --
0 --
44 out of 100 men WILL NOT have erections firm enough for intercourse after 2 years.
56 out of 100 men WILL have erections firm enough for intercourse after 2 years.
53 out of 100 men WILL NOT have erections firm enough for intercourse after 2 years.
47 out of 100 men WILL have erections firm enough for intercourse after 2 years.

Prostatectomy

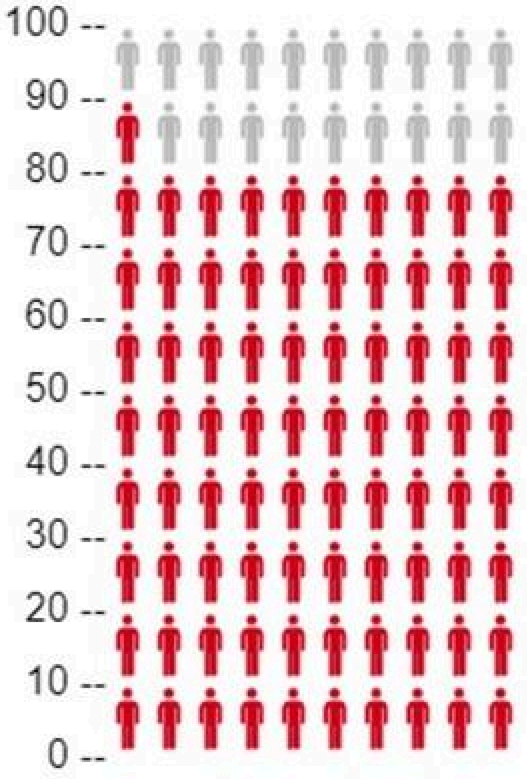

81 out of 100 men WILL NOT have erections firm enough for intercourse after 2 years.

19 out of 100 men WILL have erections firm enough for intercourse after 2 years.

Radiotherapy

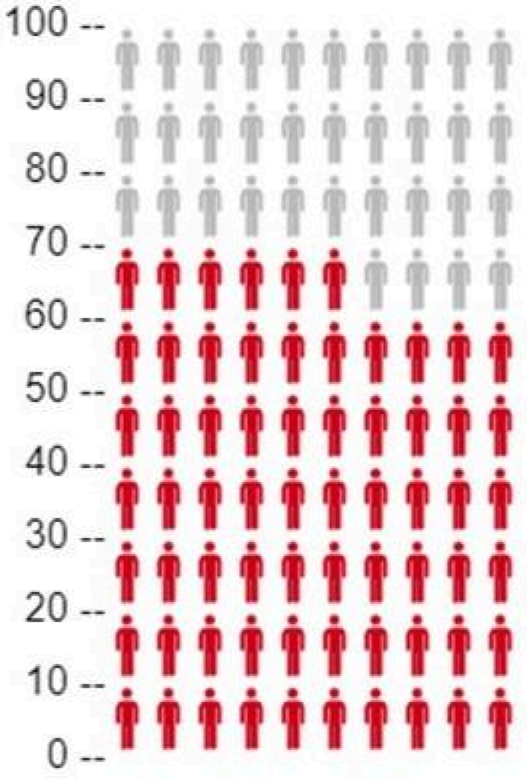

66 out of 100 men WILL NOT have erections firm enough for intercourse after 2 years.

34 out of 100 men WILL have erections firm enough for intercourse after 2 years.

Bladder control

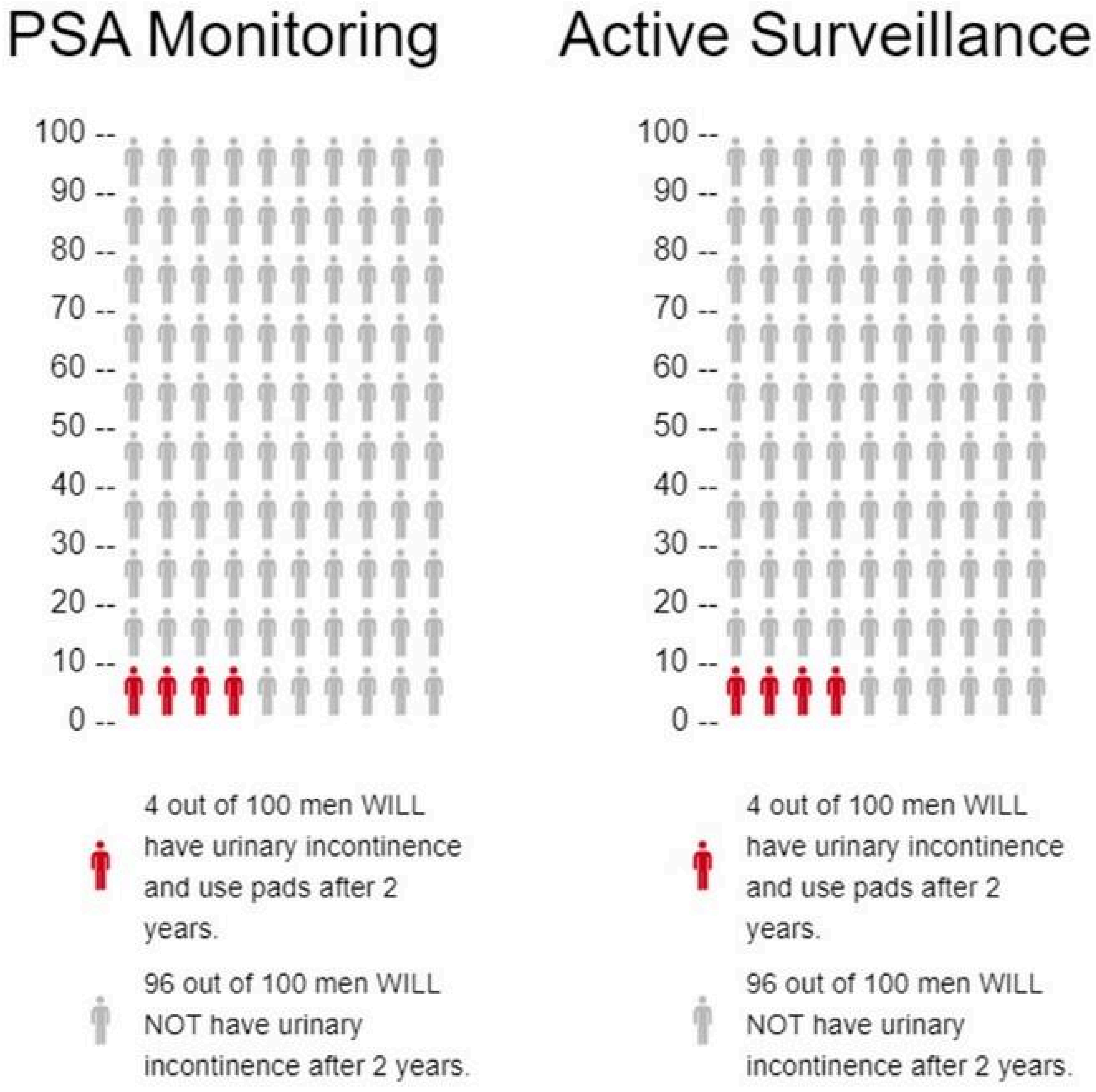

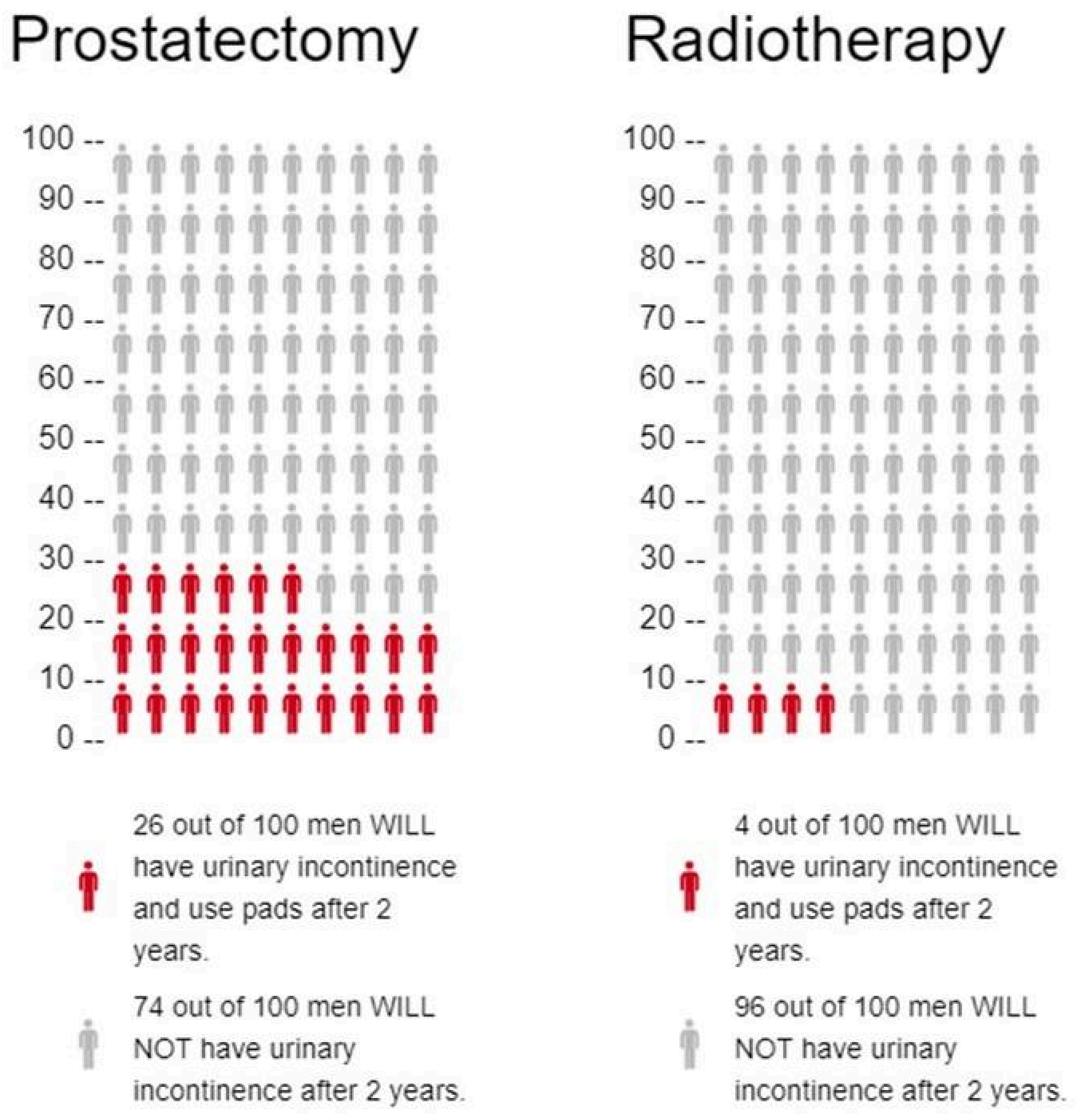
Prostatectomy
Radiotherapy
100 --
90 --
80 --
70 --
60 --
50 --
40 --
30 --
20 --
10 --
0 --
26 out of 100 men WILL have urinary incontinence and use pads after 2 years.
74 out of 100 men WILL NOT have urinary incontinence after 2 years.
4 out of 100 men WILL have urinary incontinence and use pads after 2 years.
96 out of 100 men WILL NOT have urinary incontinence after 2 years.

Bowel control

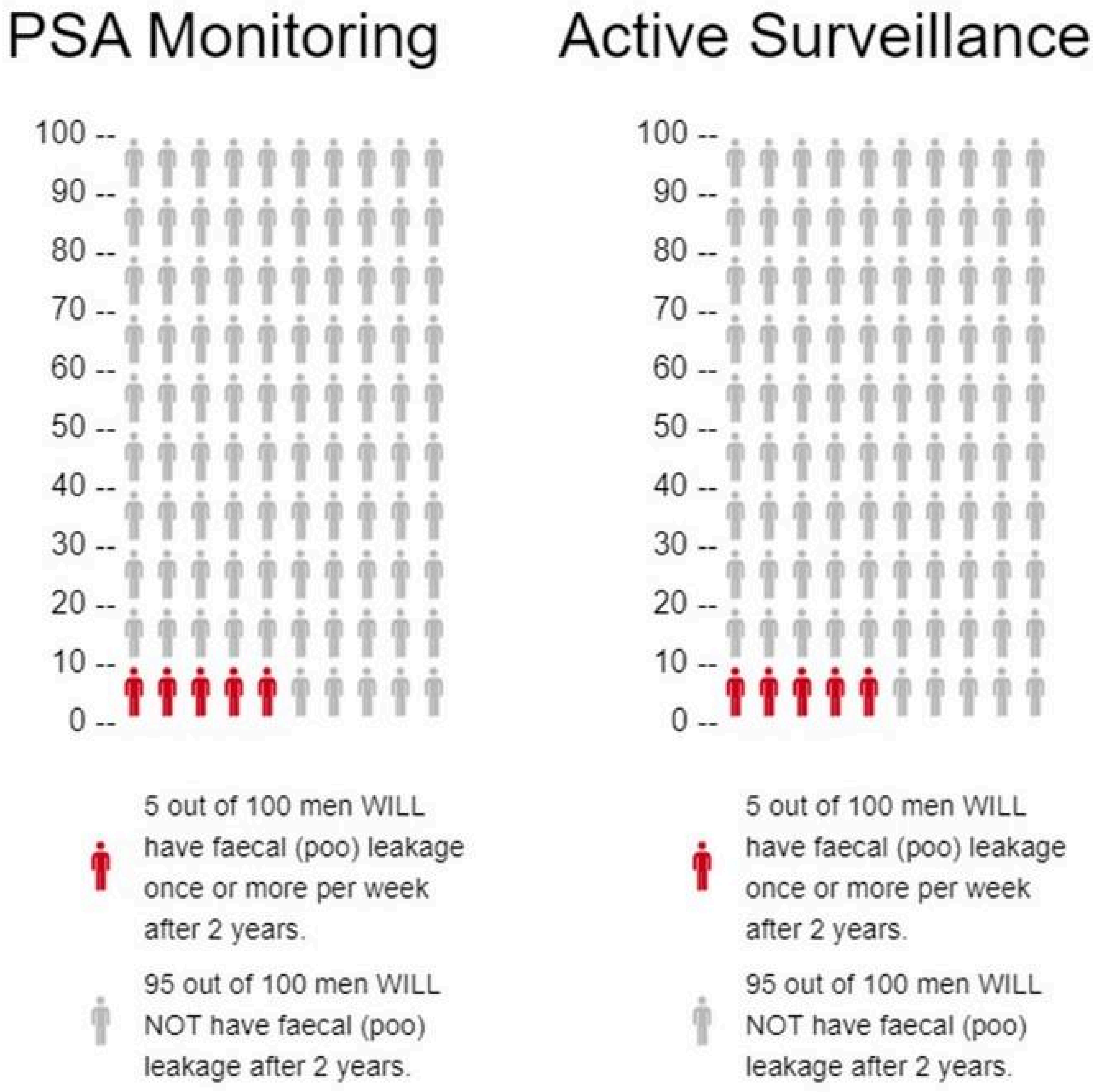

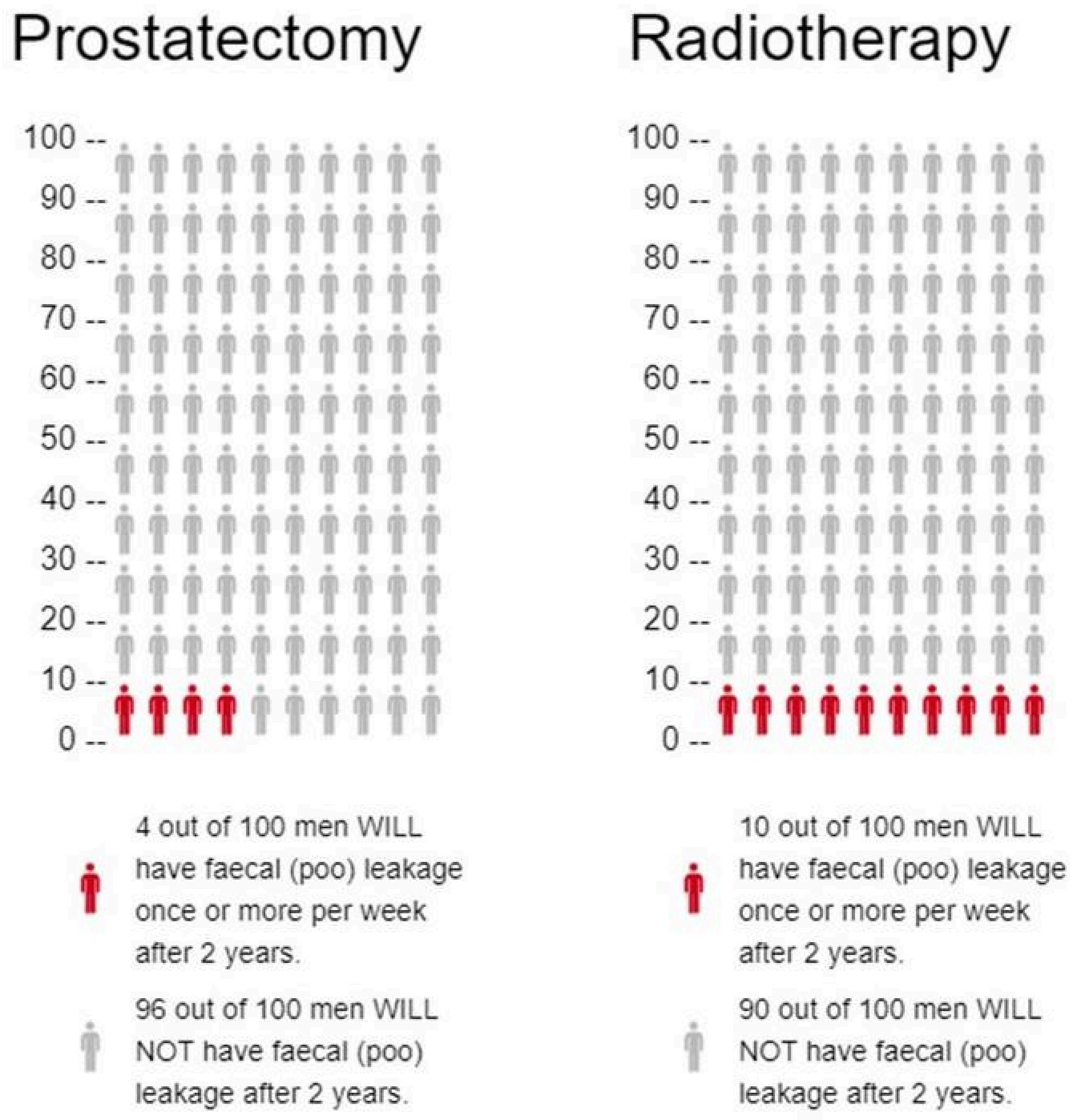
Prostatectomy
Radiotherapy
100 --
90 --
80 --
70 --
60 --
50 --
40 --
30 --
20 --
10 --
0 --
4 out of 100 men WILL have faecal (poo) leakage once or more per week after 2 years.
96 out of 100 men WILL NOT have faecal (poo) leakage after 2 years.
10 out of 100 men WILL have faecal (poo) leakage once or more per week after 2 years.
90 out of 100 men WILL NOT have faecal (poo) leakage after 2 years.

Spread

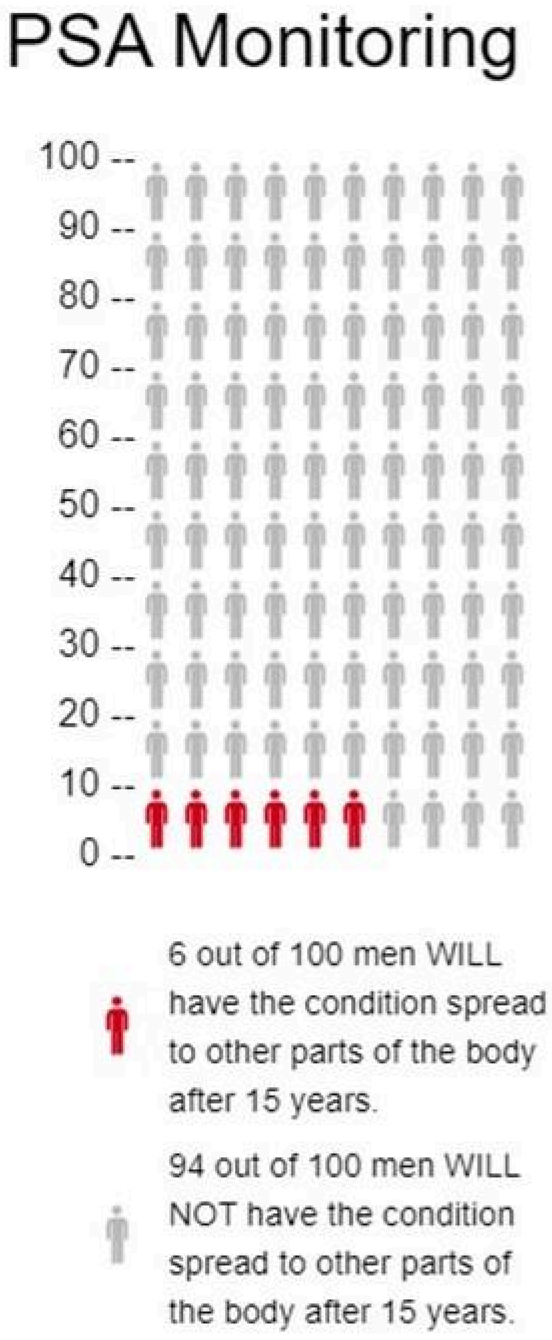
PSA Monitoring
100 --
90 --
80 --
70 --
60 --
50 --
40 --
30 --
20 --
10 --
0 --
6 out of 100 men WILL have the condition spread to other parts of the body after 15 years.
94 out of 100 men WILL NOT have the condition spread to other parts of the body after 15 years.

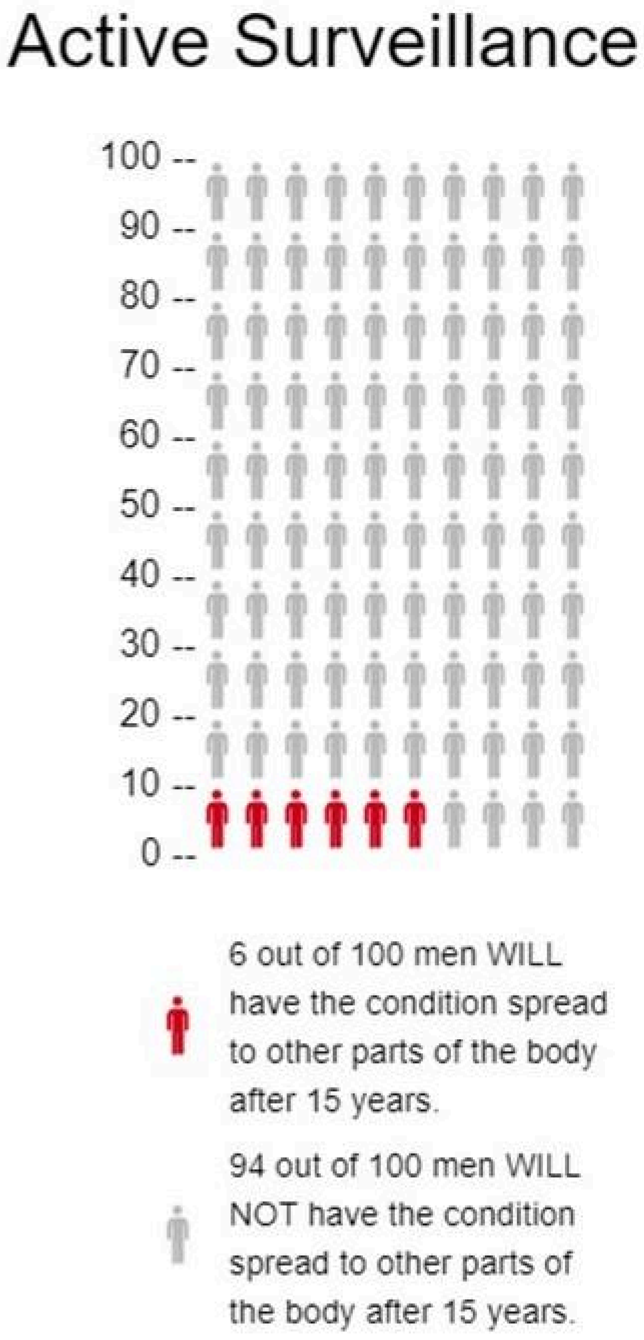
Active Surveillance
100 --
90 --
80 --
70 --
60 --
50 --
40 --
30 --
20 --
10 --
0 --
6 out of 100 men WILL have the condition spread to other parts of the body after 15 years.
94 out of 100 men WILL NOT have the condition spread to other parts of the body after 15 years.

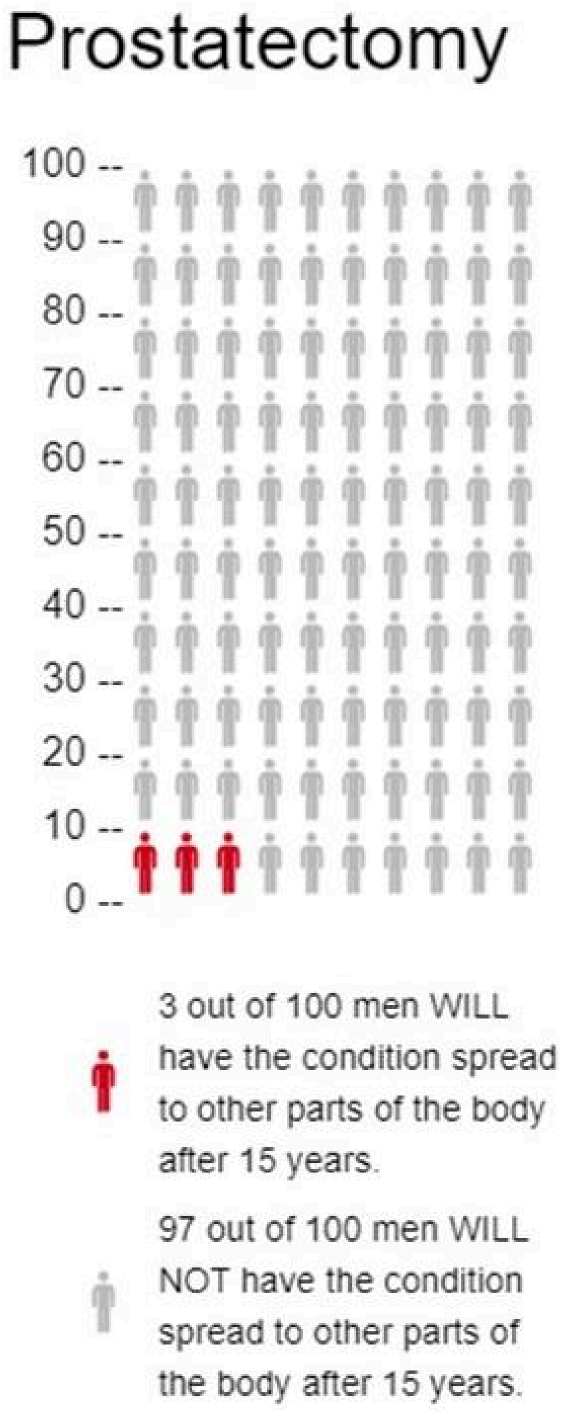

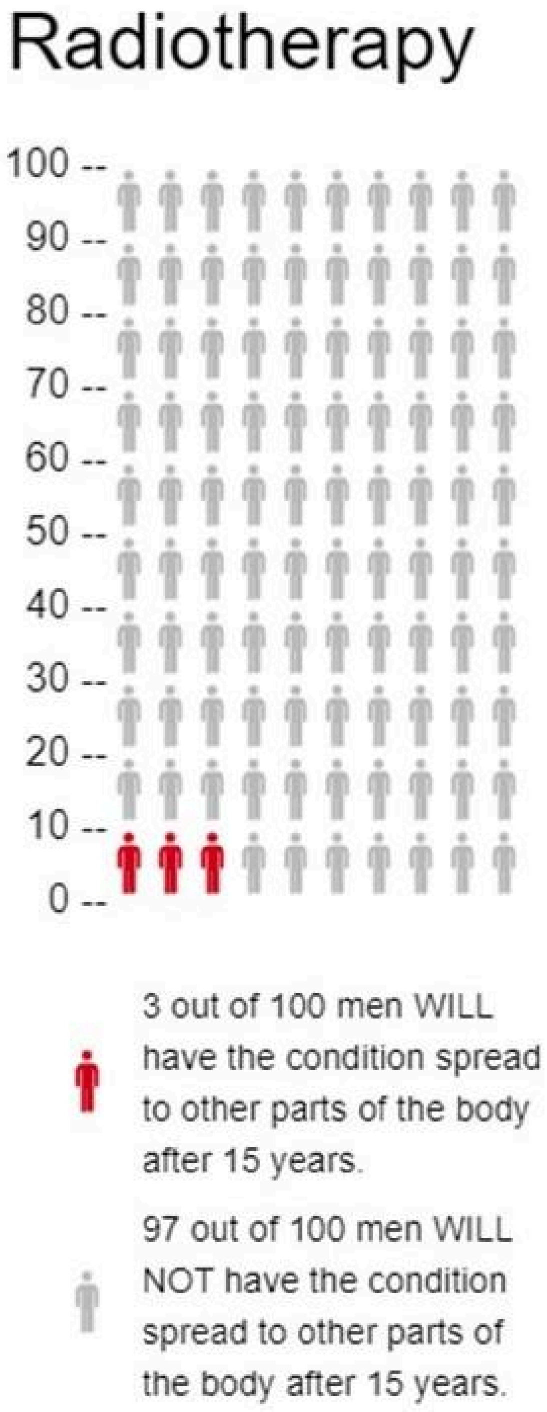

Hypothetical - Label 1 + Low Information - Men

Please read the information below and answer the questions that follow. You are asked to imagine as if the following scenario is true. Please answer how you would feel or react if you were in this situation, to the best of your ability.

You are at the doctor (GP) after you recently had a prostate biopsy (sample of your prostate). This was because of a raised prostate specific antigen (PSA) result on a blood test.

The doctor has the biopsy test results and says:
“We found a small focus of **low-risk prostate neoplasm**.”

After giving you these test results, the doctor explains that there are four options. These are:

Option 1. PSA monitoring: where you keep visiting the doctor to get check-ups and tests. You will be monitored at regular points in time with PSA blood tests. This is to monitor the way the low-risk prostate neoplasm behaves. If it shows signs of growing, then treatment can be started.

Option 2. Active surveillance: where you keep visiting the doctor to get check-ups and tests. You will be monitored at regular points in time with PSA blood tests, MRI scans, and prostate biopsies. This is to monitor the way the low-risk prostate neoplasm behaves. If it shows signs of growing, then treatment can be started.

Option 3. Radical prostatectomy: where you have a surgical procedure to remove the prostate. This includes the low-risk prostate neoplasm.

Option 4. Radiotherapy: where you have a non-surgical procedure on the prostate. The prostate is treated with radiation to destroy the low-risk prostate neoplasm.

Studies have reported:

- For every 100 men found to have a low-risk prostate neoplasm, 2 will die from this over the next 15 years, regardless of the treatment.

Different treatments have different advantages and disadvantages.

Hypothetical - Label 1 + High Information - Men

Please read the information below and answer the questions that follow. You are asked to imagine as if the following scenario is true. Please answer how you would feel or react if you were in this situation, to the best of your ability.

You are at the doctor (GP) after you recently had a prostate biopsy (sample of your prostate). This was because of a raised prostate specific antigen (PSA) result on a blood test.

The doctor has the biopsy test results and says:
"We found a small focus of **low-risk prostate neoplasm**."

After giving you these test results, the doctor explains that there are four options. These are:

Option 1. PSA monitoring: where you keep visiting the doctor to get check-ups and tests. You will be monitored at regular points in time with PSA blood tests. This is to monitor the way the low-risk prostate neoplasm behaves. If it shows signs of growing, then treatment can be started.

Option 2. Active surveillance: where you keep visiting the doctor to get check-ups and tests. You will be monitored at regular points in time with PSA blood tests, MRI scans, and prostate biopsies. This is to monitor the way the low-risk prostate neoplasm behaves. If it shows signs of growing, then treatment can be started.

Option 3. Radical prostatectomy: where you have a surgical procedure to

remove the prostate. This includes the low-risk prostate neoplasm.

Option 4. Radiotherapy: where you have a non-surgical procedure on the prostate. The prostate is treated with radiation to destroy the low-risk prostate neoplasm.

Studies have reported:

- For every 100 men found to have a low-risk prostate neoplasm, 2 will die from this over the next 15 years, regardless of the treatment.

Different treatments have different advantages and disadvantages.
Please consider the diagrams on the next page for details:

Erectile dysfunction
(difficulty getting and keeping an erection)

PSA Monitoring

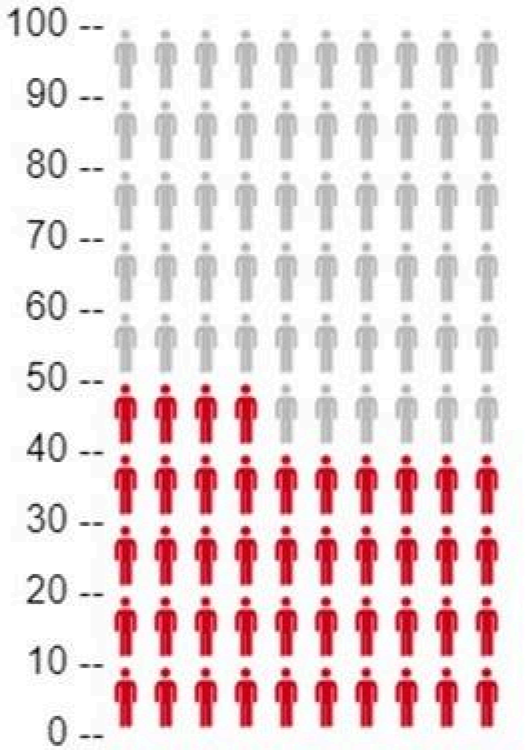

44 out of 100 men WILL NOT have erections firm enough for intercourse after 2 years.

56 out of 100 men WILL have erections firm enough for intercourse after 2 years.

Active Surveillance

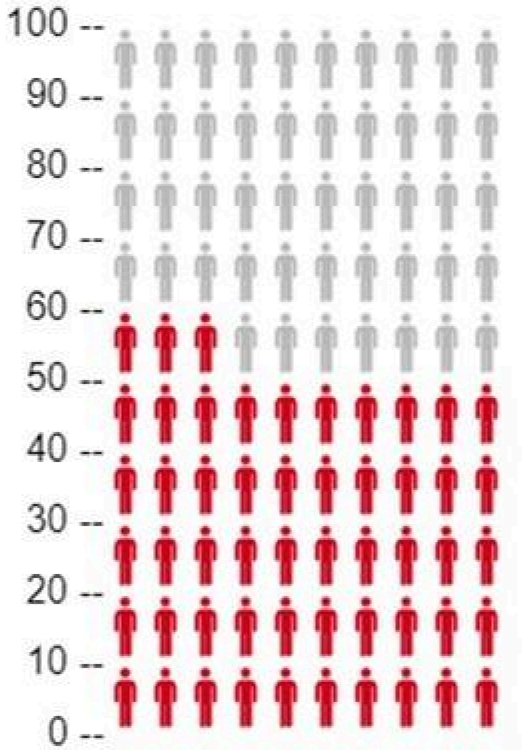

53 out of 100 men WILL NOT have erections firm enough for intercourse after 2 years.

47 out of 100 men WILL have erections firm enough for intercourse after 2 years.

Prostatectomy

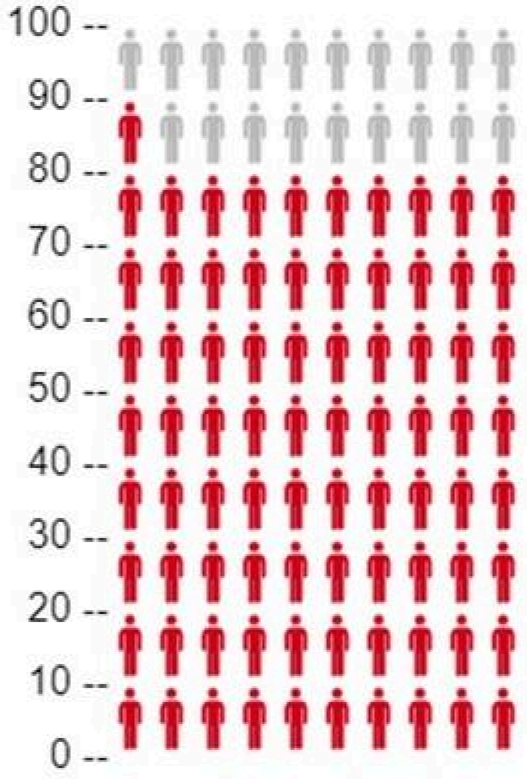

81 out of 100 men WILL NOT have erections firm enough for intercourse after 2 years.

19 out of 100 men WILL have erections firm enough for intercourse after 2 years.

Radiotherapy

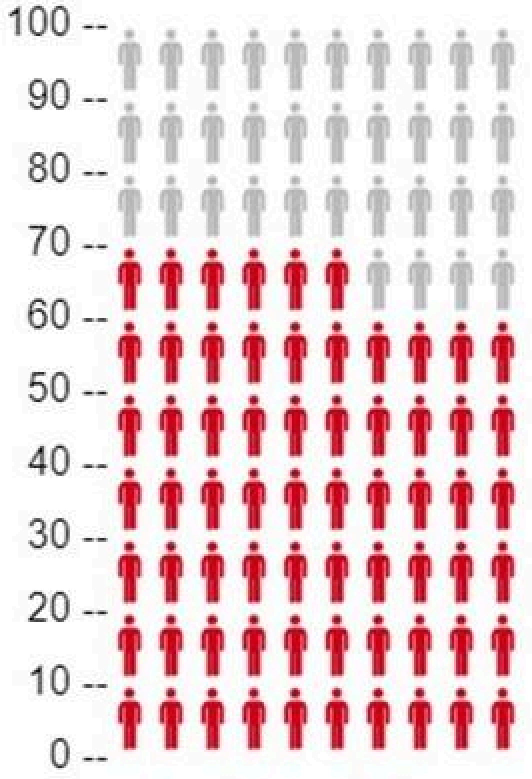

66 out of 100 men WILL NOT have erections firm enough for intercourse after 2 years.

34 out of 100 men WILL have erections firm enough for intercourse after 2 years.

Bladder control

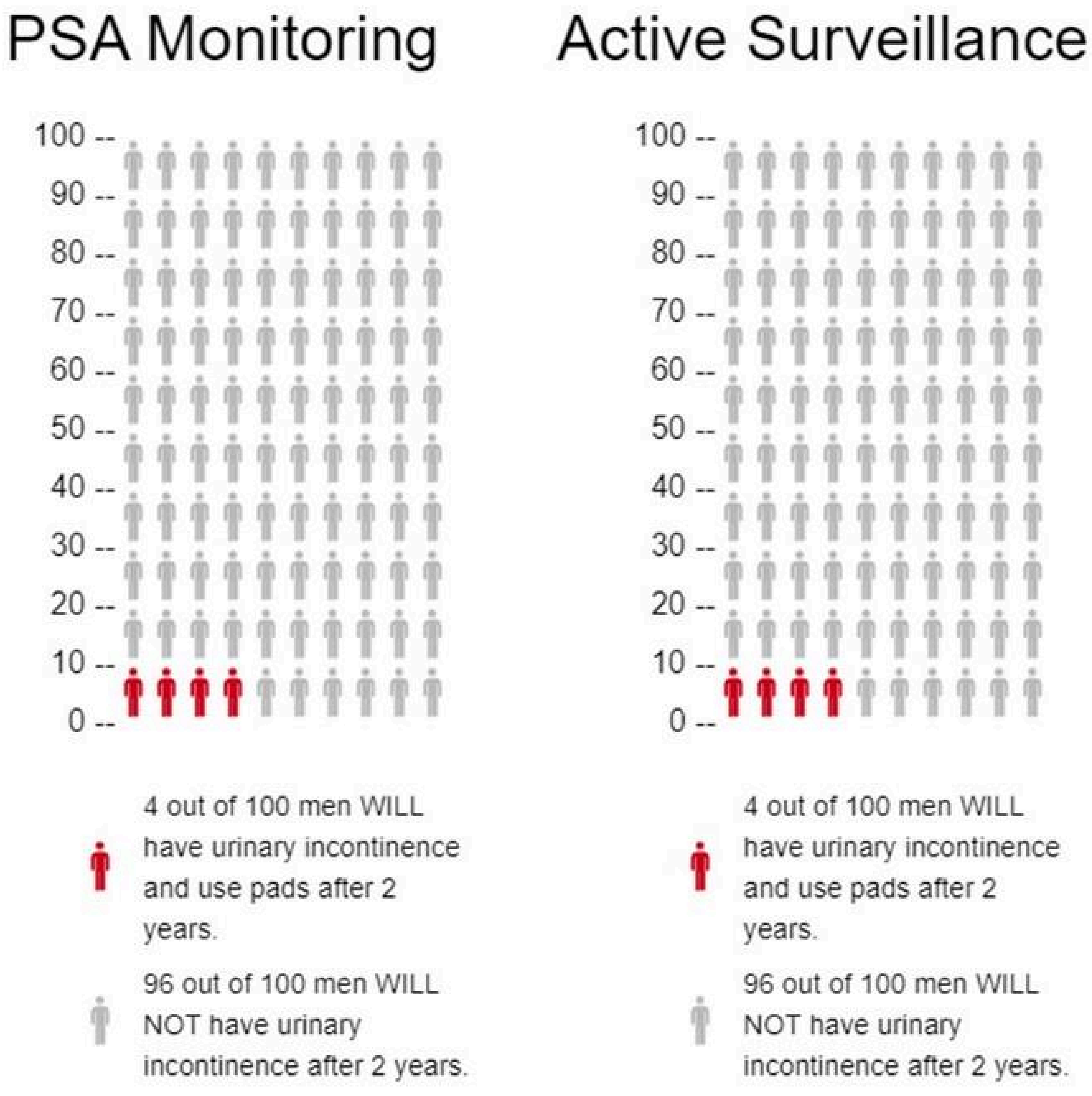

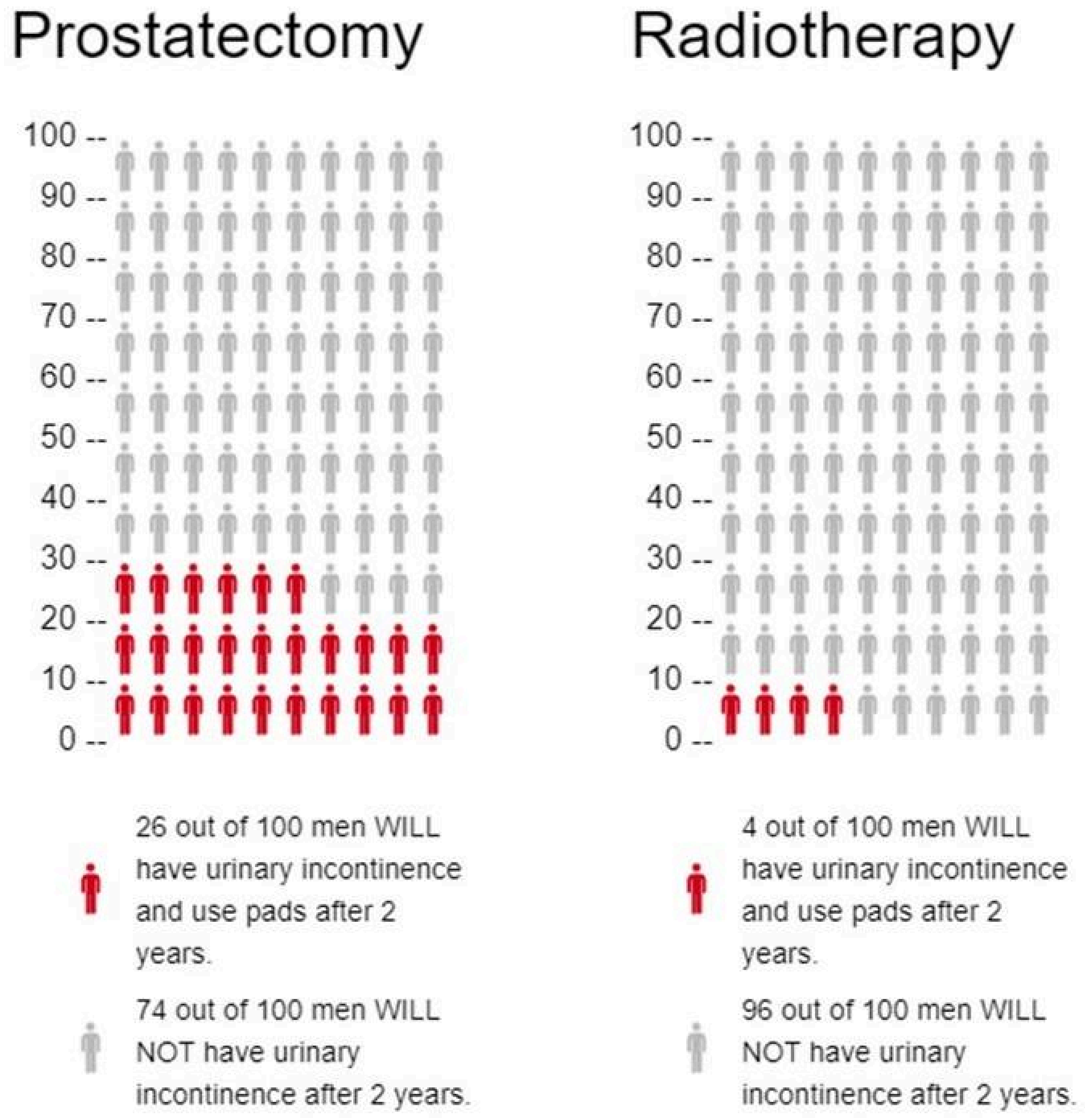
Prostatectomy
Radiotherapy
100 --
90 --
80 --
70 --
60 --
50 --
40 --
30 --
20 --
10 --
0 --
26 out of 100 men WILL have urinary incontinence and use pads after 2 years.
74 out of 100 men WILL NOT have urinary incontinence after 2 years.
4 out of 100 men WILL have urinary incontinence and use pads after 2 years.
96 out of 100 men WILL NOT have urinary incontinence after 2 years.

Bowel control

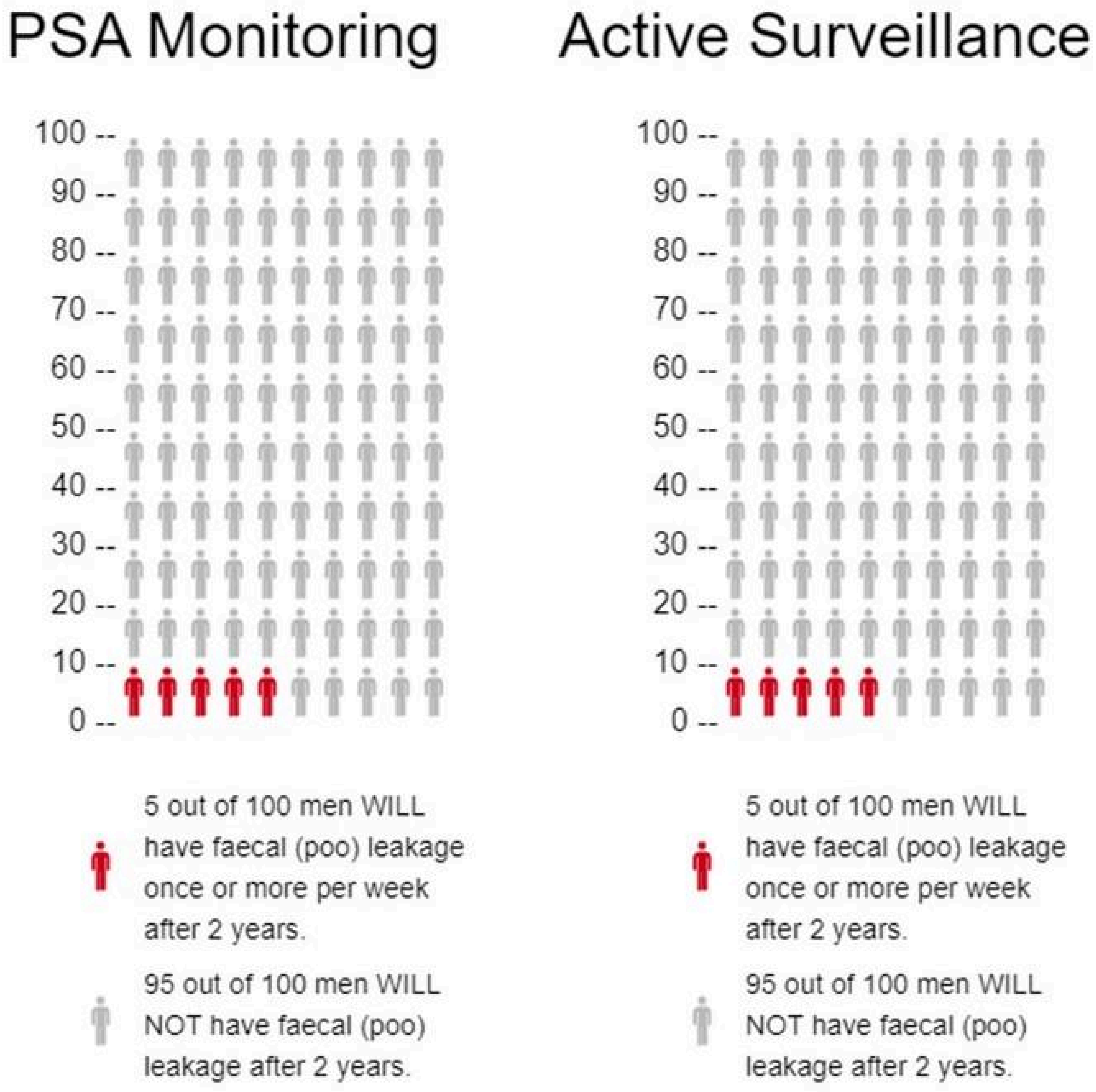

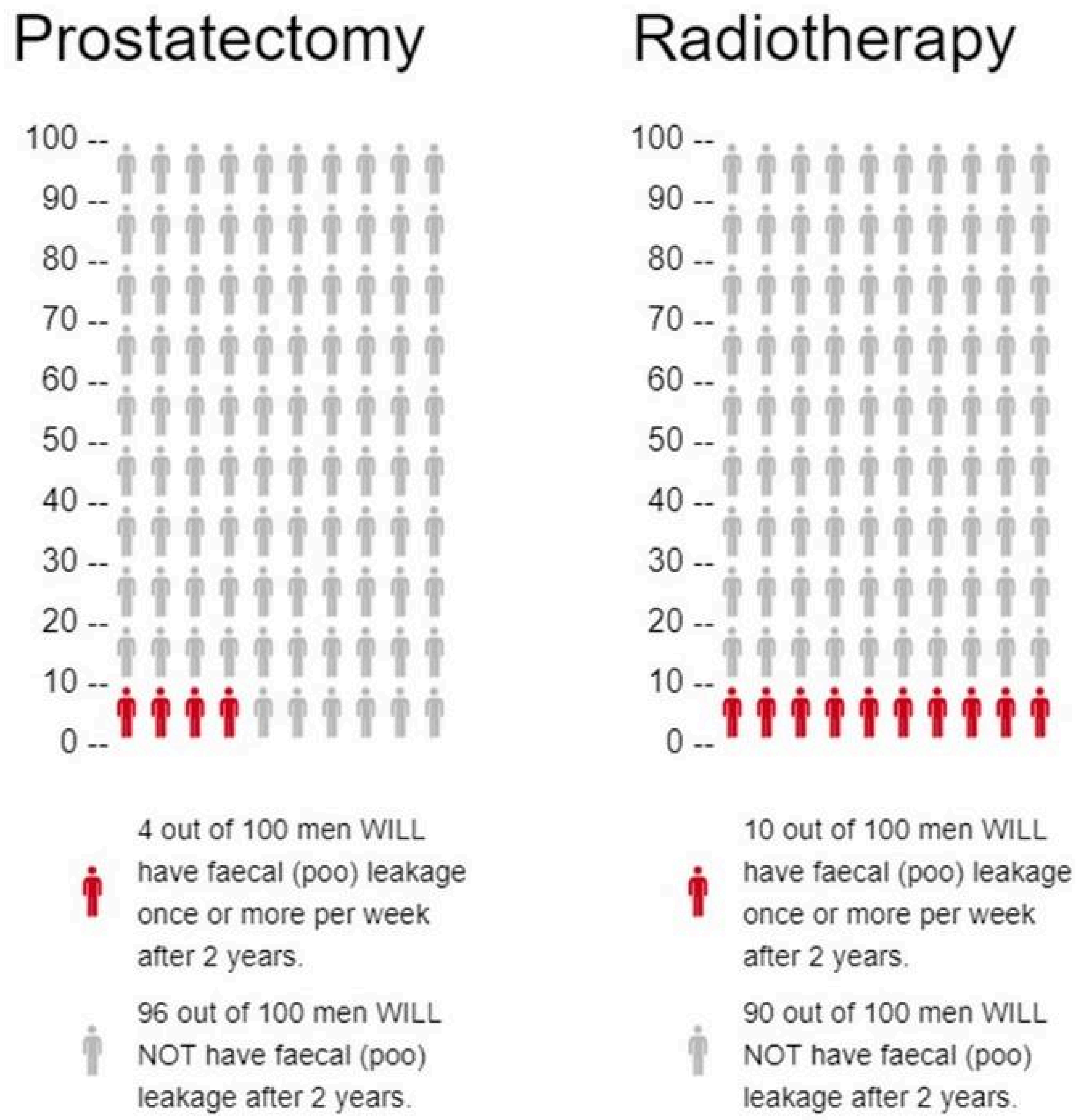
Prostatectomy
100 --
90 --
80 --
70 --
60 --
50 --
40 --
30 --
20 --
10 --
0 --
4 out of 100 men WILL have faecal (poo) leakage once or more per week after 2 years.
96 out of 100 men WILL NOT have faecal (poo) leakage after 2 years.
Radiotherapy
100 --
90 --
80 --
70 --
60 --
50 --
40 --
30 --
20 --
10 --
0 --
10 out of 100 men WILL have faecal (poo) leakage once or more per week after 2 years.
90 out of 100 men WILL NOT have faecal (poo) leakage after 2 years.

Spread

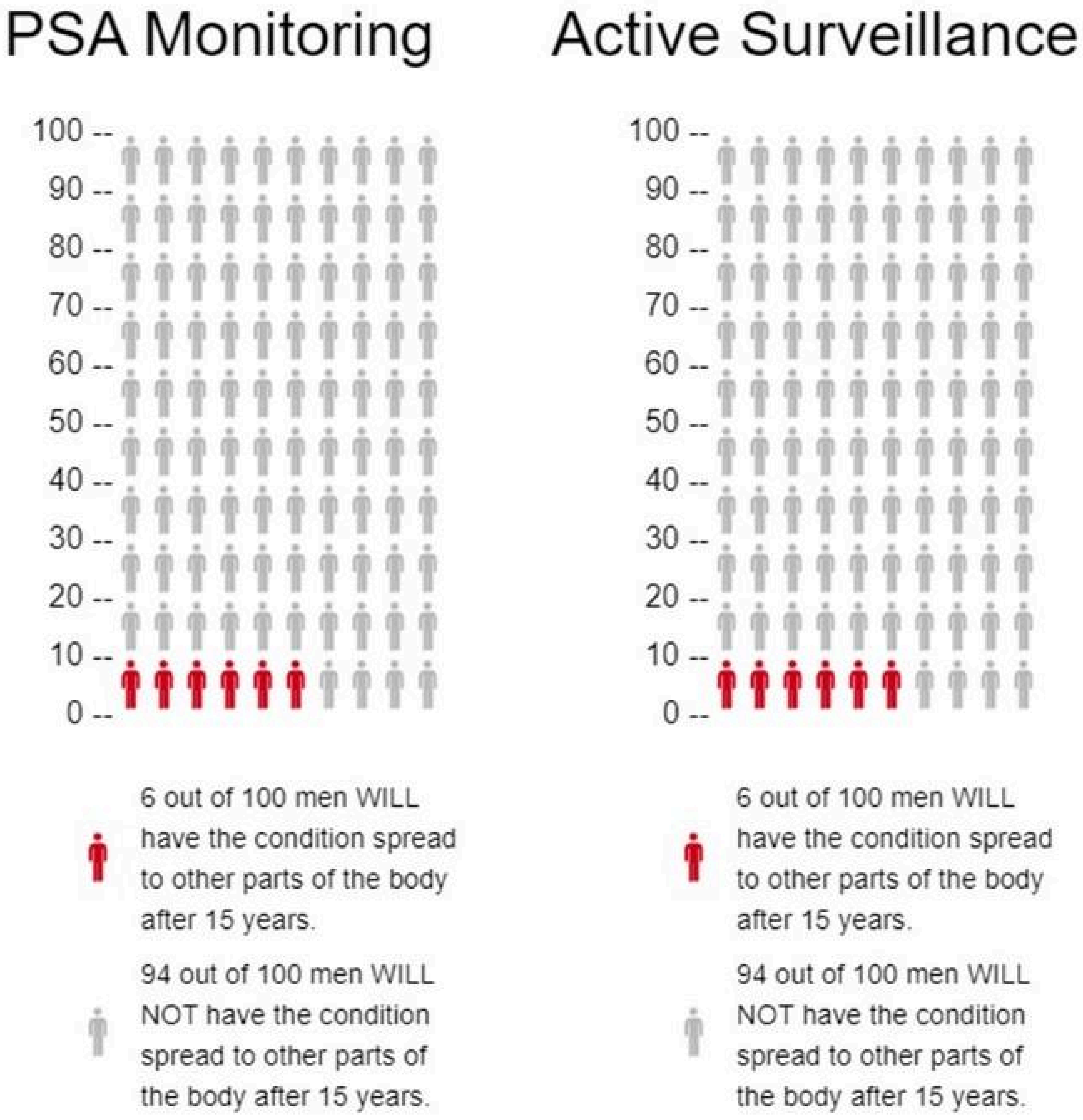
PSA Monitoring
Active Surveillance
100 --
90 --
80 --
70 --
60 --
50 --
40 --
30 --
20 --
10 --
0 --
6 out of 100 men WILL have the condition spread to other parts of the body after 15 years.
94 out of 100 men WILL NOT have the condition spread to other parts of the body after 15 years.
6 out of 100 men WILL have the condition spread to other parts of the body after 15 years.
94 out of 100 men WILL NOT have the condition spread to other parts of the body after 15 years.

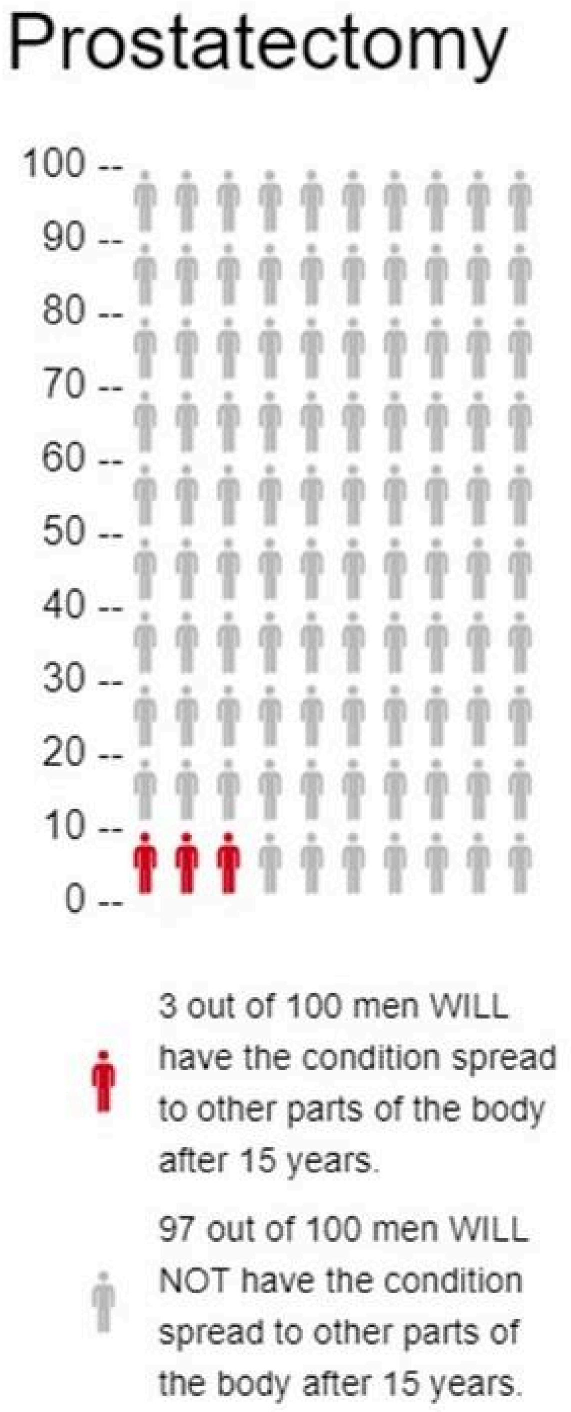

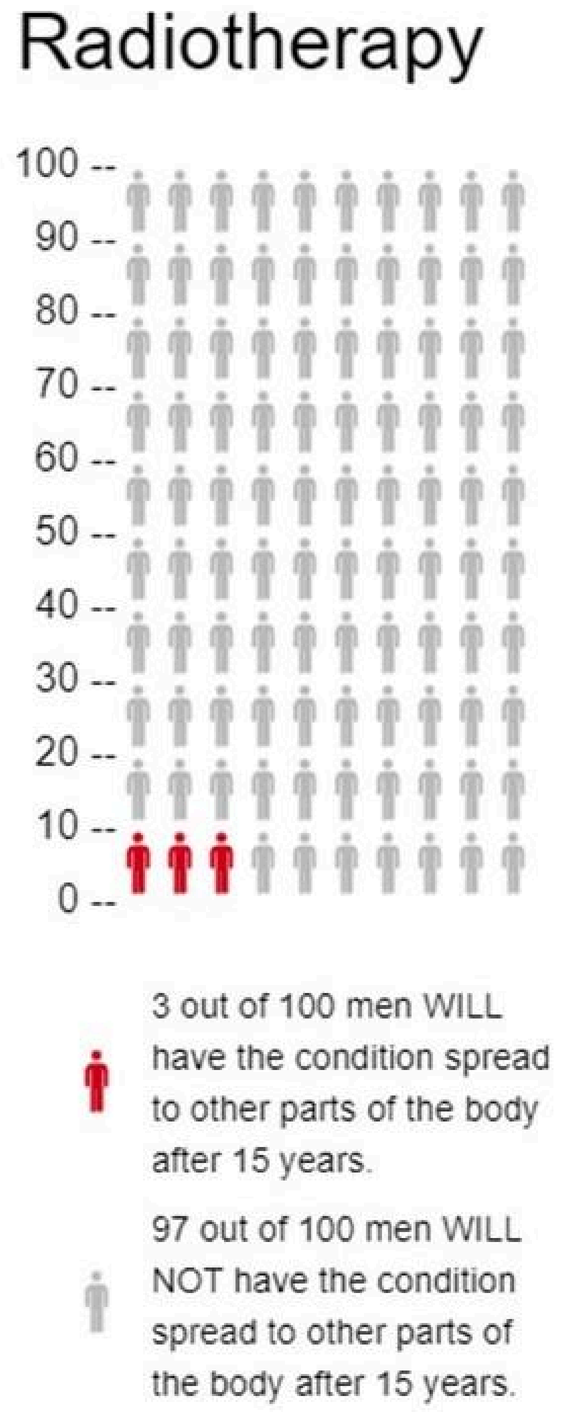

Hypothetical - Label 2 + Low Information - Men

Please read the information below and answer the questions that follow. You are asked to imagine as if the following scenario is true. Please answer how you would feel or react if you were in this situation, to the best of your ability.

You are at the doctor (GP) after you recently had a prostate biopsy (sample of your prostate). This was because of a raised prostate specific antigen (PSA) result on a blood test.

The doctor has the biopsy test results and says:
“We found a small focus of **low-risk prostate lesion**.”

After giving you these test results, the doctor explains that there are four options. These are:

Option 1. PSA monitoring: where you keep visiting the doctor to get check-ups and tests. You will be monitored at regular points in time with PSA blood tests. This is to monitor the way the low-risk prostate lesion behaves. If it shows signs of growing, then treatment can be started.

Option 2. Active surveillance: where you keep visiting the doctor to get check-ups and tests. You will be monitored at regular points in time with PSA blood tests, MRI scans, and prostate biopsies. This is to monitor the way the low-risk prostate lesion behaves. If it shows signs of growing, then treatment can be started.

Option 3. Radical prostatectomy: where you have a surgical procedure to remove the prostate. This includes the low-risk prostate lesion.

Option 4. Radiotherapy: where you have a non-surgical procedure on the prostate. The prostate is treated with radiation to destroy the low-risk prostate lesion.

Studies have reported:

- For every 100 men found to have a low-risk prostate lesion, 2 will die from this over the next 15 years, regardless of the treatment.

Different treatments have different advantages and disadvantages.

Hypothetical - Label 2 + High Information - Men

Please read the information below and answer the questions that follow. You are asked to imagine as if the following scenario is true. Please answer how you would feel or react if you were in this situation, to the best of your ability.

You are at the doctor (GP) after you recently had a prostate biopsy (sample of your prostate). This was because of a raised prostate specific antigen (PSA) result on a blood test.

The doctor has the biopsy test results and says:
"We found a small focus of **low-risk prostate lesion**."

After giving you these test results, the doctor explains that there are four options. These are:

Option 1. PSA monitoring: where you keep visiting the doctor to get check-ups and tests. You will be monitored at regular points in time with PSA blood tests. This is to monitor the way the low-risk prostate lesion behaves. If it shows signs of growing, then treatment can be started.

Option 2. Active surveillance: where you keep visiting the doctor to get check-ups and tests. You will be monitored at regular points in time with PSA blood tests, MRI scans, and prostate biopsies. This is to monitor the way the low-risk prostate lesion behaves. If it shows signs of growing, then treatment can be started.

Option 3. Radical prostatectomy: where you have a surgical procedure to

remove the prostate. This includes the low-risk prostate lesion.

Option 4. Radiotherapy: where you have a non-surgical procedure on the prostate. The prostate is treated with radiation to destroy the low-risk prostate lesion.

Studies have reported:

- For every 100 men found to have a low-risk prostate lesion, 2 will die from this over the next 15 years, regardless of the treatment.

Different treatments have different advantages and disadvantages.
Please consider the diagrams on the next page for details:

Erectile dysfunction
(difficulty getting and keeping an erection)

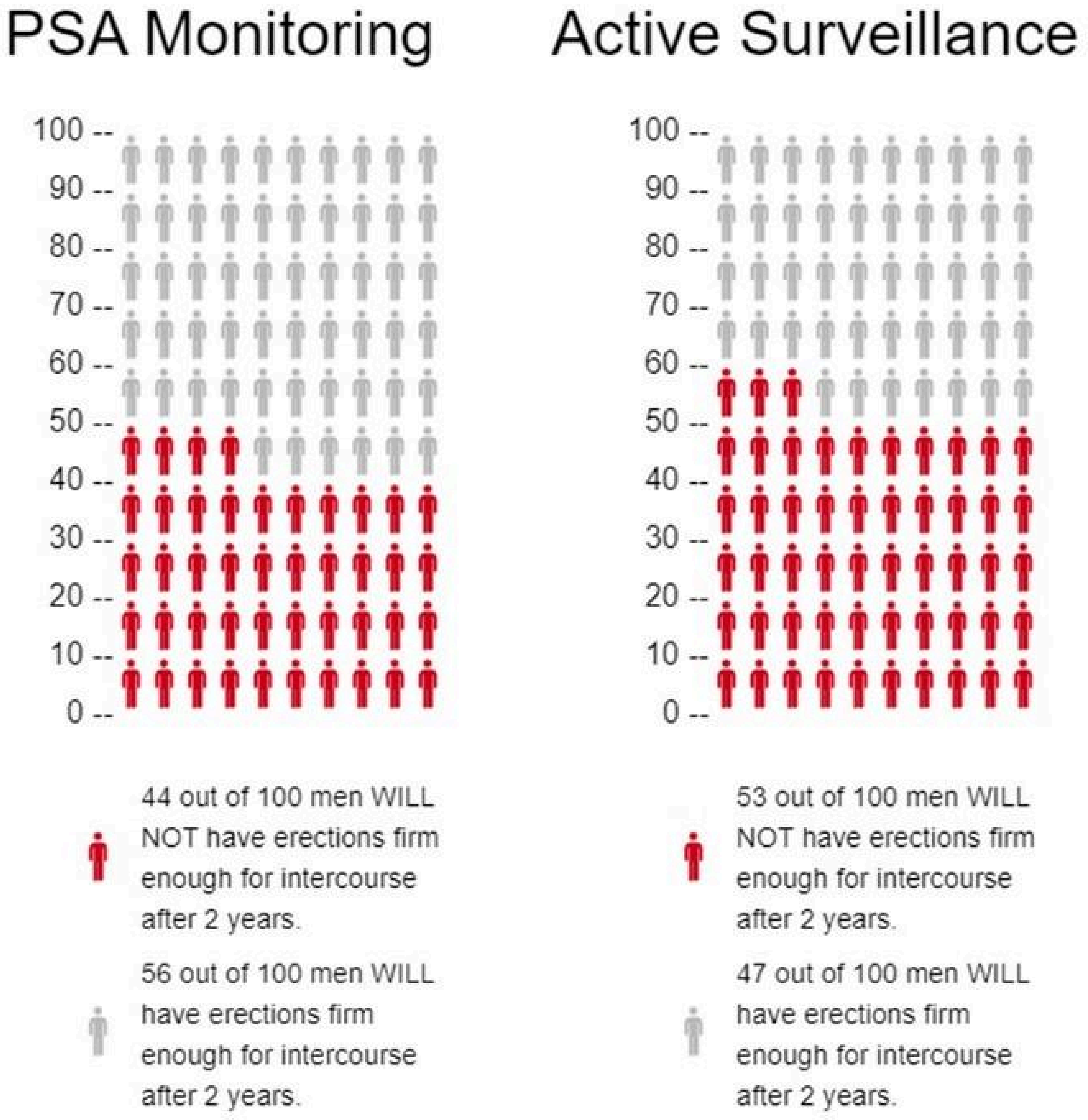
PSA Monitoring
Active Surveillance
100 --
90 --
80 --
70 --
60 --
50 --
40 --
30 --
20 --
10 --
0 --
44 out of 100 men WILL NOT have erections firm enough for intercourse after 2 years.
56 out of 100 men WILL have erections firm enough for intercourse after 2 years.
53 out of 100 men WILL NOT have erections firm enough for intercourse after 2 years.
47 out of 100 men WILL have erections firm enough for intercourse after 2 years.

Prostatectomy

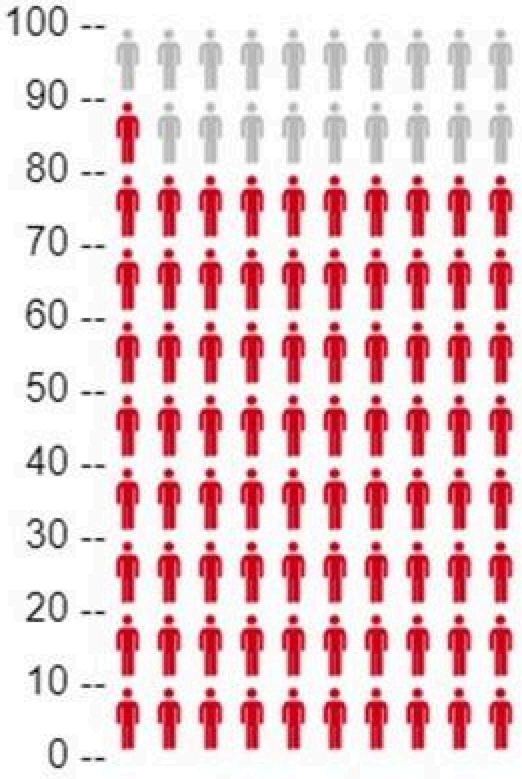

81 out of 100 men WILL NOT have erections firm enough for intercourse after 2 years.

19 out of 100 men WILL have erections firm enough for intercourse after 2 years.

Radiotherapy

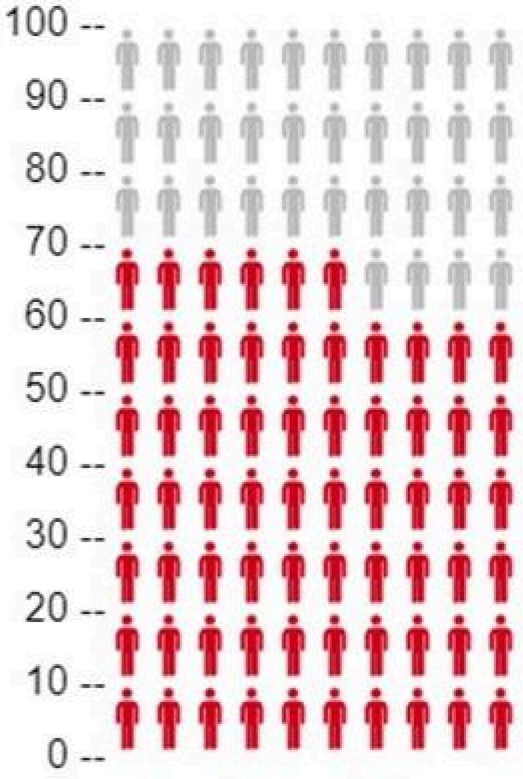

66 out of 100 men WILL NOT have erections firm enough for intercourse after 2 years.

34 out of 100 men WILL have erections firm enough for intercourse after 2 years.

Bladder control

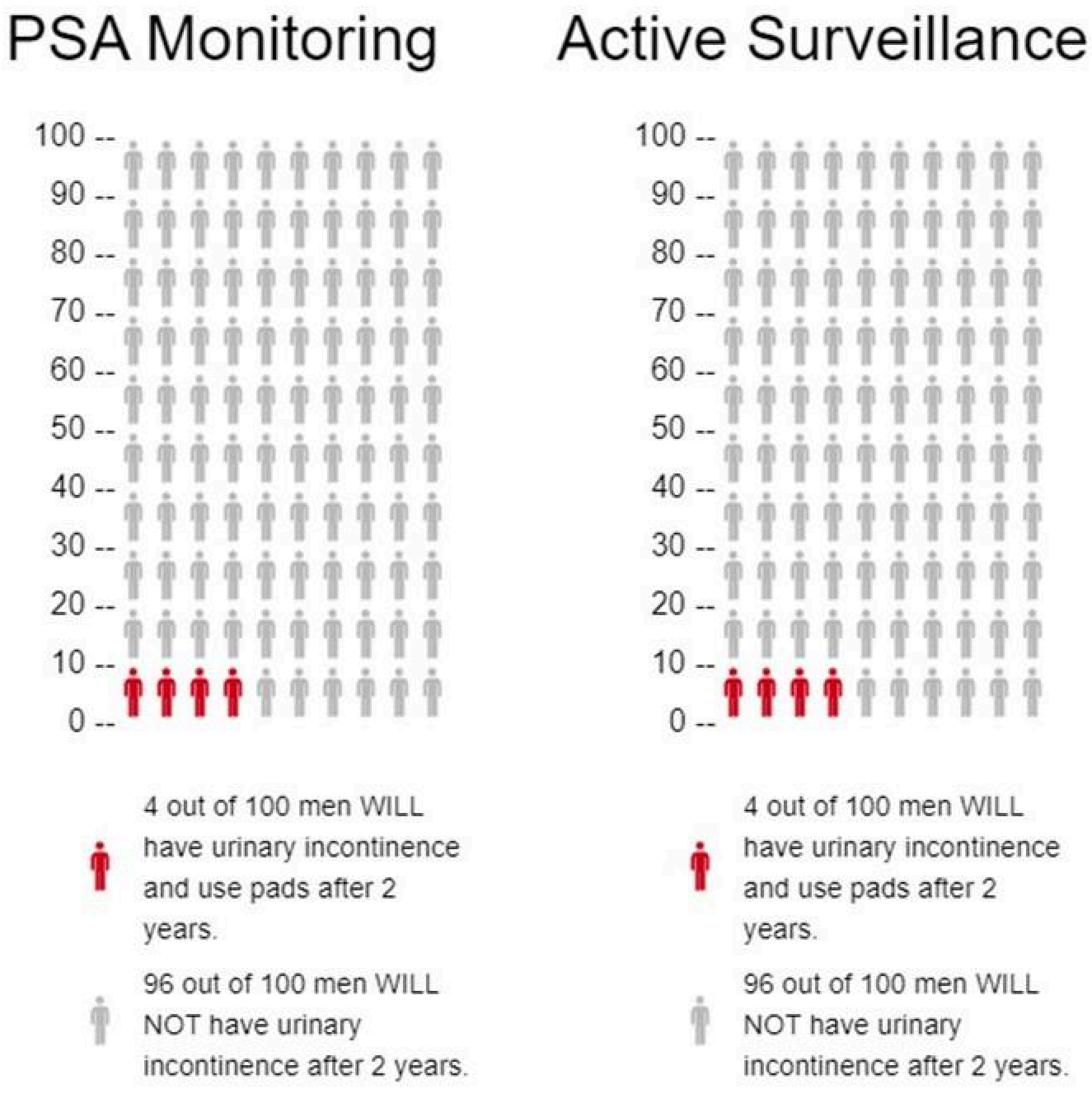

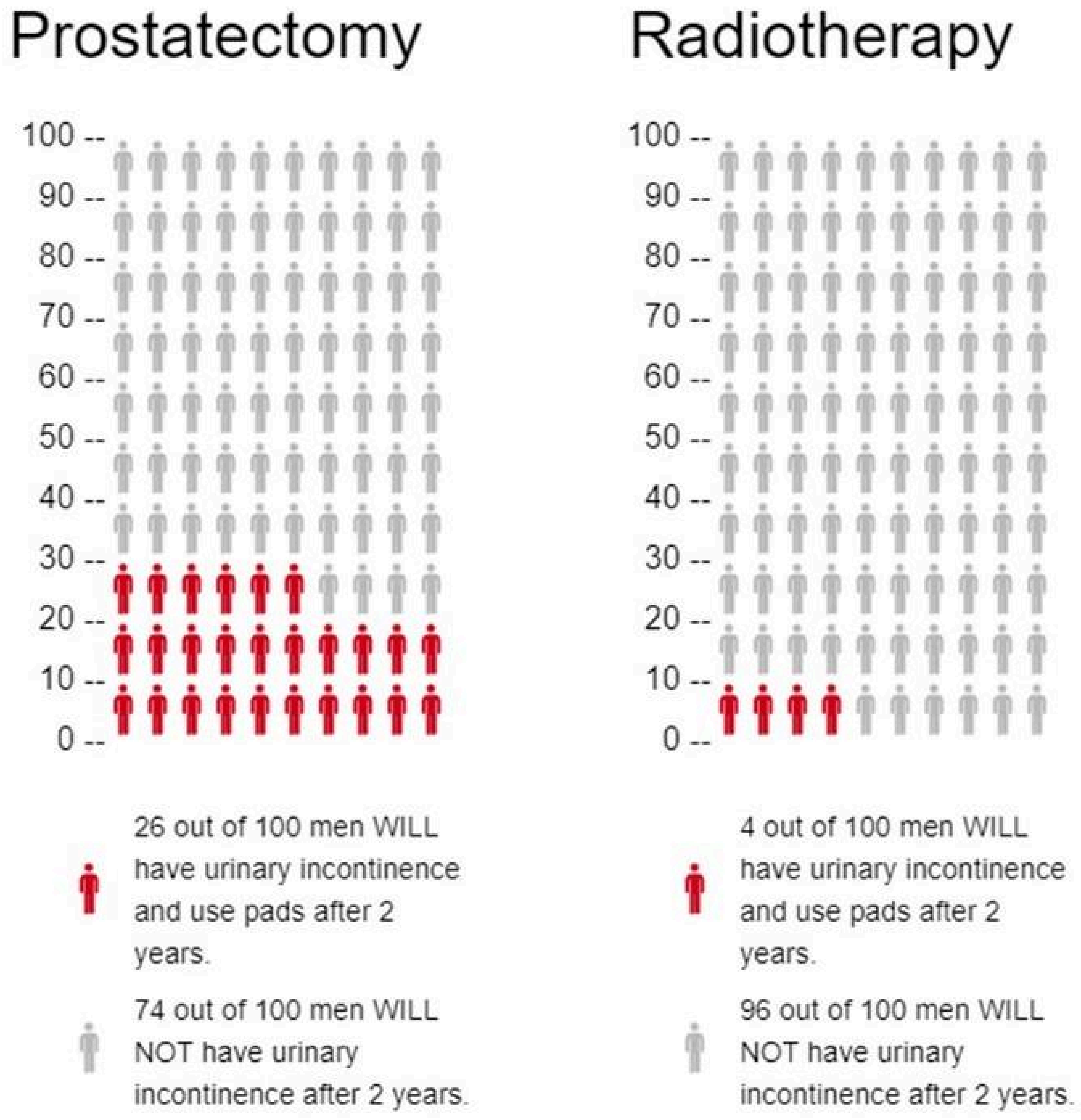
Prostatectomy
Radiotherapy
100 --
90 --
80 --
70 --
60 --
50 --
40 --
30 --
20 --
10 --
0 --
26 out of 100 men WILL have urinary incontinence and use pads after 2 years.
74 out of 100 men WILL NOT have urinary incontinence after 2 years.
4 out of 100 men WILL have urinary incontinence and use pads after 2 years.
96 out of 100 men WILL NOT have urinary incontinence after 2 years.

Bowel control

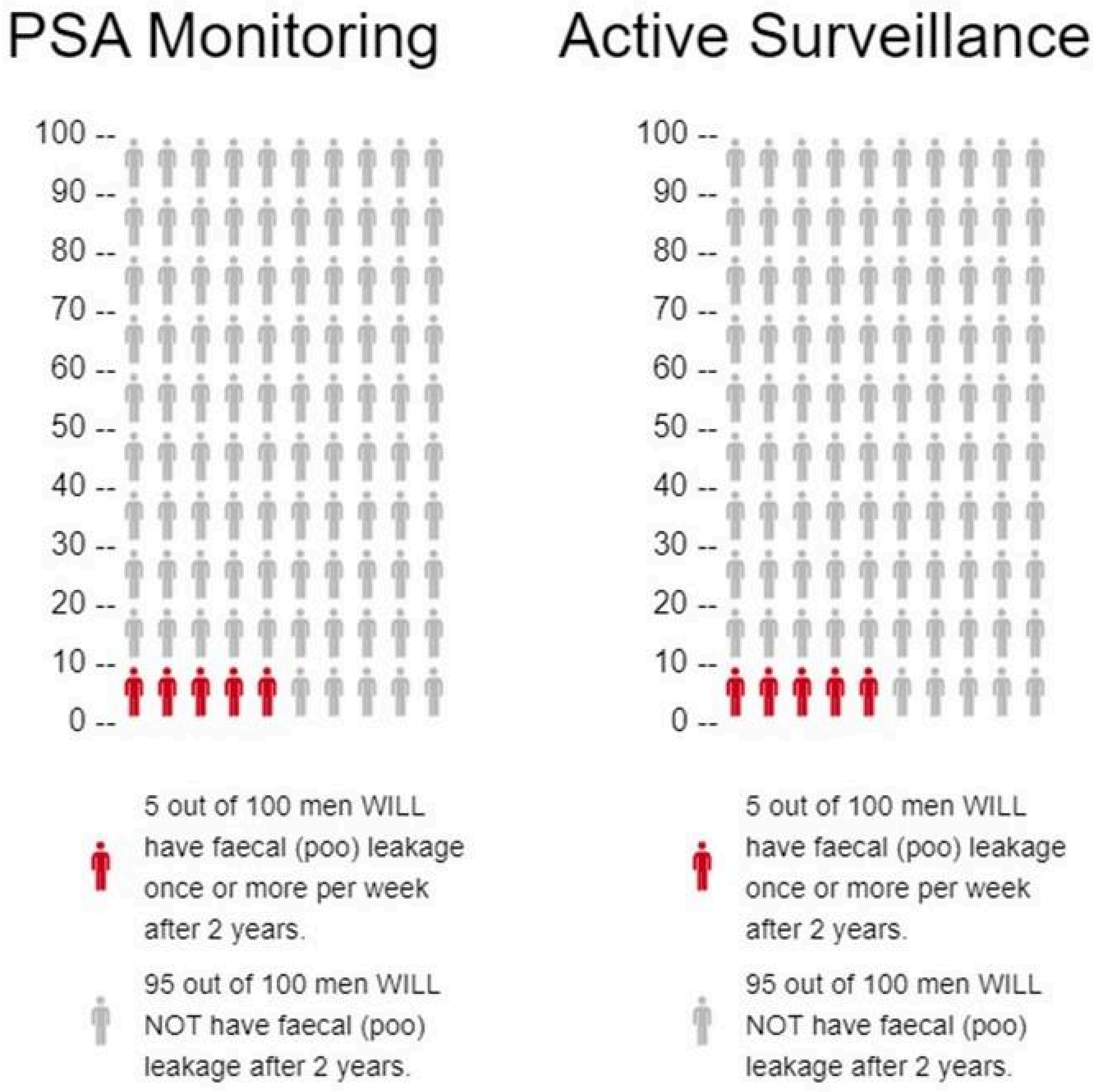

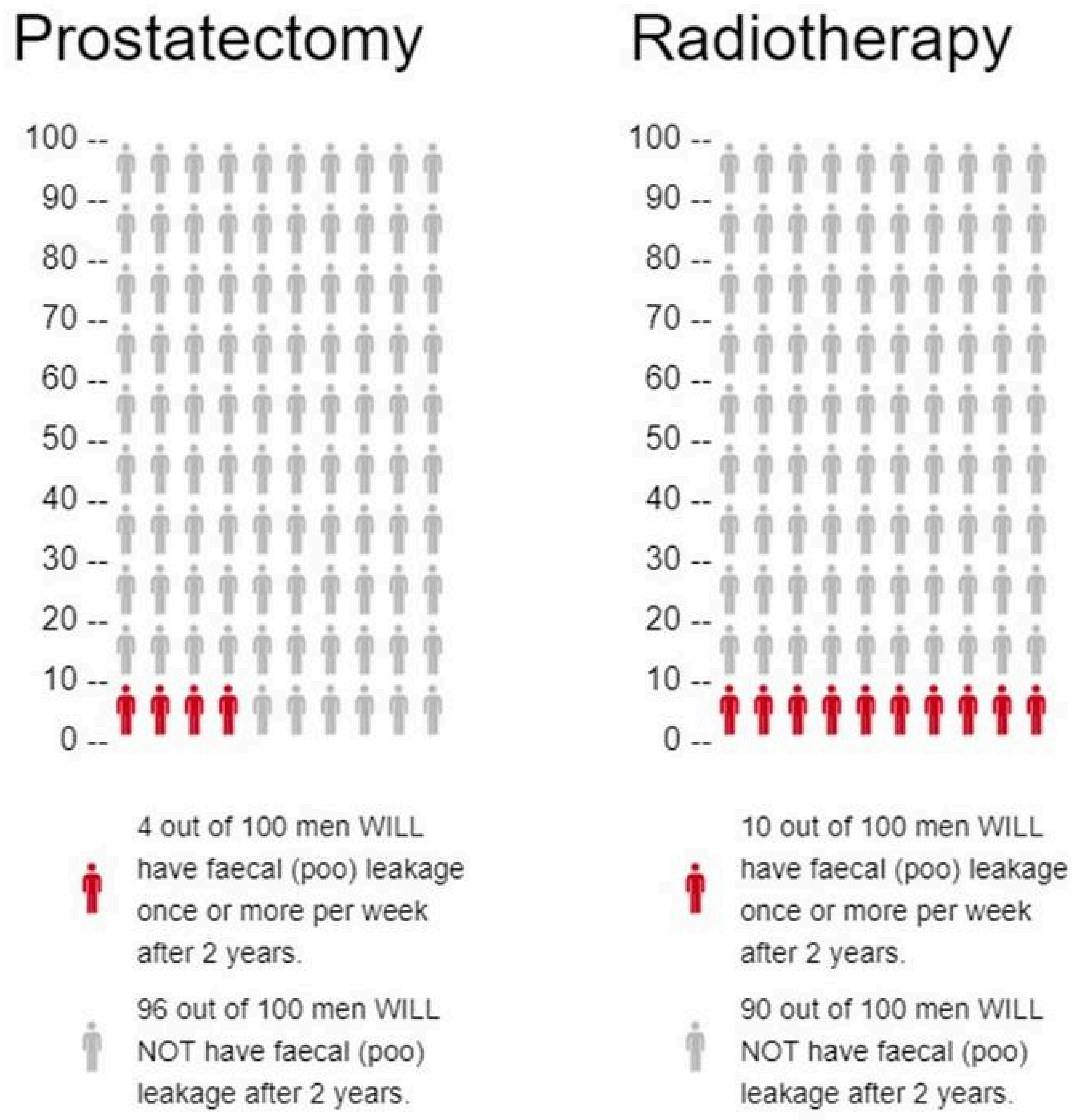
Prostatectomy
Radiotherapy
100 --
90 --
80 --
70 --
60 --
50 --
40 --
30 --
20 --
10 --
0 --
4 out of 100 men WILL have faecal (poo) leakage once or more per week after 2 years.
96 out of 100 men WILL NOT have faecal (poo) leakage after 2 years.
10 out of 100 men WILL have faecal (poo) leakage once or more per week after 2 years.
90 out of 100 men WILL NOT have faecal (poo) leakage after 2 years.

Spread

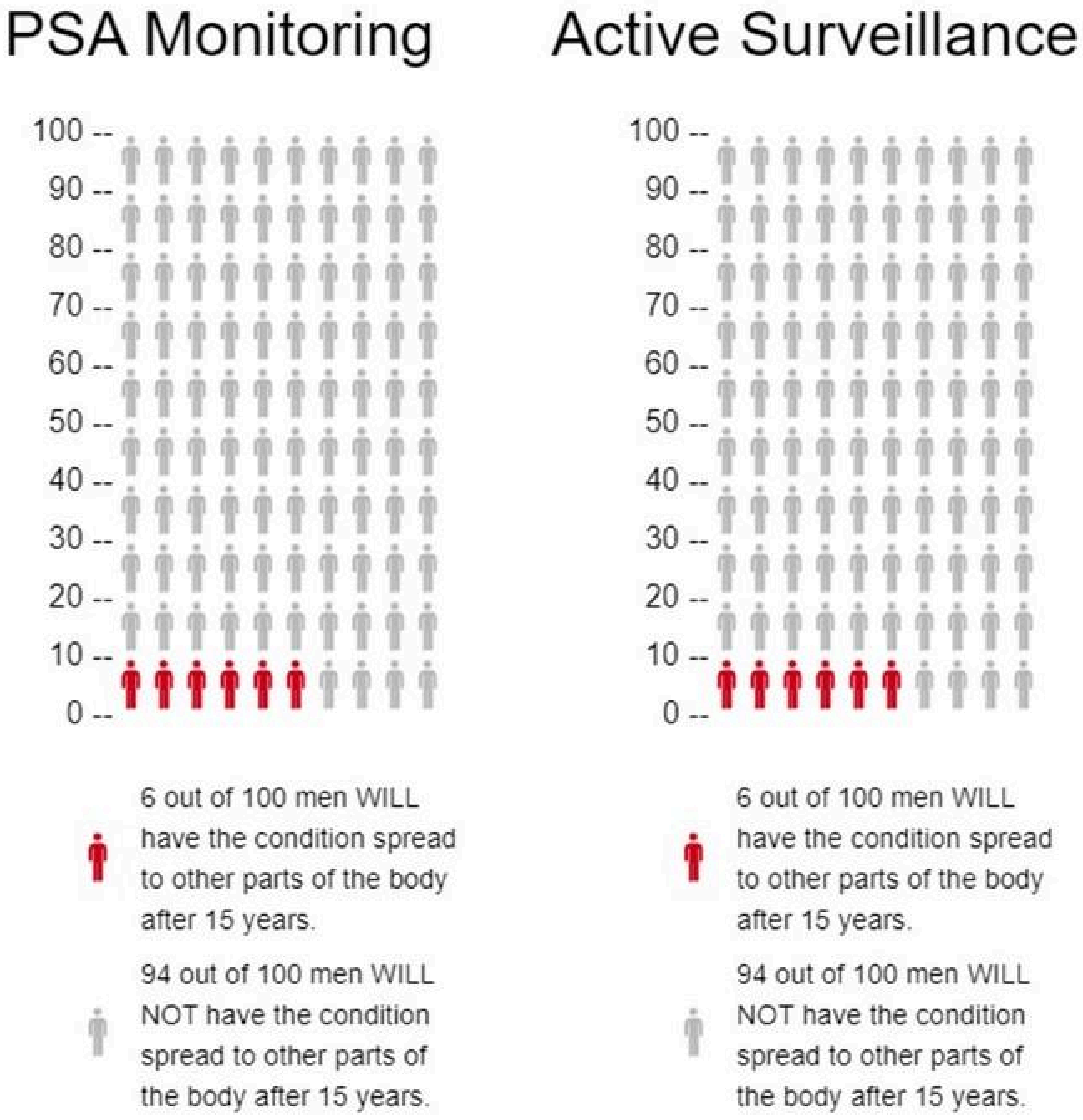
PSA Monitoring
Active Surveillance
100 --
90 --
80 --
70 --
60 --
50 --
40 --
30 --
20 --
10 --
0 --
6 out of 100 men WILL have the condition spread to other parts of the body after 15 years.
94 out of 100 men WILL NOT have the condition spread to other parts of the body after 15 years.
6 out of 100 men WILL have the condition spread to other parts of the body after 15 years.
94 out of 100 men WILL NOT have the condition spread to other parts of the body after 15 years.

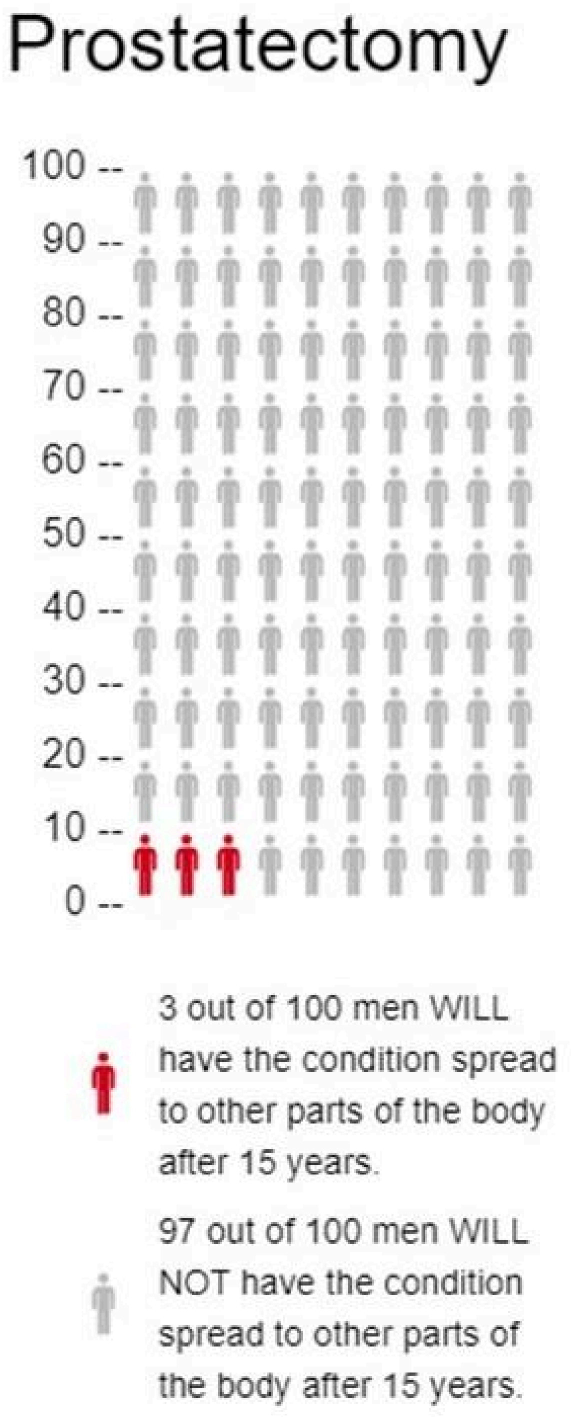

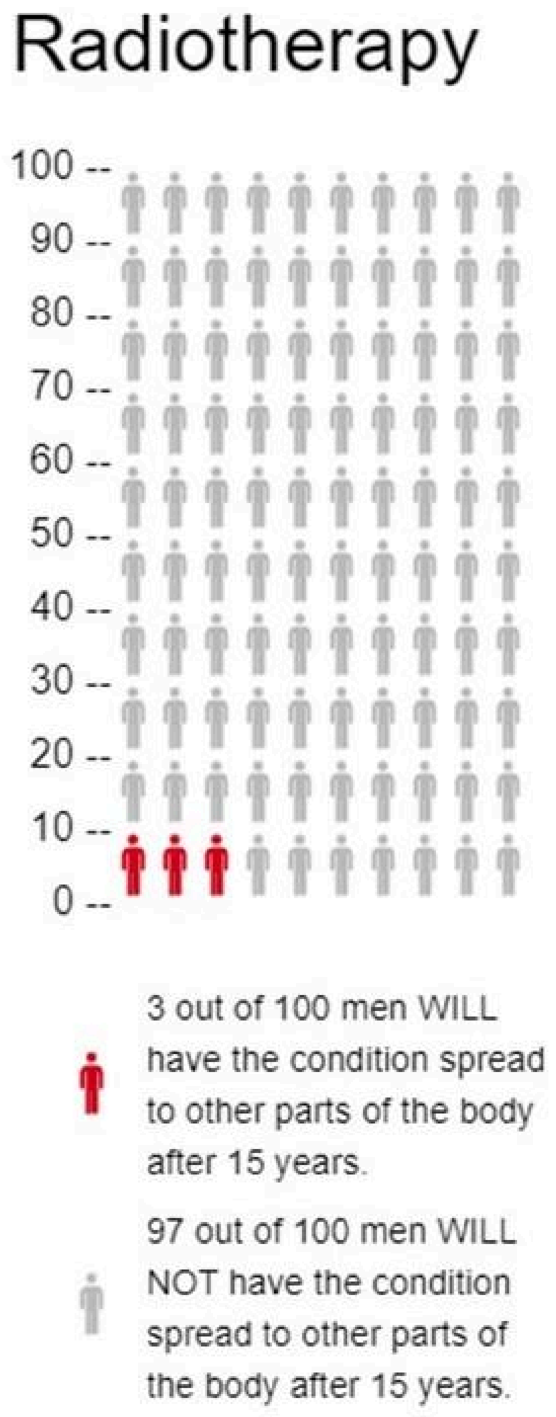

Section 5: Primary and Secondary Outcome Measures ✓

After learning of this pathology result, how anxious do you feel?

Not anxious at all	Extremely anxious
0	10

After learning of your pathology result, which of these management options would you choose?

- ◯ **1. PSA monitoring:** where you keep visiting your doctor to get check-ups and tests. You will be monitored at regular points in time with PSA blood tests.
- ◯ **2. Active surveillance:** where you keep visiting the doctor to get check-ups and tests. You will be monitored at regular points in time with PSA blood tests, MRI scans, and prostate biopsies.
- ◯ **3. Prostatectomy:** where you have a surgical procedure to remove the prostate.
- ◯ **4. Radiotherapy:** where you have a non-surgical procedure on the prostate.

After learning of your partner's pathology result, which of these management options would you advise them to use?

- ◯ **1. PSA monitoring:** where your partner keeps visiting the doctor to get check-ups and tests. Your partner will be monitored at regular points in time with PSA blood tests.
- ◯ **2. Active surveillance:** where your partner keeps visiting the doctor to get check-ups and tests. Your partner will be monitored at regular points in time with PSA blood tests, MRI scans, and prostate biopsies.
- ◯ **3. Prostatectomy:** where your partner has a surgical procedure to remove the prostate.
- ◯ **4. Radiotherapy:** where your partner has a non-surgical procedure on the prostate.

Please tell us how you decided on that management option. What were the important factors that helped you decide? [This question is optional].

After making that management choice, how anxious do you feel?

	**Not anxious at all**	**Extremely anxious**
	0	10

Imagine it is five years after your initial diagnosis and you are seeing the doctor for a follow up clinic visit. You have had regular check ups and tests for your prostate each year. The results have all been stable – nothing has changed. How likely are you to now choose treatment (prostatectomy or radiotherapy) instead of PSA monitoring or active surveillance for your prostate?

Very unlikely	Somewhat unlikely	Neither likely nor unlikely	Somewhat likely	Very likely
○	○	○	○	○

Imagine it is five years after the initial diagnosis and your partner is seeing the doctor for a follow up clinic visit. Your partner has had regular check ups and tests for his prostate each year. The results have all been stable – nothing has changed. How likely are you to now recommend treatment (prostatectomy or radiotherapy) instead of PSA monitoring or active surveillance for your partner?

Very unlikely	Somewhat unlikely	Neither likely nor unlikely	Somewhat likely	Very likely
○	○	○	○	○

Section 6: Debrief Statement ✓

Thank you - you have now completed the survey.

You were a participant in this study which aimed to investigate how people would react to different information provision on diagnosis of a low-risk prostate lesion results by the label given to the prostate lesion.

During the study, you were asked to imagine a hypothetical scenario in which you or your partner are given a diagnosis result after having gone to a routine screening. You were then asked to complete a series of survey questions.

You were randomised to receive one of three different hypothetical scenarios.

These three diagnosis scenarios were:

1. Diagnosis of low-risk prostate cancer, grade group 1.
2. Diagnosis of low-risk prostate neoplasm
3. Diagnosis of low-risk prostate lesion.

The purpose of this study was to examine the impact of these different labels/diagnoses on preferred management strategy and psychological outcomes such as worry and health seeking intentions.

It is important to remember that this study was entirely hypothetical (made up). The study team does not have access to any of your medical history.

If you have any further questions regarding the study, feel free to contact the researchers: Prof Katy Bell (katy.bell@sydney.edu.au) or Dr Brooke Nickel (brooke.nickel@sydney.edu.au).

For more information on prostate cancer and screening processes, please visit the following websites:

Health Direct – Prostate Cancer
Cancer Council – Prostate Cancer

[Prostate Cancer Foundation of Australia]
[Understanding Active Surveillance - PCFA]
[PSA Testing Guidelines - PCFA]
[Active Surveillance for Prostate Cancer – Cancer Council NSW]

Section 7: Feedback ✓

Thank you for your participation in the survey. Your time and contribution is greatly appreciated. If you are interested in the results of the study, the published report and a lay summary of the results will be published at the following web page: https://www.wiserhealthcare.org.au/category/publications/

Powered by Qualtrics
