# Supplementary material for: Impact of the diagnostic label for a low-risk prostate lesion: protocol for two online factorial randomised experiments
Source: BMJ Open. 2024 Aug 9;14(8):e085947. doi: 10.1136/bmjopen-2024-085947 (PMC11331948; doi:10.1136/bmjopen-2024-085947)
Supplement: Supplementary file 2 [file bmjopen-2024-085947.reviewer_comments.pdf]

PEER REVIEW HISTORY

BMJ Open publishes all reviews undertaken for accepted manuscripts. Reviewers are asked to complete a checklist review form and are provided with free text boxes to elaborate on their assessment. These free text comments are reproduced below.

ARTICLE DETAILS

**TITLE (PROVISIONAL)**	Impact of the diagnostic label for a low-risk prostate lesion: protocol for two online factorial randomised experiments
**AUTHORS**	Bullen, James; Nickel, Brooke; McCaffery, Kirsten; Wilt, Timothy; Smith, Jenna; Boroumand, Farzaneh; Parker, Lisa; Millar, Jeremy; Brodersen, John; Dahm, Philipp; Delahunt, Brett; Varma, Murali; Glasziou, Paul; Warden, Andrew; Diller, Lawrence; Billington, Larry; van Rensburg, Christo; Bell, Katy

VERSION 1 - REVIEW

**REVIEWER NAME**	Mai, Zhipeng
**REVIEWER AFFILIATION**	Peking Union Medical College Hospital
**REVIEWER CONFLICT OF INTEREST**	I have no potentially conflicting or competing interests with the manuscript or its authors.
**DATE REVIEW RETURNED**	18-Mar-2024

**GENERAL COMMENTS**	This study delves into the impact of different diagnostic labels for low-risk prostate cancer on management choice and anxiety level among patients and their families. Overall, the study protocol exhibits innovative design, elucidates clear research concepts, presents comprehensive plans, and is both reasonable and feasible in its approach.

**REVIEWER NAME**	Lawrenson, Ross
**REVIEWER AFFILIATION**	Hamilton
**REVIEWER CONFLICT OF INTEREST**	I have no competing interests
**DATE REVIEW RETURNED**	13-May-2024

**GENERAL COMMENTS**	Thank you for asking me to review this interesting study. 75% of prostate cancers that are diagnosed are low risk and over diagnosis is a serious problem. Consequently, the psychological impact of the diagnosis can in some cases be the major health impact on a man and his family. Ways of reducing this impact are welcome. The study has taken a co-design approach, identifying wordings that are used by Clinical Advisors and Consumers and then using a randomised trial to examine the impacts of the different terms. It is welcome to see the involvement of wives in this study as in our experience they and the wider family (especially daughters) often take the lead in helping patients with prostate cancer decide on their treatment options. The introduction outlines the background to the study. On lines 116 and 117 I was confused as whether Gleason Group reflected the prostate cancer grade group or the Gleason Score choice? This should be clarified as to which had the lower preference for treatment.

	The methods should indicate how patients are recruited and how any bias will be mitigated. The authors say “Australian community members” will be randomised. Everyone is a community member. Are/have the authors advertised for participants or invited patients from a register or other source? How have these invitations been made? Facebook, TV, radio? I am not sure what they mean by “participants will be recruited via Qualtrics? “ How have they ensured minority groups, low SES etc are included?<br>One concern I have that is not explained is the age boundaries of the study. While it is clear men under 50 are excluded, is there an upper age limit? After all the likelihood of active treatment, or active surveillance in those over 75 years of age is small. These patients have very different risk profiles with regards their risk of developing symptomatic cancer or dying from it. Also there are a diverse number of active surveillance strategies being used with increasing use of MRI. Certainly MRI in our jurisdiction is not available to all, and repeated biopsies are often off putting to patients (Ong WL). Have the authors considered stratified randomisation by age group of the cancer patients? Or by other factors e.g to ensure those whose first language is not English respond in a similar way to the different terms?<br><br>How have risk estimates been collated?? Eg 3% developing metastases in 15 years seems low (Bill-Axelson A) and may falsely alter the patient choice of treatment. The estimates seem low, especially for what would be expected for older patients. Also have the authors considered the consistency of the information to be given with what is on line? For instance if you search using the three different terms you are directed to different websites. This also depends on whether you filter your online search to Australia. After all if the different terms are used in providing information it is likely patients/family will search for these terms to better understand their condition and options. By searching “low risk prostate cancer” you are directed to a number of American sites e.g. UCLA – if au is added to the search you are directed to the Prostate Cancer Foundation of Australia. How people interpret different terms such as cancer, neoplasm or lesions is likely going to be influenced from what is available online and this point maybe something for the discussion.<br><br>Overall this seems a large well thought out study and I look forward to seeing its findings in due course.<br><br>Ong WL, Thangasamy I, Murphy D et al. Large variation in conservative management of low-risk prostate cancer in Australia and New Zealand. BJU Int. 2022 Jun; 130(Suppl 1): 17–19.<br>Bill-Axelson A, Holmberg L, Garmo H et al. Radical Prostatectomy or Watchful Waiting in Prostate Cancer — 29-Year Follow-up. N Engl J Med. 018;379:2319-2329 DOI: 10.1056/NEJMoa1807801

**REVIEWER NAME**	Lang, Eddy
**REVIEWER AFFILIATION**	University of Calgary
**REVIEWER CONFLICT OF INTEREST**	I am a volunteer member of a national guideline committee that has issued recommendations against prostate cancer screening.
**DATE REVIEW RETURNED**	17-May-2024

**GENERAL COMMENTS**	Thank you for the opportunity to review this manuscript which describes a study protocol for a randomized online trial comparing

	various labels for low grade prostate cancer. The study has described will recruit healthy adult Australians both women and men and conduct an online thought experiment as to how they perceive and would expect to be managed based on varying descriptions of a early stage potentially indolent prostate cancer lesion.<br>The study protocol is well written and has received ethics approval. The strengths of the study include add a good statistical power very well developed outcome measures and a very high likelihood of study completion. Additional strength includes code design of the study with members of the public.<br>The underline premise and rationale for this study is that at the present time 10s of thousands of men if not more globally are suffering the consequences of prostate cancer screening with the prostate specific antigen blood test. While rates have diminished given be widening perception that harms outweigh benefits, testing still continues resulting in men being diognosed with range of tumors but this includes low grade lesions which would almost certainly have never come to light had the test never been done in the 1st place. Unfortunately these men undergo treatment which can result in disfiguring and severe complications including erectile dysfunction and urinary incontinence.<br>I believe therefore that it is a very appropriate question to see if there is a way to frame this diagnostic finding based on a prostate cancer biopsy which would mitigate these harms by communicating to patients that in fact what has been discovered may actually be of little or no consequence.<br>Just two comments to consider. I don't think I am seeing the term watch end wait in the manuscript whereas active surveillance is being used. I do wonder if they are synonymous and if the former may provide some additional reassurance to men diagnosed with low grade prostate cancer. The other comment I have relates to instructions received in regards to this study protocol review which fails to make clear where the study stands at the moment in terms of embarking on recruitment and what timelines are associated with study completion.<br>Overall I believe this study protocol merits publication. In addition to being registered in a trials registry, peer reviewed publication would have the benefit of prompting scientists interested in reducing overdiagnosis from trialing this work in languages other than English.

VERSION 1 – AUTHOR RESPONSE

Reviewer: 1

1. This study delves into the impact of different diagnostic labels for low-risk prostate cancer on management choice and anxiety level among patients and their families. Overall, the study protocol exhibits innovative design, elucidates clear research concepts, presents comprehensive plans, and is both reasonable and feasible in its approach.

Response: Thank you for your supportive comments.

Reviewer: 2

1. 75% of prostate cancers that are diagnosed are low risk and over diagnosis is a serious problem. Consequently, the psychological impact of the diagnosis can in some cases be the major health impact on a man and his family. Ways of reducing this impact are welcome. The study has taken a co-design approach, identifying wordings that are used by Clinical Advisors and Consumers and then using a randomised trial to examine the impacts of the different terms. It is welcome to see the involvement of wives in this study as in our experience they and the wider family (especially daughters) often take the lead in helping patients with prostate cancer decide on their treatment options. Overall this seems a large well thought out study and I look forward to seeing its findings in due course.

Response: Thank you for your supportive comments.

2. The introduction outlines the background to the study. On lines 116 and 117 I was confused as whether Gleason Group reflected the prostate cancer grade group or the Gleason Score choice? This should be clarified as to which had the lower preference for treatment. **Response:** We have edited this for clarity.

Changes

Page 6, lines 115-117: "Prostate Cancer Gleason Score 6 (measured on a scale from 2 to 10), vs Prostate Cancer Gleason Group 1 (measured on a scale from 1 to 5), vs Indolent Lesion of Epithelial Origin ('IDLE'). Authors found that participants in the 'Prostate Cancer Gleason Group 1' label had a lower preference for immediate definitive treatment"

3. The methods should indicate how patients are recruited and how any bias will be mitigated. The authors say "Australian community members" will be randomised. Everyone is a community member. Are/have the authors advertised for participants or invited patients from a register or other source? How have these invitations been made? Facebook, TV, radio? I am not sure what they mean by "participants will be recruited via Qualtrics? " How have they ensured minority groups, low SES etc are included?

Response: We have added additional text to clarify these points.

Changes

Page 7, line 144: "Two separate online randomised studies of Australian residents will be run for men and for women."

Page 8, lines 166-169: "Participants will be recruited by the market research provider Qualtrics™, from their pre-arranged pool of respondents who have agreed to be contacted to respond to surveys[36]. Quotas will be used to ensure that approximately 50% of respondents are without tertiary education and the sample includes people from all Australian states and territories."

4. One concern I have that is not explained is the age boundaries of the study. While it is clear men under 50 are excluded, is there an upper age limit? After all the likelihood of active treatment, or active surveillance in those over 75 years of age is small. These patients have very different risk profiles with regards their risk of developing symptomatic cancer or dying from it. Also there are a diverse number of active surveillance strategies being used with increasing use of MRI. Certainly MRI in our jurisdiction is not available to all, and repeated biopsies are often off putting to patients (Ong WL). Have the authors considered stratified randomisation by age group of the cancer patients? Or by other factors e.g to ensure those whose first language is not English respond in a similar way to the different terms?

Ong WL, Thangasamy I, Murphy D et al. Large variation in conservative management of low-risk prostate cancer in Australia and New Zealand. BJU Int. 2022 Jun; 130(Suppl 1): 17–

19.
Response: We do not have an upper age limit on eligibility to participate in the study, and did not use stratified randomisation by age group or language. We are collecting data on these however, and will report effects across different characteristics. We agree that there are diverse approaches to active surveillance, including use of MRI and repeat biopsy. Our inclusion of “PSA monitoring” as well as “active surveillance” is intended to allow participants a less intensive monitoring option if that is their preference. We now include text that effects across important participant characteristics will be reported.

Changes

Page 12, lines 259-263: “We will estimate unadjusted and adjusted effects using the relevant regression model, and will present these across important patient characteristics, including: age, state or territory, relative socioeconomic disadvantage (postcode based), remoteness (postcode based), education, language spoken at home, family member with prostate cancer diagnosis, and other participant characteristics in Table 2.”

5. How have risk estimates been collated?? Eg 3% developing metastases in 15 years seems low (Bill-Axelson A) and may falsely alter the patient choice of treatment. The estimates seem low, especially for what would be expected for older patients.
Bill-Axelson A, Holmberg L, Garmo H et al. Radical Prostatectomy or Watchful Waiting in Prostate Cancer — 29-Year Follow-up. N Engl J Med. 2018;379:2319-2329 DOI:

10.1056/NEJMoa1807801.

Response: The risk estimates for developing metastases we have used (6% for PSA monitoring/active surveillance and 3% for prostatectomy and radiotherapy) are from the two randomised trials that included a majority with PSA-detected prostate cancer: PIVOT (US study) and ProtecT (UK study). The results from these trials are more applicable to our hypothetical clinical scenario than those from the Scandinavian Prostate Cancer Group Study Number 4 (SPCG-4) trial suggested by the reviewer, which was done before widespread PSA testing (Bill-Axelson NEJM 2018). Specifically, we sourced the risk estimates from these two trials as follows:

Wilt NEJM 2012: from Table S6, among men with low-risk cancers (Gleason Score ≤6, PSA < 10 and Tumour Stage T1a, T1b, T1c or T2a), the risk of bone metastasis at 12 years was 4.9% in the observation group, and 0% in the prostatectomy group.

Hamdy NEJM 2023: from the section “Patients and Risk Stratification, there were n=1243 men with grade group 1 cancers who had 15 years follow up data (1610 x 77.2%). From Table S5, n=53 men with grade group 1 developed metastases, meaning that 4.3% (53/1243) of men with grade group 1 cancer across randomised groups developed a metastasis. From Table 1, among the full trial sample, compared to active monitoring the hazard radio for clinical progression was 0.36 for prostatectomy and 0.35 for radiotherapy. Assuming the same relative effects for men with grade group 1 cancers, approximately 6% of men in the active monitoring group and 3% in the prostatectomy and radiotherapy groups developed a metastasis.

Changes

Page 9, lines 188-189: "as reported in the two randomised controlled trials (RCTs) that included a majority with PSA-detected cancer"

Page 27, footnote for Figure 3: "1. Risk estimates are based on long term follow up in the PIVOT[18-20] and ProtecT RCTs.[12 21 22]

6. Also have the authors considered the consistency of the information to be given with what is on line? For instance if you search using the three different terms you are directed to different websites. This also depends on whether you filter your online search to Australia. After all if the different terms are used in providing information it is likely patients/family will search for these terms to better understand their condition and options. By searching "low risk prostate cancer" you are directed to a number of American sites e.g. UCLA – if au is added to the search you are directed to the Prostate Cancer Foundation of Australia. How people interpret different terms such as cancer, neoplasm or lesions is likely going to be influenced from what is available online and this point maybe something for the discussion. **Response:** We agree that information available online could influence how people interpret the different terms. Although we have not included discussion on this point in the protocol paper, this may be something we include and discuss in the reports of the experiments.

Reviewer: 3

1. Thank you for the opportunity to review this manuscript which describes a study protocol for a randomized online trial comparing various labels for low grade prostate cancer. The study has described will recruit healthy adult Australians both women and men and conduct an online thought experiment as to how they perceive and would expect to be managed based on varying descriptions of a early stage potentially indolent prostate cancer lesion.
The study protocol is well written and has received ethics approval. The strengths of the study include add a good statistical power very well developed outcome measures and a very high likelihood of study completion. Additional strength includes code design of the study with members of the public.

The underline premise and rationale for this study is that at the present time 10s of thousands of men if not more globally are suffering the consequences of prostate cancer screening with the prostate specific antigen blood test. While rates have diminished given be widening perception that harms outweigh benefits, testing still continues resulting in men being diognosed with range of tumors but this includes low grade lesions which would almost certainly have never come to light had the test never been done in the 1st place.

Unfortunately these men undergo treatment which can result in disfiguring and severe complications including erectile dysfunction and urinary incontinence.

I believe therefore that it is a very appropriate question to see if there is a way to frame this diagnostic finding based on a prostate cancer biopsy which would mitigate these harms by communicating to patients that in fact what has been discovered may actually be of little or no consequence.

Overall I believe this study protocol merits publication. In addition to being registered in a trials registry, peer reviewed publication would have the benefit of prompting scientists interested in reducing overdiagnosis from trialling this work in languages other than English.

Response: Thank you for your supportive comments. We agree that trialling this work in other languages may be a very worthwhile endeavour.

2. I don't think I am seeing the term watch and wait in the manuscript whereas active surveillance is being used. I do wonder if they are synonymous and if the former may provide some additional reassurance to men diagnosed with low grade prostate cancer.
Response: We used the terms "PSA monitoring" and "Active surveillance" to indicate varying intensity of monitoring included in the conservative management choice. As data collection is now completed, we cannot change the terms used to describe the management choices in the experiments. However, we have added the term "watchful waiting" to the Introduction.

Changes

Page 5, line 94: "While randomised controlled trials have demonstrated clinical effectiveness of observation with additional care for symptomatic disease spread ("watchful waiting"), or active surveillance using PSA monitoring with delayed intervention with surgery or radiation[18-22]"

3. The other comment I have relates to instructions received in regards to this study protocol review which fails to make clear where the study stands at the moment in terms of embarking on recruitment and what timelines are associated with study completion.
Response: We have now finished data collection and manuscripts to report on the study are in preparation.
